# Holo-Seq: single-cell sequencing of holo-transcriptome

**DOI:** 10.1186/s13059-018-1553-7

**Published:** 2018-10-17

**Authors:** Zhengyun Xiao, Guo Cheng, Yang Jiao, Chen Pan, Ran Li, Danmei Jia, Jing Zhu, Chao Wu, Min Zheng, Junling Jia

**Affiliations:** 1 Life Sciences Institute and Innovation Center for Cell Signaling Network, Zhejiang University, Hangzhou, 310058 Zhejiang People’s Republic of China; 2 Collaborative Innovation Center for Diagnosis and Treatment of Infectious Diseases, Zhejiang University, Hangzhou, 310003 Zhejiang People’s Republic of China; 3 State Key Laboratory for Diagnosis and Treatment of Infectious Diseases, The First Affiliated Hospital, Zhejiang University, Hangzhou, 310003 Zhejiang People’s Republic of China; 4 Beijing Ming-tian Genetics Ltd., Beijing, 100070 People’s Republic of China

**Keywords:** Single cell, mRNA-Seq, Total RNA-Seq, Anti-sense transcript, Small-RNA-Seq, Super-enhancer, Liver cancer

## Abstract

Current single-cell RNA-seq approaches are hindered by preamplification bias, loss of strand of origin information, and the inability to observe small-RNA and mRNA dual transcriptomes. Here, we introduce a single-cell holo-transcriptome sequencing (Holo-Seq) that overcomes all three hurdles. Holo-Seq has the same quantitative accuracy and uniform coverage with a complete strand of origin information as bulk RNA-seq. Most importantly, Holo-Seq can simultaneously observe small RNAs and mRNAs in a single cell. Furthermore, we acquire small RNA and mRNA dual transcriptomes of 32 human hepatocellular carcinoma single cells, which display the genome-wide super-enhancer activity and hepatic neoplasm kinetics of these cells.

**Electronic supplementary material:**

The online version of this article (10.1186/s13059-018-1553-7) contains supplementary material, which is available to authorized users.

## Background

No two cells are the same, which can be largely reflected at the transcriptional level, and single-cell RNA-Seq is an ideal approach to demonstrate the differences across cells [1–3]. Although many methods have been established to probe transcriptomes from single cells [4–12], more efforts are needed to acquire the same quantitative accuracy and a complete strand of origin information provided by bulk RNA-Seq. Moreover, an approach to observing both small RNA and mRNA transcriptomes simultaneously in a single cell is still lacking. These limitations are major hurdles for detecting the subtle differences in transcriptomes, decoding non-coding information, understanding miRNA regulating networks and probing genome-wide super-enhancer activity in single cells, which are all crucial for the identification and characterization of cell types, states, and rare cellular phenotypes [13–15].

Current bulk RNA-Seq approaches are well developed and highly accurate for obtaining mRNA, small RNA, and non-coding RNA information [13, 16–18]. We reason that adapting the conventional bulk RNA-Seq approach to the single-cell level will be an ideal and applicable solution to overcome the hurdles that currently limit single-cell RNA-Seq methods.

Here, we introduce a single-cell holo-transcriptome sequencing (Holo-Seq) method that uses in vitro transcribed RNAs as the carrier to protect against cellular RNA loss during conventional library construction procedures. Then, restriction endonucleases are used to remove carrier-generated cDNA fragments from sequencing libraries. We successfully used Holo-Seq to adapt bulk RNA-Seq approaches, such as poly-A selection mRNA-Seq, directional total RNA-Seq, and small RNA-Seq, to single-cell level. As expected, Holo-Seq attained the same quantitative accuracy as bulk mRNA-Seq. More importantly, Holo-Seq can retain a complete strand of origin information and simultaneously probe small RNA and mRNA transcriptomes in a single cell.

Furthermore, we applied Holo-Seq to probe small RNA-mRNA dual transcriptomes from 32 single cells isolated from a human hepatocellular carcinoma. We found three expression-based subpopulations (Exp-subpopulations) with six featured transcript groups and three super-enhancer-based subpopulations (SE-subpopulations). We also inferred a potential hepatic neoplasm kinetics model showing that (1) HCC malignant transition couples with genome-wide super-enhancer remodeling and (2) the downregulation of mitochondrial activity and the upregulation of both tumor suppressor miRNA and oncomiRs happen at the early stage of malignant transition before activating tumorigenesis signaling pathways.

## Results

### Holo-Seq has same accuracy and coverage as bulk mRNA-Seq

We utilized the T7 promoter to transcribe carrier RNA from an artificial DNA fragment (Additional file 1: Figure S1). No reads (50 bp) generated from the carrier DNA, which contains a Not I site every 20–30 bp (Additional file 1: Figure S1), could be mapped to the mouse or human genome using TopHat (2 mismatches allowed). We added carrier RNA immediately after single-cell lysis and constructed the mRNA sequencing library following the manufacturer’s protocol after poly-A selection (NEB 7530; Additional file 1: Figure S2). To remove the carrier-oriented cDNA fragments from the sequencing library, we digested the library with Not I before sequencing (Additional file 1: Figure S1). Two thousand six hundred seventy-one human genes and 1779 mouse genes contain Not I site and most of them (> 90%) contain no more than 2 sites (Additional file 2: Table S1, S2). Because Holo-Seq fragments mRNAs to generate short cDNAs (200–300 bp) before library construction, Not I digestion only disrupts limited reads and causes no significant bias (Additional file 2: Table S1, S2; Additional file 1: Figure S3 a, b). We also provided an alternative to removing the carrier cDNA fragments using in vitro CRISPR/Cas9 digestion if the restriction enzyme was not accepted (Additional file 1: Figure S1; S3 c, d). The carrier removal efficiency (> 85%) is high (Additional file 2: Table S3), and the mapping results of Holo-Seq libraries are reasonable (Additional file 2: Table S3), which indicate that the carrier does not significantly interfere with the library construction and do not significantly increase the cost (Additional file 2: Table S4).

**Fig. 1:**
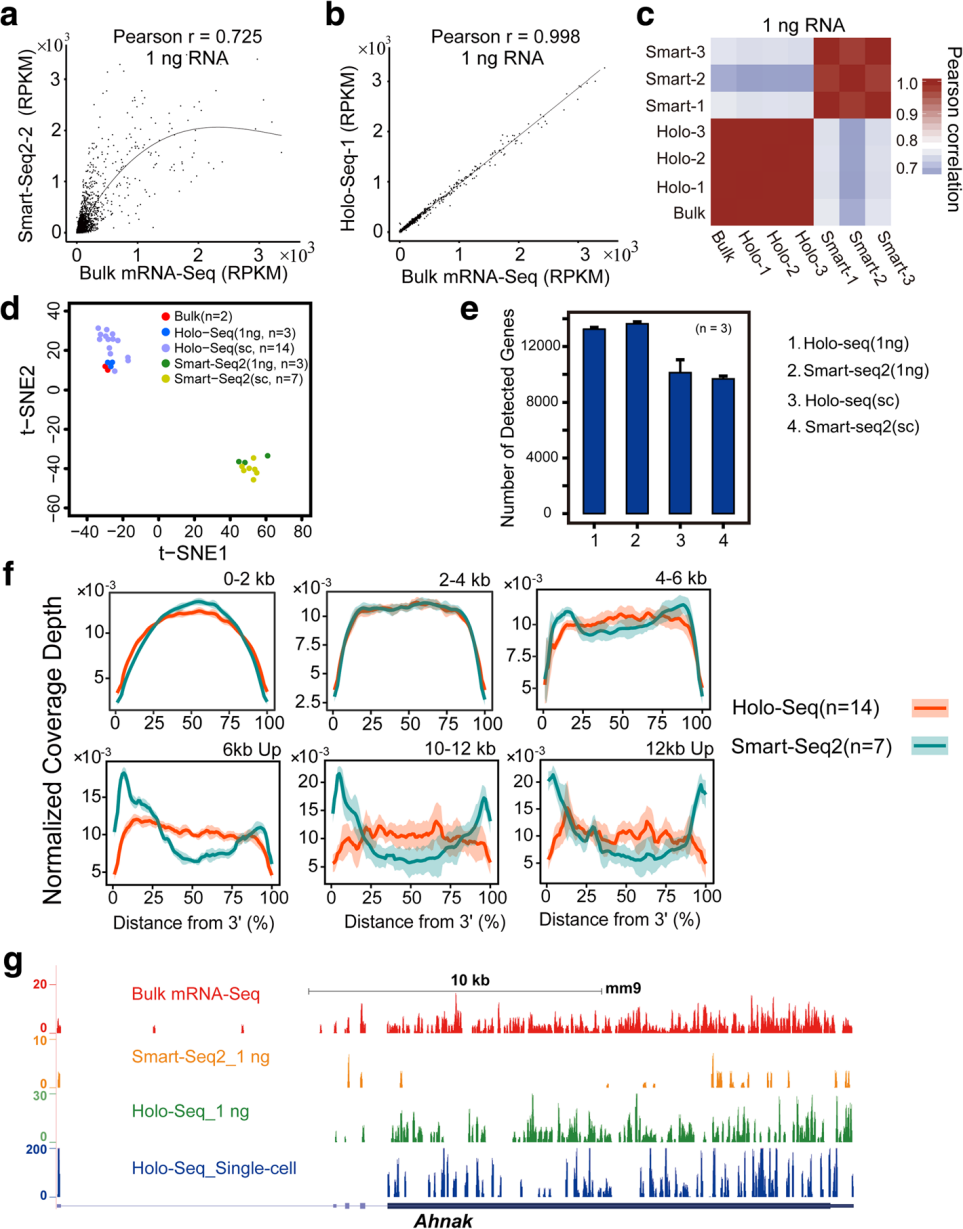
Holo-Seq profiles mRNA with the same accuracy and coverage as bulk mRNA-Seq. **a** An RPKM scatterplot of expressed genes between Smart-Seq2 and bulk mRNA-Seq. 1 ng of mESC total RNA was used. **b** An RPKM scatterplot of expressed genes between Holo-Seq (mRNA) and bulk mRNA-Seq. 1 ng of mESC total RNA was used. **c** Pearson correlation coefficient heat map of the mRNA profiles generated from 1 ng of total RNA by Holo-Seq (mRNA), Smart-Seq2, and bulk-mRNA-Seq. Three biological replicates were performed. **d** t-SNE analysis of mESCs (bulk-mRNA-Seq), mESC single cells (Holo-Seq and Smart-Seq2), and 1 ng mESCs total RNA (Holo-Seq and Smart-Seq2). Principal components were used as inputs. **e** Comparison of the number of genes detected by Holo-Seq and Smart-Seq2 from 1 ng mESC total RNA and mESC single cells at same mapped depths (6.8 M and 3.2 M). **f** Comparison of the read coverage across transcripts of different lengths between Holo-Seq and Smart-Seq2 from mESCs single cells. The read coverage over the transcripts is displayed along with the percentage of the distance from their 3′ end. Shaded regions indicate the standard deviation (SD). **g** The plot of the signals of *Ahnak* detected from mESCs (bulk mRNA-Seq), 1 ng mESC total RNA (Holo-Seq and Smart-Seq2), and a mESCs single cell (Holo-Seq) on the University of California Santa Clara (UCSC) gene browser

To compare the accuracy of Holo-Seq and Smart-Seq2, we generated Holo-Seq and Smart-Seq2 libraries from 1 ng diluted total RNA (total RNA from approximately 100 mESCs). This strategy was based on a previous work showing that nanogram-diluted sampling of total RNAs could satisfactorily approximate bulk total RNA distribution and be stably profiled by Smart-Seq2 [5], and we used conventional bulk mRNA-Seq (800 ng of total RNAs input) as the benchmark. By comparing the reads per kilobase per million mapped reads (RPKM) values of approximately 14,000 genes, the accuracy of Holo-Seq (Pearson *r* 0.997–0.998) was significantly better than that of Smart-Seq2 (Pearson *r* 0.725–0.779) (Fig. 1a, b, c; Additional file 1: Figure S4, S5). Next, we visualized the data from Holo-Seq and Smart-Seq2 in two dimensions using t-distributed stochastic neighbor embedding (t-SNE) and hierarchical cluster analysis (HCA). As expected, the data of Holo-Seq (1 ng) and Holo-Seq (SC) tightly surround the data of bulk mRNA-Seq, whereas the data of Smart-Seq2 (1 ng) and Smart-Seq2 (SC) are separated from them (Fig. 1d; Additional file 1: Figure S6). The results show again that the accuracy of Holo-Seq is significantly better than that of Smart-Seq2. We also compared the Holo-Seq with Smart-Seq2 coupled with Nextera XT library construction workflow and got similar results (Additional file 1: Figure S7). This suggests that the library construction step does not cause the low accuracy of Smart-Seq2. In addition, the sensitivity of Holo-Seq and Smart-Seq2 for probing poly-A RNAs are comparable. Holo-Seq consistently detected 13,258 ± 128 genes from 1 ng mESC total RNA and 9994 ± 899 genes from single mESC cells (Fig. 1e).

The complexity of the library is measured by the number of unique mapped reads which is decided by the unique broken patterns of cDNA during the fragmentation step. The high complexity SMART-Seq is artificial because SMART-Seq preamplifies the large cDNAs before the fragmentation step that can lead to more broken patterns of cDNAs. As expected, although the saturated point of Smart-Seq2 (single cells) is higher than Holo-Seq (single cells) (Additional file 1: Figure S8 a, b, c), Smart-Seq2 detected fewer genes than Holo-Seq at the same exome-mapped depth (Additional file 1: Figure S8 a, b, c).

Because the PCR efficiency of long DNA fragments is markedly lower than that of short fragments, the preamplification step of Smart-Seq2 inevitably causes coverage bias, especially for long cDNAs. Unsurprisingly, the central regions of long cDNAs are less covered by Smart-Seq2, and once the cDNA is longer than 10 kb, the central region is barely covered (Fig. 1f, g). Owing to the carrier RNA, Holo-Seq can directly construct the library following the conventional mRNA-Seq pipeline without preamplification, which enables uniform coverage for cDNAs of all lengths (Fig. 1f, g).

### Holo-Seq accurately profiles total RNAs with a complete strand of origin information

**Fig. 2:**
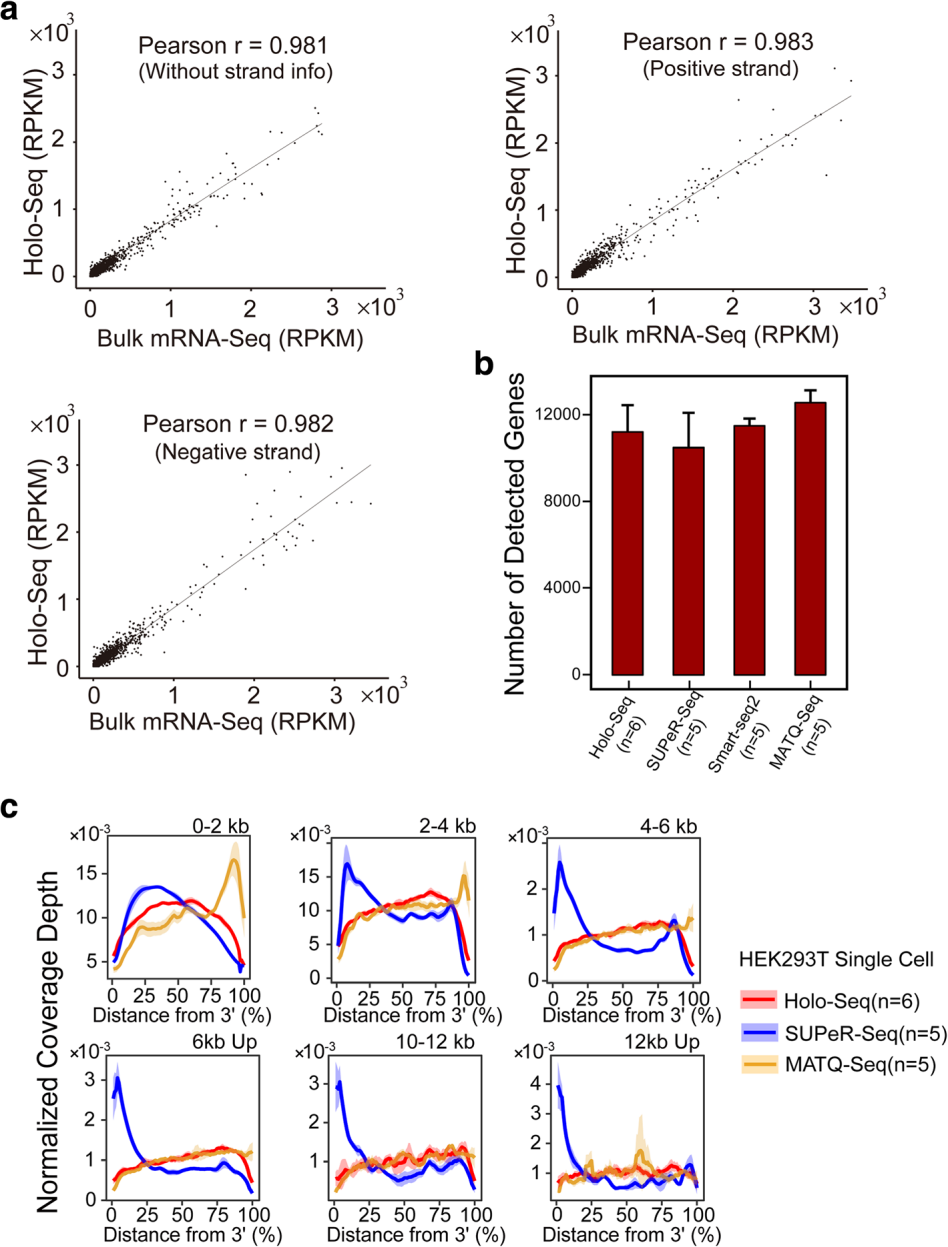
Holo-Seq accurately profiles total RNAs with a complete strand of origin information from single cells. **a** RPKM scatterplots of expressed genes between the combined dataset (total RNA with a complete strand of origin information from 10 mESCs single cells) and a directional bulk mRNA-Seq. **b** Comparison of the detected gene number in HEK293T single cells at the maximum exome-mapped depth of MATQ-Seq (UMI labeled reads) and 1.2M unique exome-mapped depth of Holo-Seq, SUPeR-Seq, and Smart-Seq2. **c** Read coverage across transcripts of different lengths of three methods in HEK293T single cells. The read coverage over the transcripts is displayed along with the percentage of the distance from their 3′ end. Shaded regions indicate the standard deviation

A strand of origin information plays an important role in accurately quantifying gene expression for approximately 19% of genes in which overlapping genomic loci are transcribed from opposite strands [19]. Additionally, RNAs transcribed from the antisense strands (either introns or coding regions) contain important information defining the type and stage of cells [20]. Current single-cell methods cannot retain a complete strand of origin information. To overcome this challenge, we adapted the conventional directional total RNA-Seq pipeline to the single-cell level (Additional file 1: Figure S9). We probed total RNA with a complete strand of origin information from 10 individual mESCs. The carrier removal efficiency (> 85%) is high (Additional file 2: Table S3), and the mapping results of Holo-Seq libraries are reasonable (Additional file 2: Table S3). Because we did not employ any rRNA removing strategy, these libraries have a high percentage of rRNA mapped reads (50–60%). To evaluate the accuracy, we first combined an equal number of mapped reads from each of the ten cells and then compared the RPKM values of expressed genes between the combined data and a directional bulk mRNA-Seq. Because the mRNAs from 10 randomly selected mESCs approximate the bulk mESCs [21], the high correlation values indicate that the quantitative accuracy of Holo-Seq probing total RNAs with a complete strand of origin information is comparable to that of the bulk directional mRNA-Seq (Fig. 2a).

Next, we compared the coverage and the sensitivity between Holo-Seq and two published approaches (SUPeR-Seq and MATQ-Seq) that can also probe total RNAs from single cells [22, 23]. Holo-Seq total RNA pipeline can detect 11126 ± 1447 genes from HEK293T single cells, which is comparable to that of SUPeR-Seq, MATQ-Seq, and Smart-Seq2 [6] (Fig. 2b). With the same exome-mapped reads, SUPeR-Seq detected fewer genes than Holo-Seq, but MATQ-Seq detected more genes than Holo-Seq (Additional file 1: Figure S8 d). Although both SUPeR-Seq and MATQ-Seq preamplify the large cDNAs before the fragmentation step, the special preamplification strategy of MATQ-Seq promotes its performance [23]. Both Holo-Seq and MATQ-Seq show uniform coverage across the transcripts over 2 kb without significant bias, whereas SUPeR-Seq has apparent 5′- or 3′-end bias. Interestingly, both SUPeR-Seq and MATQ-Seq show significant bias across the 0–2 kb transcripts which can be uniformly covered by Holo-Seq total RNA pipeline (Fig. 2c).

**Fig. 3:**
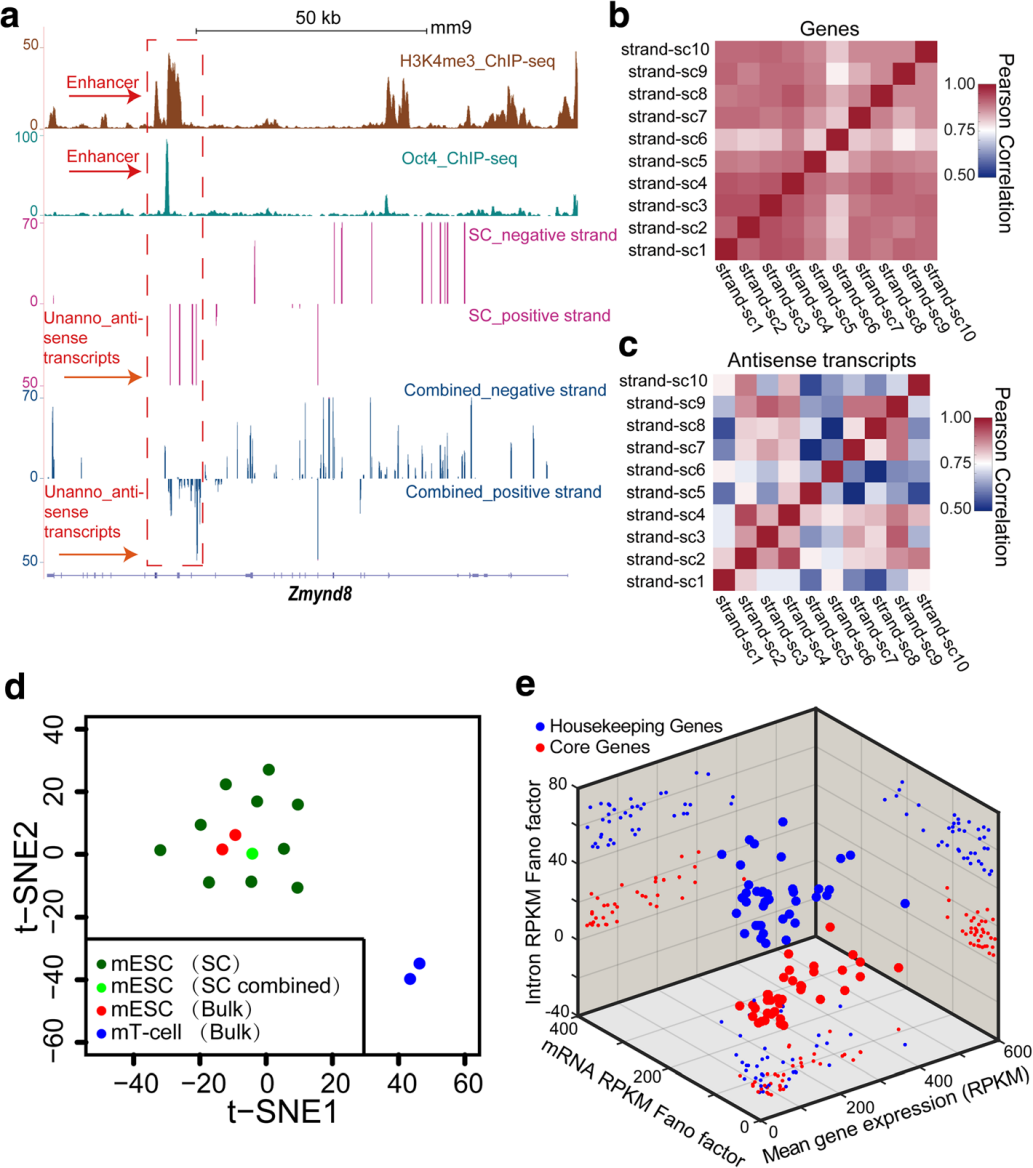
Strand of origin information of Holo-Seq presents intronic and antisense transcripts in single cells. a The plot of the signal from an unannotated antisense transcript inside Zmynd8 locus. The enhancer region is indicated by both Oct4 and H3K4me3 ChIP-Seq signal clusters. **b**, **c** Pearson correlation heat maps of coding genes (**b**) and antisense transcripts (**c**) abundance profiled by Holo-Seq from 10 mESC single cells. **d** t-SNE analysis of mESCs (bulk directional RNA-Seq), mESCs single cells (Holo-Seq), mESCs single cells combined (Holo-Seq), and mouse bone marrow T cells (bulk directional RNA-Seq) using their antisense transcripts. **e** 3D scatterplot of the gene expression level, intron RPKM Fano factor, and mRNA RPKM Fano factor of a core gene group (red dots) and a housekeeping gene group (blue dots) in 10 mESC single cells (Additional file 3: Table S6). With similar gene expression level (mRNA RPKM), the core gene group has significantly lower pre-mRNA bursting than that of the housekeeping gene group

Antisense transcripts, many of which lacking annotations, are important regulators of gene transcription, translation, and RNA degradation and may contribute to self-regulatory circuits that allow genes to regulate themselves expression [24]. Using a complete strand of origin information acquired by Holo-Seq total RNA pipeline, we identified 301 (66 known, 235 unannotated) antisense transcripts and analyzed their abundance in ten individual mESCs (Additional file 3: Table S5; Additional file 1: Figure S10). For example, the antisense transcript from Zmynd8, at an Oct4-occupied enhancer, has been shown to be an important regulator of mESCs self-renewal [25] (Fig. 3a; Additional file 1: Figure S10 b). A strand of origin information can also help accurately count reads from genes with overlapping genomic loci that are transcribed from opposite strands (Additional file 1: Figure S11). Next, we found that antisense transcript expression is more diverse than the mRNA expression in ten individual mESCs (Fig. 3b, c; Additional file 1: Figure S10, S12) and can efficiently identify mESCs from mouse bone marrow-originated T cells (Fig. 3d). Overall, the antisense transcript information acquired by Holo-Seq total RNA pipeline is valuable for characterizing the cell type or state at single-cell resolution.

Pre-mRNA abundance can be measured using reads mapped to introns. A complete strand of origin information excluding antisense strand reads enabled us to accurately count the intron-mapped reads. In the ten mESCs, we assessed the expression level, pre-mRNA bursting Fano factor (represented by the ratio of variance to mean), and mRNA bursting Fano factor of each expressed gene [23]. Although the expression levels and mRNA bursting were similar, the pre-mRNA bursting of some housekeeping genes was significantly higher than that of some core transcriptional factors (Fig. 3e; Additional file 1: Figure S13; Additional file 3: Table S6). This indicates that the RNA metabolism kinetics of these core genes is different from housekeeping genes, which could be the result of different underlying transcription, splicing, or degradation mechanisms [26–28].

### Holo-Seq simultaneously profiles small RNA and mRNA transcriptomes from single cells

A recent effort has established small RNAs, especially miRNAs, as the molecular identification and characterization of cell types and states at the single-cell level [29]. Here, we utilized Holo-Seq to adopt the conventional small RNA transcriptome pipeline to the single-cell level (Additional file 1: Figure S14). Since we did not find any Not I sites in known human and mouse miRNAs, tsRNAs, and snoRNAs, we still use Not I to remove the small-RNA carriers.

**Fig. 4:**
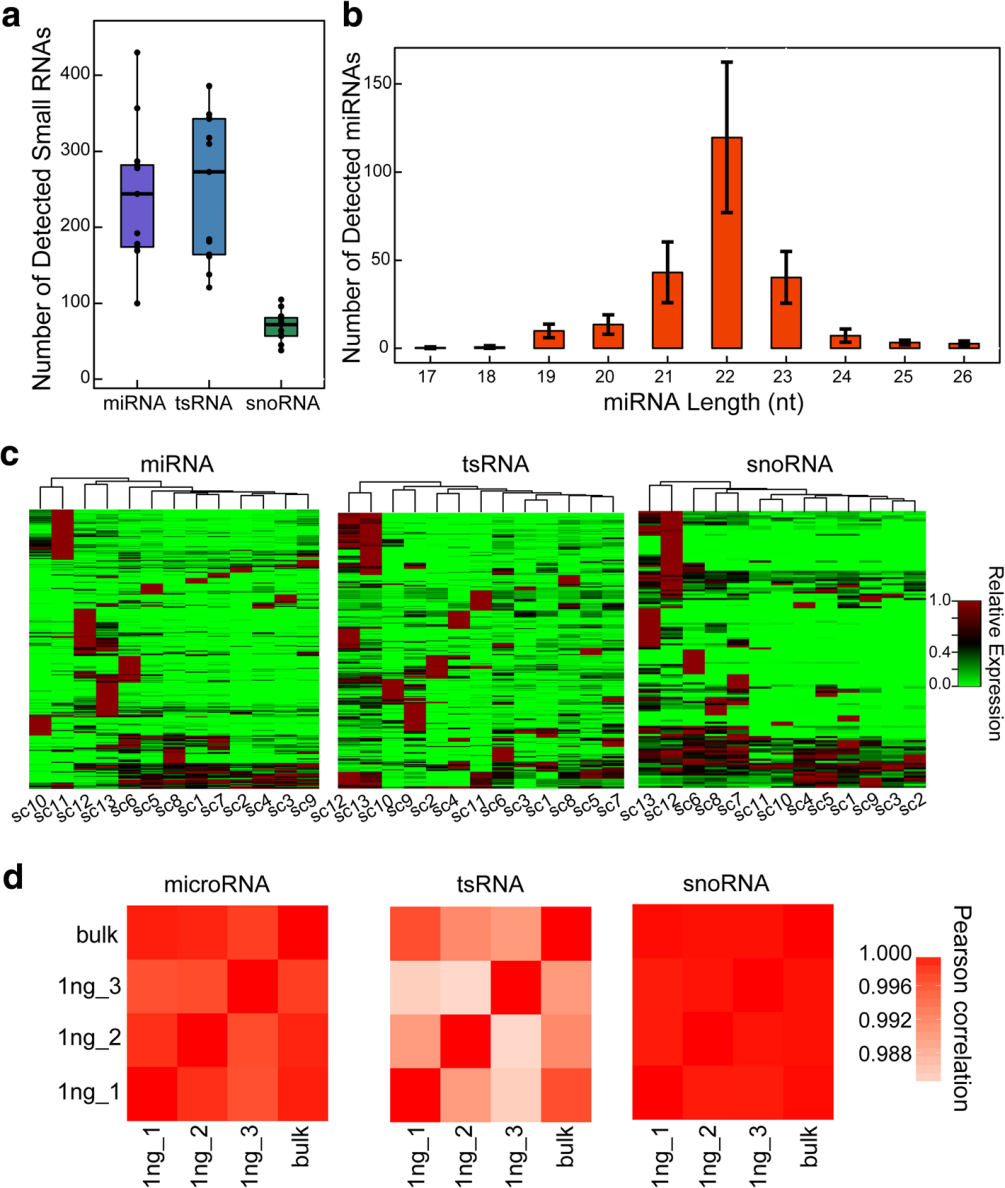
The characterization of small RNAs transcriptome by Holo-Seq. **a** Number of miRNAs, tsRNAs and snoRNAs detected in mESC single cells. **b** Size distribution of miRNAs profiled from mESC single cells. Error bars indicate the Standard Deviation (SD). **c** Heat map of the relative expression levels of miRNAs, tsRNAs and snoRNAs profiled from 13 mESC single cells. **d** Pearson correlation heat maps of miRNAs, tsRNAs, and snoRNAs abundance profiled from ZHBTc4 cells (bulk small-RNA-Seq) and 1 ng ZHBTc4 total RNAs (Holo-Seq)

We probed small RNAs from 13 mESC single cells, and the small RNAs were identified computationally (see the “Methods” section). On average, we captured 242 miRNAs, 252 tsRNAs, and 70 snoRNAs per cell (Additional file 3: Tables S7-S9; Fig. 4a). The saturation curve and sequencing depth of Holo-Seq (2–3 M mouse mapped reads) for probing miRNA are comparable with that of the previous study (~ 2 M human mapped reads) (Additional file 1: Figure S15; Additional file 2: Table S3) [29]. The length of detected miRNAs was approximately 22 nt (Fig. 4b), and the miRNA and tsRNA abundance profiles have higher heterogeneity than the snoRNA abundance profile (Fig. 4c), which is consistent with the previous study that miRNA and tsRNA can be more useful to display cell types and single-cell variations than snoRNAs [29]. To validate the quantitative accuracy of Holo-Seq small RNA pipeline, we probe small RNAs from 1 ng and bulk total RNA of ZHBTc4 cells. By comparing the RPM (reads per million mapped reads) values of approximately 389 small RNAs (snoRNA, tsRNA and miRNA), the accuracy of Holo-Seq is high (Fig. 4d; Additional file 1: Figure S16) (Pearson *r* 0.9904–0.9998).

Each miRNA likely downregulates multiple mRNAs, and each mRNA can also be targeted by multiple miRNAs to produce synergistic effects [30]. Thus, focusing on miRNA-mRNA networks rather than miRNAs can help decode miRNA functions at the single-cell resolution. However, current methods cannot observe small RNA-mRNA dual transcriptomes from single cells.

**Fig. 5:**
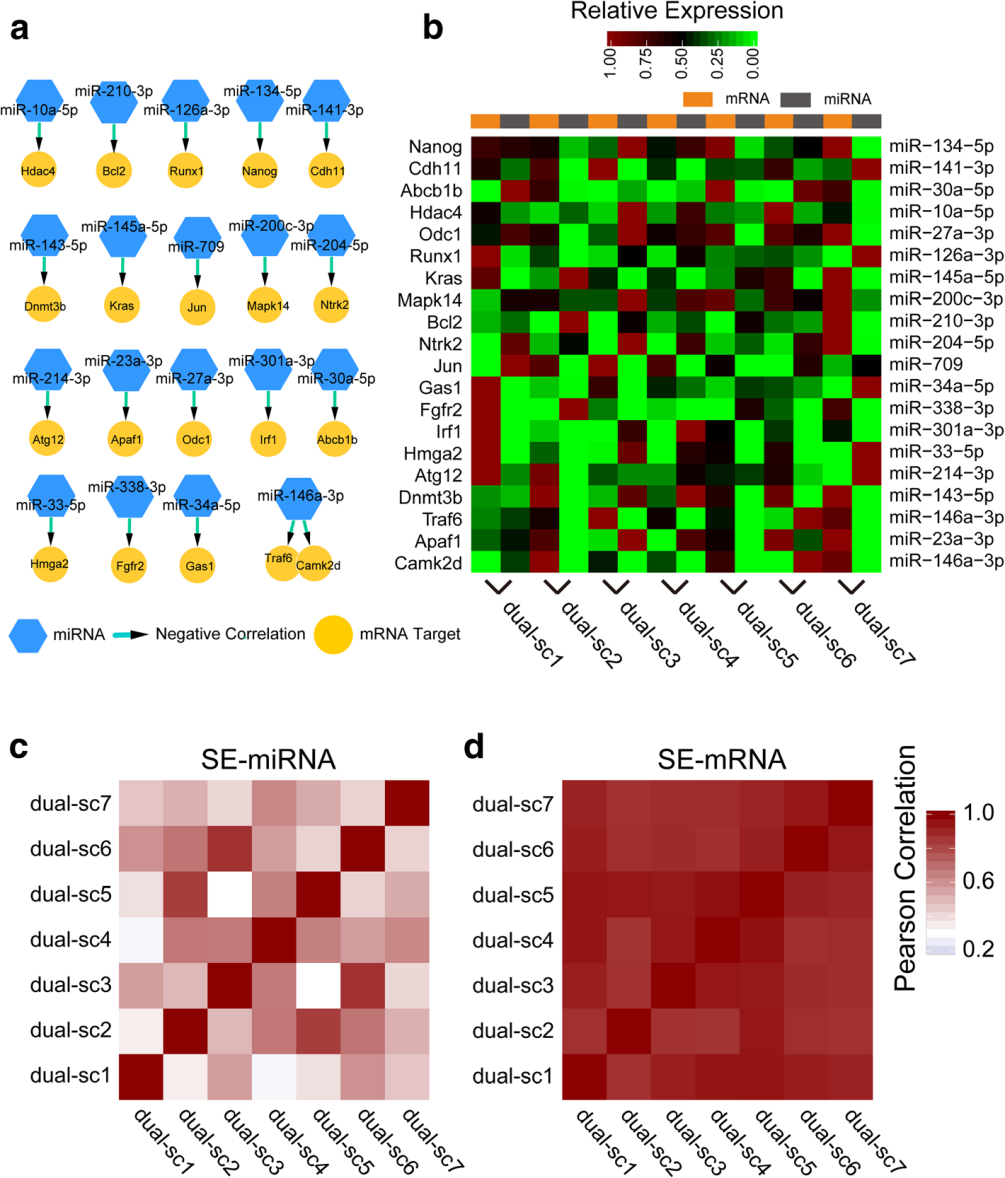
Holo-Seq profiles small RNA-mRNA dual transcriptomes from single cells. **a** miRNA-mRNA regulatory modules identified by small RNA-mRNA dual transcriptome sequencing from mESC single cells. **b** Heat map of the relative expression of miRNAs and their potential targeting mRNAs identified by small RNA-mRNA dual transcriptome sequencing from mESC single cells. **c**, **d** Pearson correlation heat maps of the genome-wide super-enhancer activity of seven mESC single cells

To profile the small RNA-mRNA dual transcriptomes, we performed a poly-A selection before implementation of the small RNA library construction pipeline. We successfully acquired dual transcriptome information from seven individual mESCs. As proof of principle that dual transcriptome information could facilitate understanding of the miRNA-mRNA regulatory networks, we pairwise compared the abundance of miRNAs and their potential targets (623 total pairs) identified in silico [31] (Additional file 3: Table S10). Further analyses revealed that 24 miRNA-mRNA pairs displayed significant negative correlations (*R* < 0; *P* < 0.05) of their abundance and presented heterogeneity in seven individual mESCs (Fig. 5a, b; Additional file 3: Table S10).

Super-enhancers are genomic regions comprising multiple enhancers that are collectively occupied by transcription factors to regulate the transcription of genes important for controlling and defining cell identity [32–34]. The abundance of super-enhancer-regulated genes can be used to measure super-enhancer activity. Similar to mRNAs, miRNAs can be regulated by super-enhancer as master miRNAs to orchestrate cell identity [14]. Using single-cell small RNA-mRNA dual transcriptomes, we can extract the abundance of super-enhancer-regulated master miRNAs and mRNAs (Additional file 3: Tables S11, S12) and better display genome-wide super-enhancer activity at single-cell resolution than using a solo transcriptome (miRNA or mRNA) (Fig. 5c, d; Additional file 1: Figure S17).

Together, the small RNA-mRNA dual transcriptome information not only helps validate the miRNA targets in vivo but also provides a novel perspective (super-enhancer) to explore cell heterogeneity.

### The small RNA-mRNA dual transcriptome sequencing of HCC single cells

**Fig. 6:**
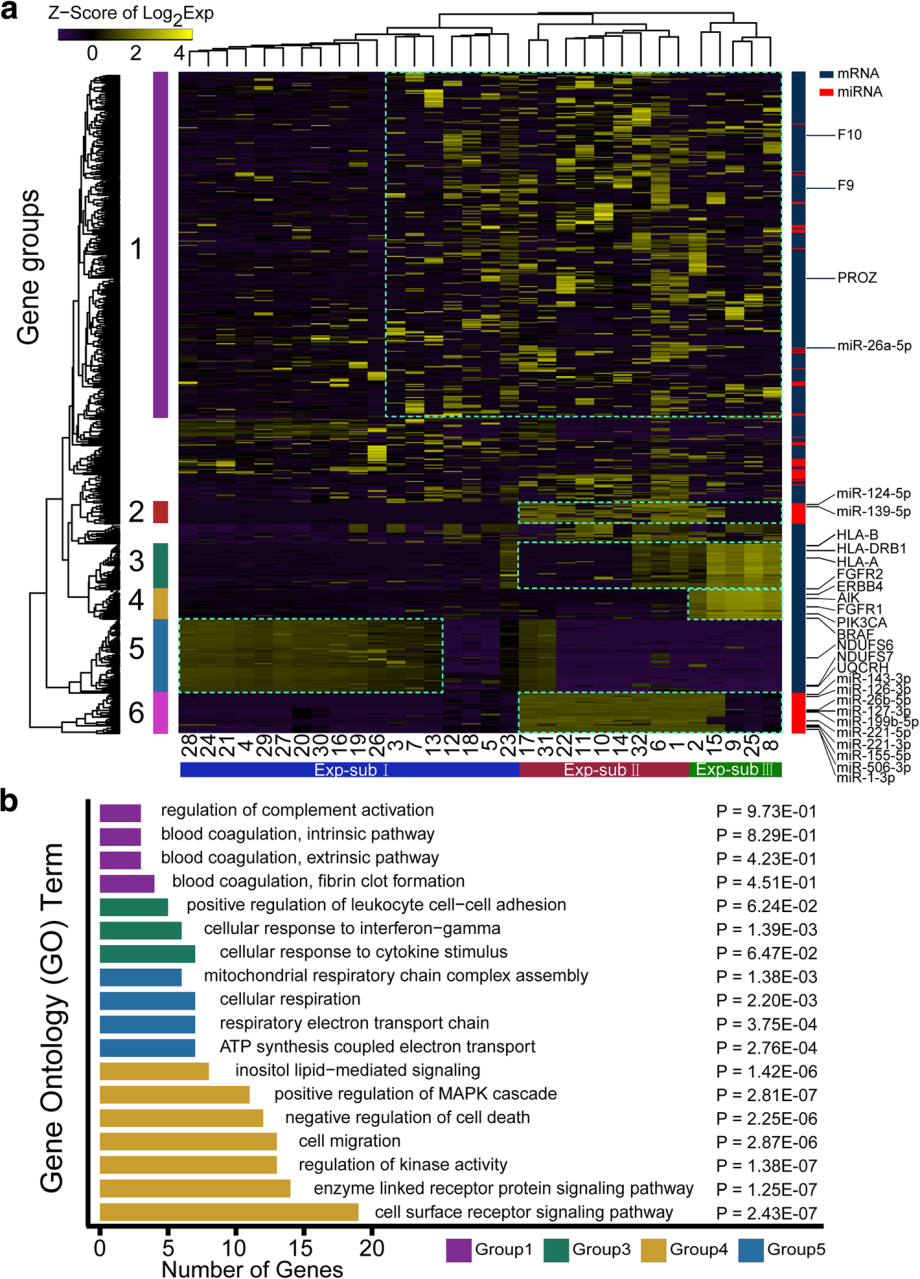
miRNA-mRNA dual transcriptome analyses of hepatocellular carcinoma (HCC) single cells. **a** Unsupervised hierarchical clustering analysis of 32 HCC single cells based on the top variant mRNAs and miRNAs (details in the “Methods” section). Three expression-based cell subpopulations (Exp-subpopulation) and six differential expressed gene groups were identified (dashed squares). Featured cancer-related genes are labeled on the right. **b** Featured cancer-related Gene Ontology (GO) terms in gene groups 1, 3, 4, and 5

**Fig. 7:**
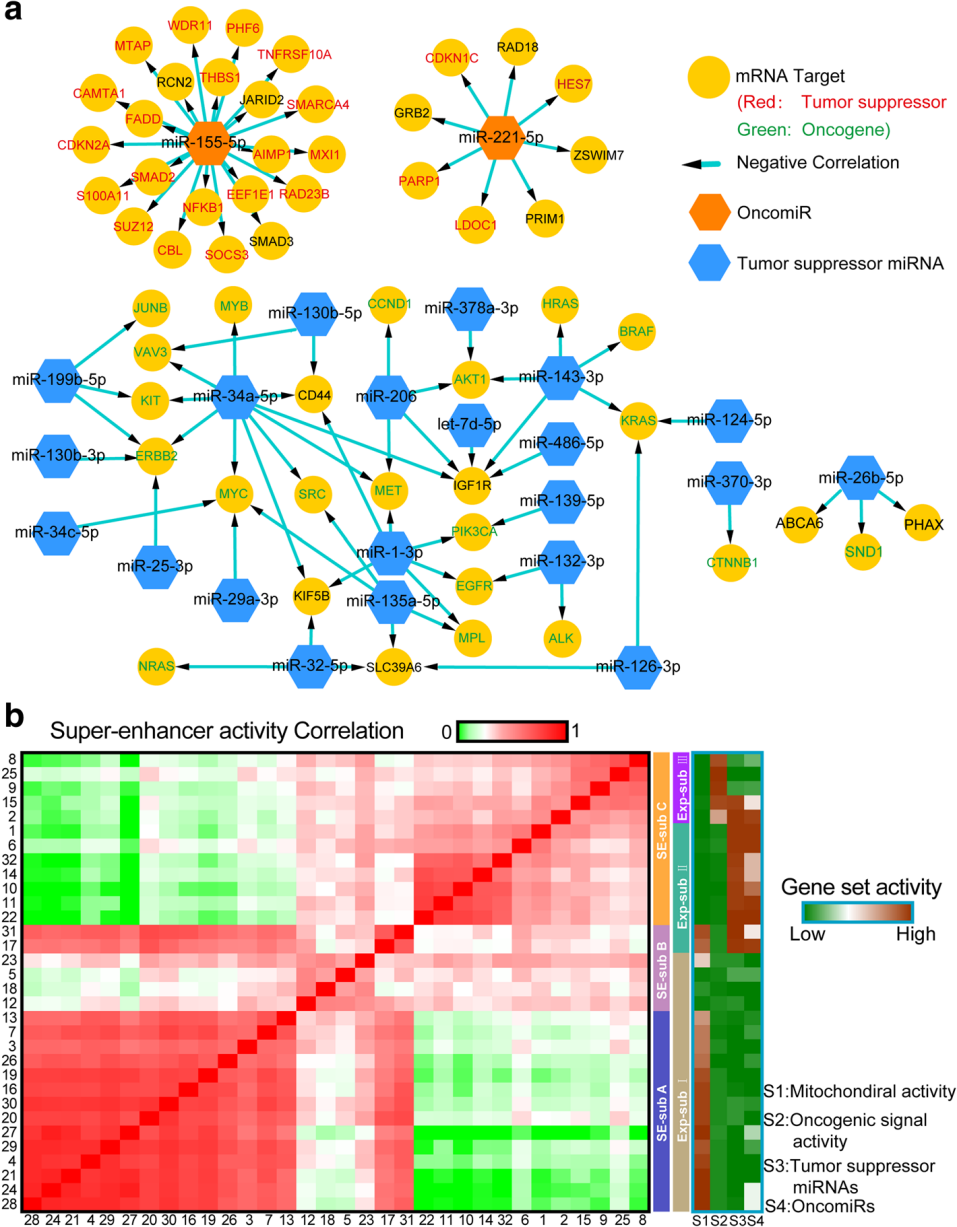
miRNA-mRNA regulatory modules and super-enhancer activity analysis of hepatocellular carcinoma (HCC) single cells. **a** Identified miRNA-mRNA regulatory modules related to tumorgenesis. **b** Correlation heat map of the genome-wide super-enhancer activity of 32 HCC single cells (Left panel). The cells are ranked in the same order as Fig. 6a. Three super-enhancer-based cell subpopulations (SE-subpopulation) are identified. Right panels is the relative expression heat map of the gene sets related to mitochondrial activity, oncogenic signals pathways, tumor suppressor miRNAs, and oncomiRs of 32 HCC single cells

Changes in miRNA transcriptome can have a profound effect on the expression of several hundred mRNAs which propels the cells towards transformation [35, 36]. Although single-cell mRNA sequencing has provided many insights into the neoplastic progression and therapy of cancers [2, 37], the heterogeneity of miRNAs and its relationship with mRNA transcriptome in cancer still cannot be characterized at single-cell resolution. Here, we analyzed the small RNA-mRNA dual transcriptomes from 32 single cells isolated from a moderately differentiated human HCC sample (Additional file 1: Figure S18; Additional file 3: Table S13). Because the previous report has shown that miRNA and mRNA profiles possessed the better capability to assign single-cell heterogeneity [29], we used miRNAs and mRNAs to separate the 32 cells into three expression-based subpopulations (Exp-subpopulations) with six featured transcript groups (miRNAs and mRNAs) through an unsupervised hierarchical clustering analysis (Fig. 6a; Additional file 3: Table S14). Exp-subpopulation-I cells have a significantly higher expression of group 5 genes, significantly lower expression of group 2, 3, 4, and 6 genes and moderate expression of group 1 genes (*p* < 0.01; Fig. 6a; Additional file 1: Figure S19). Exp-subpopulation-II cells have a significantly higher expression of group 1, 2, and 6 genes, significantly lower expression of group 4 and 5 genes and moderate expression of group 3 genes (*p* < 0.05; Fig. 6a; Additional file 1: Figure S19). Exp-subpopulation-III cells have a significantly higher expression of group 1, 4, and 3 genes, significantly lower expression of group 5 genes and moderate expression of group 2 and 6 genes (*p* < 0.01; Fig. 6a; Additional file 1: Figure S19). The group 1 genes were not significantly enriched in Gene Ontology (GO) terms but contain F10, F9, PROZ, etc., which function in complement and coagulation cascades and have been considered biomarkers of many tumors [38, 39] (Fig. 6a, b; Additional file 3: Tables S14, S15). The group 3 genes were significantly enriched in the pathways of response to IFN-γ which are majorly contributed by HLA genes (Fig. 6a, b; Additional file 3: Tables S14, S15). The group 4 genes were significantly enriched in major signaling pathways promoting tumorigenesis, i.e., cell surface receptor signaling pathways [40, 41] (Fig. 6a, b; Additional file 3: Tables S14, S15). The group 5 genes were significantly enriched in GO terms which antagonize the Warburg effect of tumor cells, i.e., mitochondrial respiratory [42, 43] (Fig. 6a, b; Additional file 3: Tables S14, S15). Most interestingly, the groups 2 and 6 were miRNAs which contain 23 well-documented tumor suppressor miRNAs, such as miR26b, miR125, and miR139 [44–46] and two oncomiRs (miR221 and miR155) [47, 48] (Fig. 6a, b; Additional file 3: Table S14). Thus, Exp-subpopulation-I cells are likely less malignant (or benign) with high mitochondrial activity and low HLA gene expression [49] (Fig. 6a, b); the Exp-subpopulation-II cells are likely moderately malignant with low mitochondrial activity, moderately HLA gene expression and high tumor suppressor miRNA and oncomiR expression (Fig. 6a, b); the subpopulation-III cells are likely the most malignant with low mitochondrial activity, low tumor suppressor miRNA expression, high HLA gene expression, and high oncogenic signaling pathway activity (Fig. 6a, b). The significant downregulation of the group 2 and 6 miRNAs in Exp-subpopulation-III compared with Exp-subpopulation-II suggests that group 2 and 6 miRNAs contain potential tumor suppressors (Fig. 6a, b; Additional file 3: Table S14). Next, we pairwise compared the abundance of miRNAs with their potential targets in HCC single cells [31] (Additional file 3: Table S16) and revealed that 1765 miRNA-mRNA pairs displayed a negative correlation of their abundance (Additional file 3: Tables S17, S18). Among them, 265 potential miRNA-mRNA regulatory modules show that tumor suppressor miRNAs antagonize the expression of major oncogenes upregulated in Exp-subpopulation-III cells, such as PIK3CA, MYC, EGFR, and KRAS and oncomiRs antagonize the expression of tumor suppressors upregulated in Exp-subpopulation-I cells, such as CDKN1C, HES7, CDKN2A, and NFKB1 (Figs. 7a and 6a; Additional file 3: Tables S17, S18).

Super-enhancers (SEs) enable cell-type-specific gene regulation and the cell identity maintenance [26]. Furthermore, we used the abundance of master miRNAs and super-enhancer-regulated mRNAs to measure the genome-wide super-enhancer activity of these HCC cells [14] (Additional file 3: Tables S19, S20). The correlation matrix of genome-wide super-enhancer activity discriminated three super-enhancer-based subpopulations (SE-subpopulation-A, SE-subpopulation-B and SE-subpopulation-C) (Fig. 7b; Additional file 3: Table S21). All SE-subpopulation-C cells are from two malignant Exp-subpopulation-II and Exp-subpopulation-III (Fig. 7b). SE-subpopulation-B contains four cells (23, 5, 18, and 12) from Exp-subpopulation-I and two cells (31 and 17) from Exp-subpopulation-II (Fig. 7b). All SE-subpopulation-A cells are from less malignant (or benign) Exp-subpopulation-I (Fig. 7b). Interestingly, the super-enhancer activity of SE-subpopulation-B cells moderately correlates with that of both SE-subpopulation-A and SE-subpopulation-C cells, whereas the super-enhancer activity of SE-subpopulation-A and SE-subpopulation-C cells only correlates with that of the cells in same SE-subpopulations (Fig. 7b). These results suggest that SE-subpopulation-B cells are in benign-to-malignant transition and all malignant cells (moderately and most malignant cells) have similar genome-wide super-enhancer activity profile (Fig. 7b). Taken together, our small RNA-mRNA dual transcriptome single-cell sequencing suggests a potential hepatic neoplasm kinetics model showing that (1) HCC malignant transition couples with genome-wide super-enhancer remodeling and (2) the downregulation of mitochondrial activity and the upregulation of tumor suppressor miRNAs and oncomiRs happen in the early stage of malignant transition before activating tumorigenesis signaling pathways (Fig. 7b; Additional file 3: Table S22).

## Discussion and conclusions

Preamplification of long cDNA fragments, which have various lengths and secondary structures, is a key limitation to increasing the accuracy of current single-cell RNA-Seq methods. Unique molecular identifiers (UMIs) only work well for correcting moderate PCR bias. If a cDNA fragment is dramatically biased, its UMI can also be over- or under-represented at reasonable sequencing depths. Moreover, because UMIs can only tag cDNA fragment termini, the major reads inside a cDNA fragment cannot be used for improving quantification accuracy. Another drawback of preamplification is the loss of strand of origin information. Holo-Seq utilizes removable in vitro transcribed RNA as a carrier to protect against cellular RNA loss during single-cell lysis, reverse transcription and conventional library construction. Without the preamplification step, Holo-Seq successfully maintains the high accuracy, uniform coverage, and complete strand of origin information as conventional bulk RNA-Seq.

Among the diverse sources of total RNA-Seq mapped reads (rRNAs, intergenic regions, introns, and exons), rRNAs normally constitute 50–60% of mapped reads. Target sequencing could be an efficient add-on to focus on specific transcripts, such as mRNAs. To establish proof of principle, we successfully targeted the exon reads of highly expressed genes (48-fold enrichment) from a directional total RNA-Seq library of a single MCF7 cell using an Illumina TruSeq Exome Capture kit (FC-144-1004). If more library DNA is required for the target sequencing, we can use PCR primers that contain 8-nt degenerate barcodes to perform the first ten-cycle library amplifications followed by another fifteen-cycle amplification using regular P5/P7 primers. Because the length of library DNA fragments is short (200–500 bp), additional amplification only induces minimal bias, which can be effectively corrected in silico utilizing the degenerate barcodes (Additional file 1: Figure S20)*.*

A recent study developed an approach (RamDA-Seq) to amplify cDNA during reverse transcription [50]. Although RamDA can probe total RNA from a single cell with ideal coverage, it still cannot provide a complete strand of origin information that is crucial for non-coding RNA study [50]. Besides, Holo-Seq could be an open platform to adopt the amplification strategy of Smart-Seq, MATQ-Seq, SUePR-Seq, or RamDA-Seq when necessary.

Previous studies have demonstrated that small RNAs could effectively classify tumor tissues [51]. However, integrating miRNAs into the mRNA transcriptional regulatory network is still not ideal because the experiments are performed on bulk RNAs extracted from whole tissue; therefore, there is no guarantee that the observed small RNAs and mRNAs are in the same cellular space. Owing to the carrier RNA, Holo-Seq can perform poly-A selection to capture mRNAs before implementation of the conventional small RNA library pipeline, which enables simultaneously probing small RNAs and mRNAs in a single cell for the first time. Using Holo-Seq, we identified three Exp-subpopulations and three SE-subpopulations of HCC cells based on the miRNA-mRNA dual-transcriptomes and deciphered the hepatic neoplasm kinetics. Moreover, we filtered out a batch of tumor suppressor miRNA candidates with their potential targets and super-enhancers with significant activity change during malignant transformation. They are all potential biomarkers for the diagnosis or therapy of HCCs.

mRNA profile alone can also cluster the HCC cells into three similar groups with minimal variance (four cells from Exp-sub I and Exp-sub II rearranged) (Fig. 6a; Additional file 1: Figure S21 a, b). However, without miRNA information, the biological significance of the mRNA Subgroup II is difficult to explain (Additional file 1: Figure S21 a, b). miRNA profile alone can only cluster HCC cells into two groups (Additional file 1: Figure S21 c, d). The miRNA subgroup II cells are similar to the Exp-sub II cells based on combined expression profiles with minimal variance (two cells are from Exp-sub III) (Fig. 6a; Additional file 1: Figure S21 c,d). The miRNA subgroup I cells are the mixture of Exp-sub I and Exp-sub III based on combined expression profiles (Fig. 6a; Additional file 1: Figure S21 c,d). Without mRNA information, the biological significance of both miRNA subgroups is also difficult to explain (Additional file 1: Figure S21 c,d).

## Methods

### Cell culture

V6.5 (F1 hybrid of 129SvJae/C57BL/6) mESCs and ZHBTc4 cells were maintained and expanded on gelatin-coated (Millipore) plates in Dulbecco’s modified Eagle’s medium (DMEM; GIBCO) containing 15% FBS (GIBCO), 0.1 mM 2-mercaptoethanol (GIBCO), 2 mM GlutaMAX™-I (GIBCO), 0.1 mM MEM nonessential amino acids (NEAA; GIBCO), 1 mM sodium pyruvate (GIBCO), 1000 U/ml recombinant leukemia inhibitory factor (Millipore), and 30 U/ml penicillin/streptomycin (GIBCO). Approximately 200k cells were seeded in each 6-well plate (9.5 cm^2^/well; Corning). The media were changed daily, and mESCs were dissociated and expanded every 2 days.

MCF7 (human epithelial breast carcinoma) cells were cultured in DMEM (GIBCO) supplemented with 10% FBS (GIBCO) and 100 U/ml penicillin/streptomycin (GIBCO). Cells were seeded in each 6-well plate (9.5 cm^2^/well; Corning) and sub-cultured upon reaching 80–90% confluence.

HEK293T cells were cultured in DMEM (GIBCO) supplemented with 10% FBS (GIBCO) and 100 U/ml penicillin/streptomycin (GIBCO). Cells were seeded in the plate (Corning) and sub-cultured upon reaching 80–90% confluence.

### Preparation of carrier RNAs

Several DNA fragments with the T7 promoter were commercially synthesized to serve as the template. The HiScribe T7 High Yield RNA Synthesis kit (NEB) was used to perform in vitro transcription, and DNaseI was used to digest the template DNA before purifying the carrier RNA with a RNeasy Plus Mini kit (QIAGEN). The carrier RNA was aliquoted and stored at − 80 °C. The poly-A- and poly-A+ carrier RNAs were mixed (1:20) to mimic the ratio of mRNAs in a cell. We commercially synthesized a small carrier RNA (25 nt) and utilized T4 PNK to phosphorylate the 5′ end. The phosphorylated small carrier RNA mimic was purified with a miRNeasy Micro kit (QIAGEN) and stored at − 80 °C.

### Single-cell isolation

Cells were digested with 0.05% trypsin into a single-cell suspension, selected by mouth pipettes, and placed into 0.2-ml tubes containing 2 μl of 0.1% BSA/PBS. The selected cells were lysed immediately or stored at − 80 °C for later use.

### Hepatocellular carcinoma sample collection and single-cell isolation

Resected tumor sample was collected from a 46-year-old male patient and transported in DMEM (GIBCO) on ice immediately after surgical at The First Affiliated Hospital of School of Medicine, Zhejiang University. A small fragment was cut from the tumor and washed by PBS twice, then minced into pieces and transferred into a 50-ml tube (BD Falcon) containing 10 ml pre-warmed accumax (Stem Cell Technologies). Tumor pieces were digested for 30 min at 37 °C with continuous rotation to obtain single-cell suspension; this suspension was then filtered using a 70-μm strainer (Miltenyi Biotec) and supplemented with 30-ml PBS, centrifuge at 600*g* for 5 min at 4 °C. The supernatant was discarded, and the cell pellet was re-suspended in 300-μl PBS before staining for FACS. The single-cell suspension was stained with CD45-FITC (eBioscience) and 7-AAD (eBioscience) to deplete red blood cells, leucocytes, and non-viable cells. Cells with CD45 and 7-AAD negative staining were collected, and single cells were picked up with a mouth pipette into 200-μl tubes with 3-μl lysis buffer containing 0.2% Triton X100 (Sigma-Aldrich) and 4U recombinant RNase Inhibitor (TakaRa). The lysate was gently vortexed, fast centrifuged to the bottom of the tube, and incubated at room temperature for 5 min. Immediately stored at − 80 °C for later use.

### Sequencing library construction from bulk cells

Total RNA was extracted from approximately one million mESCs using an RNeasy Plus Mini kit (QIAGEN). The sequencing library was constructed following the manufacturer’s protocol using NEBNext kits (E7490, E7530, E7420, and E7300) for the mRNA library, the directional total RNA library, and the small RNA library, respectively.

### Sequencing library construction of mRNAs from a single cell

A single cell was lysed at 95 °C for 5 min in 0.1% BSA/PBS with 100 ng of the carrier RNA mixture. The sequencing library was constructed following the manufacturer’s protocol using NEBNext kits (E7490 & E7530). After adaptor ligation and USER digestion, cDNA fragments from the RNA carrier were removed by Not I digestion. After Not I digestion, the library DNA was purified for PCR amplification (16 cycles) (more details in “Step-by-step Holo-Seq protocols” (Additional file 4)).

### Directional sequencing library construction of total RNA from a single cell

A single cell was lysed at 95 °C for 5 min in 0.1% BSA/PBS with 100 ng of the carrier RNA mixture. The sequencing library was constructed following the manufacturer’s protocol using a NEBNext kit (E7420). After adaptor ligation, the cDNA fragments from the RNA carrier were removed by Not I digestion. After Not I digestion, the library DNA was purified for USER digestion and PCR amplification (16 cycles) (more details in “Step-by-step Holo-Seq protocols” (Additional file 4)).

### Sequencing library construction of total RNA from HEK293T single cells

A single cell was lysed at 95 °C for 5 min in 0.1% BSA/PBS with 100 ng of the carrier RNA mixture. The sequencing library was constructed following the manufacturer’s protocol using a NEBNext kit (E7530). After adaptor ligation and USER digestion, cDNA fragments from the RNA carrier were removed by Not I digestion. After Not I digestion, the library DNA was purified for PCR amplification (16 cycles).

### Sequencing library construction of small RNAs from a single cell and 1 ng mESCs total RNA

A single cell was lysed at 95 °C for 5 min in 0.1% BSA/PBS with 100 ng 5′-phosphorylated small carrier RNA mimic. Similarly, 100 ng 5′-phosphorylated small carrier RNA mimic was added to the diluted 1 ng mESCs total RNA. The small RNA sequencing library was constructed following the manufacturer’s protocol using a NEBNext kit (NEB #E7300). After PCR amplification (15 cycles), the cDNA fragment from the RNA carrier was removed by Not I digestion. After Not I digestion, the library DNA was purified for deep sequencing (more details in “Step-by-step Holo-Seq protocols” (Additional file 4)).

### Dual-sequencing library construction of small RNAs and poly(A) mRNAs from single cells

A single cell was lysed at 95 °C for 5 min in 0.1% BSA/PBS with 200 ng of the carrier RNA mixture (containing 100 ng 5′-phosphorylated small carrier RNA mimic). The poly-A RNAs were selected using a NEBNext Poly(A) mRNA Magnetic Isolation Module (NEB #E7490), and the small RNAs remained in the supernatant after poly-A selection. The small RNA sequencing library was constructed following the manufacturer’s protocol using a NEBNext kit (NEB #E7300). After PCR amplification (15 cycles), the cDNA fragment from the RNA carrier was removed by Not I digestion. After Not I digestion, the library DNA was purified for deep sequencing. The poly(A) mRNA sequencing library was constructed following the manufacturer’s protocol using a NEBNext kit (NEB #E7530).

### Smart-Seq2 library construction

cDNA from 1 ng of total mESC RNA or single cells was produced following the manufacturer’s protocol using the Smart-Seq2® v4 Ultra® Low Input RNA kit (Clontech) for single cells. Then, 2 ng of cDNA was sheared into 150- to 350-bp fragments by sonication, and a NEBNext DNA library preparation kit (E7645) was used to construct the sequencing library.

### Smart-Seq2 library construction coupled with Nextera XT workflow

cDNA from 1 ng mESC total RNA or single cells was produced following the manufacturer’s protocol using the Smart-Seq2® v4 Ultra® Low Input RNA kit (Clontech) for single cells. Then, 1 ng of cDNA was used to construct the sequencing library using Illumina Nextera XT DNA library preparation kit (FC-131-1024).

### Not I and Cas9 digestion of bulk RNA-Seq libraries

Total RNA was extracted from approximately one million mESCs or HEK293T using a miRNeasy Micro kit (QIAGEN). The libraries were constructed following the manufacturer’s protocol using NEBNext kits (E7490&E7530), before PCR enrichment, the DNA was digested by Not I at 37 °C for 2 h or Cas9 nuclease with sgRNA mix at 37 °C for 2 h.

### In vitro digestion of the DNA template of carrier RNA using CRISPR/Cas9

The sgRNA sites on the DNA template were selected using the Optimized CRISPR Design website. We used the Lenti-CRISPRv2 vector to express the sgRNAs in vitro. The in vitro transcribed sgRNAs were purified with the MEGAclear™ (Ambion AM1908). Approximately 300 nM of cDNA was digested by Cas9 nuclease (NEB M0641) with 30 nM sgRNA at 37 °C.

### Single-cell directional RNA-Seq library for captured sequencing

Construction of the single-cell directional RNA-Seq library was followed by directional sequencing library construction of total RNA from a single cell as described above from a single MCF-7 cell, PCR amplification was performed using primers containing 8-nt degenerate barcodes to perform the first 10 cycles of library amplification, followed by another 15 cycles of amplification by regular P5/P7 primers.

### First 10-cycle amplification primers

P5 primer with 8 nt degenerate barcodes:

5′-AATGATACGGCGACCACCGAGATCTACACNNNNNNNNACACTCTTTCCCTACACGACGCTCTTCCGATCT-3′

P7 primer with index:

5′-CAAGCAGAAGACGGCATACGAGATNNNNNNGTGACTGGAGTTCAGACGTGTGCTCTTCCGATCT-3′.

### Another 15-cycle amplification primers

Regular P5 primer: 5′-AATGATACGGCGACCACCGA-3′

Regular P7 primer: 5′-CAAGCAGAAGACGGCATACGAGAT-3′

### Mouse ES cells poly(A+) RNA-Seq (1 ng and bulk) data processing and gene expression analysis

Raw reads of 3 1 ng Holo-Seq libraries, 3 1 ng Smart-Seq2 libraries and 2 bulk mRNA-Seq libraries from mouse ES cells were mapped on mm9 genome by TopHat (v2.0.11) using the default settings.

Next, we sampled 6.8 million mapped reads from the Holo-Seq libraries, the Smart-Seq2 libraries, and the bulk mRNA-Seq libraries respectively. We counted the uniquely mapped reads in the exon regions of genes and calculated the RPKM values of the genes based on these reads.

At last, we selected genes with average RPKM larger than 0.1 in the 2 bulk mRNA-Seq libraries as expressed genes. We calculated Pearson correlation of their expression among Holo-Seq libraries, Smart-Seq2 libraries, and bulk mRNA-Seq libraries.

### mESC mRNA-Seq library hierarchical clustering analysis

1. Raw reads of 2 bulk mRNA-Seq libraries, 3 Holo-Seq(mRNA) libraries with 1 ng total RNA used, 3 Smart-Seq2 libraries with 1 ng total RNA used, 14 single-cell Holo-Seq(mRNA) libraries, and 7 single-cell Smart-Seq2 libraries are mapped to the mouse genome in the same way described above.

2. We select genes detected in both bulk libraries and calculate their RPKMs. Then, we use the maximum RPKM of each gene to divide its every RPKM among all these libraries to row-scale the expression of each gene.

3. We conduct hierarchical clustering on the 29 mRNA-Seq libraries by the scaled gene expression with heatmap.2 function of R: we measure similarity between the single cells by the Euclidean metric of scaled gene expression profiles of the bulk detected genes; we build binary tree with the averaged similarities among the libraries as linking parameter and we set other parameters of heatmap.2 with default.

### Mouse ES cells directional total RNA-Seq data processing and gene expression analysis

1. Raw reads of 10 single-cell Holo-Seq libraries and two bulk directional mRNA-Seq libraries from mouse ES cells were mapped to the mouse genome (mm9) by TopHat (v2.0.11) using the default settings. We sampled 0.64 million mapped reads from each single-cell Holo-Seq library, merged them into a combined library. We also sampled 6.4 million mapped reads from each of the two bulk libraries.
2. We counted uniquely mapped reads in the exon regions of genes in the two sampled bulk libraries and combined library. We calculated genes’ RPKM in the two sampled bulk libraries and combined library from these reads. Then, we got the average RPKMs of genes in the two sampled bulk libraries. We selected the genes with average RPKM larger than 0.1 as expressed genes. At last, we calculated the Pearson correlation of their average RPKMs in two sampled bulk libraries and their RPKMs in the combined library for the expressed genes.
3. We also counted uniquely mapped reads in the sense strand of the exons for each gene in the two sampled bulk libraries and combined library. We calculated genes’ strand-specific RPKM in the two sampled bulk libraries and combined library from these reads. Then, we got the average strand-specific RPKMs of genes in the two sampled bulk libraries. We selected the genes with average strand-specific RPKMs larger than 0.1 as expressed genes. At last, we calculated the Pearson correlation of their average strand-specific RPKMs in two sampled bulk libraries and their strand-specific RPKMs in the combined library for the expressed genes.

### Small RNA-Seq data processing and small RNA expression analysis for mouse ES single cells and human hepatocellular carcinoma single cells

We conducted processing and expression analysis of 10 mouse ES single-cell small RNA Holo-Seq libraries and the small RNA Holo-Seq libraries of 3 mouse ES single-cell small RNA-mRNA dual transcriptomes Holo-Seq sequencing with following steps:

1. We downloaded the reference sequences of mouse snoRNA, pre-miRNA, and tRNA from the Gencode M13, miRBase21, and GtRNAdb databases and built their bowtie2 indices.
2. We mapped the 50-bp raw reads on the reference sequences of snoRNA with bowtie2 (version 2.2.2). We used the “end-to-end” aligner to run bowtie2 with default parameters, except the mismatch parameter (-N) was set to 1. We counted reads mapped on snoRNAs as snoRNA reads counting.
3. We mapped the 50-bp raw reads on the mouse genome (mm9) by TopHat (v2.0.11) using the default settings and collected the unmapped reads.
4. We trimmed the unmapped reads to 30 bp (from 5′ to 3′) and mapped them on pre-miRNA using bowtie2. We used the “local” aligner to run bowtie2 with default parameters, except the mismatch parameter (-N) was set to 1 and the seed length parameter (−L) was set to 16. We selected reads with 17 to 25 continuous perfect matches (17 M to 25 M) in the mapped reads. We counted the selected reads mapped on the mature miRNA region of the pre-miRNA reference as miRNA reads counting.
5. We trimmed the unmapped reads of the third step to 40 bp (from 5′ to 3′) and mapped them on tRNA using bowtie2. We used the “local” aligner to run bowtie2 with default parameters, except the mismatch parameter (-N) was set to 1 and the seed length parameter (−L) was set to 30. We collected the mapped and unmapped reads from the first round of mapping. We then trimmed the unmapped reads from the first round of mapping to 30 bp (from 5′ to 3′) and mapped them on tRNA using bowtie2. We conducted the second round mapping in the same way as we did in the first round mapping, except the seed length parameter (-L) was set to 18. We counted the reads mapped on the tRNA from the first and second rounds of mapping as tsRNAs reads counting.
6. We calculated RPMs of snoRNA, miRNA, and tsRNA in the 13 libraries based on their reads counting.

For sequencing data of the 32 single-cell small RNA libraries of hepatocellular carcinoma dual transcriptome libraries, we downloaded the reference sequences of human snoRNA, pre-miRNA, and tsRNA from the Gencode GRCh37, miRBase21, and GtRNAdb databases respectively and built their bowtie2 indices. Then, we repeated the above 2–6 steps to calculate RPMs of snoRNA, miRNA, and tsRNA in the 32 human hepatocellular carcinoma single cells.

### Captured directional total RNA-Seq data processing and gene expression analysis

1. A single MCF7 cell directional Holo-Seq library of total RNA was used for target capturing using the Illumina TruSeq Exome Capture kit (FC-144-1004). After sequencing, raw reads of the captured library and the original Holo-Seq library were mapped to the human genome (hg19) by TopHat (v2.0.11) using the default settings.
2. We calculated barcoded RPKM of genes. The reads uniquely mapped to the coding regions were counted. We used the 8-nt degenerate barcode strategy to correct the PCR amplification bias. Identical mRNA molecules in single cells were expected to be less than 100 for most genes [10], and we found that unique positions (~ 99%) represented less than 10 reads in bulk mRNA-Seq (two million exome-mapped reads, ~ 12,000 expressed genes). Thus, after 10 amplification cycles with 8-nt degenerate barcode (65,536 barcodes) primers, one unique position generated no more than 10,240 different barcoded reads, and the barcode repeat chance was less than 0.01 with two million exome-mapped reads. We counted the reads with unique barcodes to represent the read counts on every mapped position and calculated the barcoded RPKM using the reads counts.
3. We calculated RPKM of genes. We counted the uniquely mapped reads in the captured library and the original Holo-Seq library from step 1 of the analysis and calculated RPKMs of genes in the two libraries.
4. We compared the RPKMs of genes in the captured library and the original Holo-Seq library. We selected genes whose RPKM was larger than 0.1 in either the captured library or the original Holo-Seq library. We calculated the Pearson correlation of their RPKMs for the selected genes. We compared their barcoded RPKMs of genes in the two libraries in the same way.

### Gene coverage analysis of mouse ES cells poly(A+) RNA-Seq data

1. Raw reads of 14 mouse ES single-cell Holo-Seq libraries and 7 mouse ES single-cell Smart-Seq2 libraries were mapped onto mm9 genome using TopHat (v2.0.11) with default parameters.
2. We merged all the exons of a gene together and then counted the number of mapped reads on every exon base pair. We summed all the exome-mapped reads to obtain the exon total read count. We divided the read counts of each base pair by the exon total read count to obtain the normalized coverage value of each base pair.
3. We divided the exons of each gene into 100 equal-sized bins. We summed the normalized coverage values of each base pair in each bin and then divided this number by the bin length to obtain the length-normalized coverage value of each bin. We excluded gene Cask, which had an abnormal huge number of reads mapped on its coding regions.
4. We grouped genes into 0–2 kb, 2–4 kb, 4–6 kb, 10–12 kb, 12 kp up, and 6 kb up groups according to their total exon length. We summed the length-normalized coverage values of each bin in a group to obtain the gene group read coverage values. We divided the length-normalized coverage values of each bin by the gene group read coverage value to obtain the value of group-normalized coverage values of each bin.
5. We calculated the bin coverage indicators (BCIs) of each bin in a group as follows: For the first BCI of the group, we summed the group-normalized coverage values of all the first bins of the genes in the group. After all 100 BCIs were calculated, we smoothed every BCI by:

## smoothed − BCI_(*n*)_ = average(BCI_*n* − 2_, BCI_*n* − 1_, BCI_*n*_, BCI_*n* + 1_, BCI_*n* + 2_).

6. We used the smoothed-BCI values to compare read coverage in different gene regions of different methods.

### Gene coverage analysis of HEK293T total RNA-Seq data

We downloaded HEK293T single-cell total RNA-Seq data of Smart-Seq2 (*n* = 5), MATQ-Seq (*n* = 5) and SUPeR-Seq (*n* = 5) [6, 22, 23]. We generated 6 HEK293T single-cell total RNA-Seq Holo-Seq data.

At first, we filtered out the reads mapped on human rRNA reference sequences (5S, 5.8S, 28S, 18S, and 45S pre-rRNA) by bowtie with default parameters.

Then, we conducted the steps 2–6 in the section “Gene coverage analysis of mouse ES cells poly(A+) RNA-Seq data” to perform gene coverage analysis of HEK293T single-cell total RNA-Seq data. However, we removed six genes (RMRP, RPPH1 from Holo-Seq data; CASC5, MATAL1, PDCD4 from Smart-Seq2 data; HIST1H4C from MATQ-Seq data) during the gene coverage analysis because they had an abnormal huge number of reads mapped on their coding regions.

### De novo identification of antisense transcription from mouse ES cells directional total RNA-Seq data

1. We combined the raw reads of 10 mouse ES single-cell directional total RNA Holo-Seq libraries and removed the redundant reads.
2. We mapped the reads onto the mm9 genome using TopHat (v2.0.11).
3. We distinguished the reads mapped on the + and − strand of the genome according to the strand information.
4. We called read islands using spatial clustering for identification of ChIP-enriched regions (SICER) (Version 1.1). We conducted the first round of the read island calling with a big window parameter (1000 bp) and a gap parameter (3000 bp). Then, we conducted the 2nd round of the read island calling with a small window parameter (200 bp) and a gap parameter (600 bp). All other SICER parameters were set as the default.
5. We filtered out the read islands that were 1000 bp in the first round of island calling and contained one read island smaller than 401 bp in the second round of island calling.
6. We downloaded the gene annotation profiles from the UCSC, Ensemble, and RefSeq databases (mm9 version). We identified read islands as unannotated antisense transcripts if they were covered by no known genes on the same strand and 20% of their length was overlapped by any annotated genes on the opposite strand.

### t-SNE analysis of mouse ES cells by their gene expression profiles from poly(A+) RNA-Seq data

Raw reads of 2 bulk RNA-Seq libraries, 3 1 ng Holo-Seq libraries, 14 single-cell Holo-Seq libraries, 3 1 ng Smart-Seq2 libraries, and 7 single-cell Smart-Seq2 libraries from mouse ES cells were mapped to the mm9 genome by TopHat (v2.0.11) using the default settings. We calculated RPKM of genes in the libraries.

We conducted t-SNE analysis on these 29 libraries by their gene expression profiles. First, genes expressed in both bulk RNA-Seq libraries (RPKM > 0.1) were picked as expressed genes. Then, we conducted PCA analysis on their RPKM profiles in 29 libraries for the expressed genes with the *prcomp* function in R. At last, the top principle components contributed to 85% of the sum of eigenvalues were picked as input for t-SNE analysis using the R software package *Rtsne* (perplexity = 6).

### t-SNE analysis of mouse ES cells and mouse T cells by their antisense transcripts expression profiles from directional total RNA-Seq data

1. Raw reads of 10 mouse ES single-cell directional RNA Holo-Seq libraries, two mouse ES cell bulk directional RNA Holo-Seq libraries, and two mouse T cell bulk directional RNA libraries [18] were mapped to the mouse genome (mm9) by TopHat (v2.0.11) using the default settings.
2. We got the unannotated antisense transcripts from the analysis of the section “De novo identification of antisense transcription from mouse ES cells directional total RNA-Seq data” and got known antisense transcripts. We filtered out the antisense transcripts (either unannotated antisense transcripts or known antisense transcripts) if more than 80% of their length is covered by the known genes on the opposite strand.
3. We counted the uniquely mapped reads in the sense strand of the antisense transcripts in the libraries. We calculated RPKM based on these reads as their strand-specific RPKM for the antisense transcripts. We selected the antisense transcripts who had at least 10 reads mapped in at least one library and of whom strand-specific RPKM is larger than 0.1 in at least one library.
4. We conducted t-SNE analysis on all 14 libraries by the strand-specific RPKMs of antisense transcripts in the libraries. We employed *prcomp* function in R to conduct PCA analysis. Then, the top principle components contributed to 85% of the sum of eigenvalues were picked as input for t-SNE analysis using the R software package *Rtsne* (perplexity = 3).

### Hierarchical clustering analysis of mouse ES single cells by their gene expression profiles and antisense transcript expression profiles from directional total RNA-Seq data

1. Raw reads of 10 mouse ES single-cell directional total RNA Holo-Seq libraries were mapped to the mouse genome (mm9) by TopHat (v2.0.11) using the default settings. We counted the uniquely mapped reads in the sense strand of the exon regions (RefSeq gene annotation profile, mm9 version) and calculated the RPKM of genes based on the reads as their strand-specific RPKM.
2. We repeated the step 2 in the section “t-SNE analysis of mouse ES cells and mouse T cells by their antisense transcripts expression profiles from directional total RNA-Seq data” to get antisense transcripts. We counted the uniquely mapped reads in the sense strand of the antisense transcripts and calculated RPKM based on the reads as their strand-specific RPKMs.
3. We selected the expressed gene or antisense transcript if at least 10 reads were mapped on it in at least one library and its strand-specific RPKM is larger than 0.1 in at least one library. We conducted hierarchical clustering on the 10 libraries by the strand-specific RPKMs of expressed genes with heatmap.2 function of R. We set the distance parameter as “euclidean” and the agglomeration method as “complete”. We also conducted hierarchical clustering on the 10 libraries by the strand-specific RPKM of expressed antisense transcripts in the same way.

### Hierarchical clustering analysis of mouse ES single cells from small RNA-Seq data

1. We got RPMs of snoRNA, miRNA, and tsRNA for 13 mouse ES single cells from the step 1 of the section “Small RNA-Seq data processing and small RNA expression analysis for mouse ES single cells and human hepatocellular carcinoma single cells”. We selected the snoRNA, miRNA, and tsRNA with at least 10 mapped reads in at least one single cell as expressed snoRNA, miRNA, and tsRNA.
2. We conducted hierarchical clustering on the 13 single cells by the PRMs of expressed miRNAs with heatmap.2 function of R. We set the distance parameter as “euclidean” and the agglomeration method as “complete”. We conducted hierarchical clustering on the 13 single cells by the PRMs profiles of expressed tsRNA or snoRNA in the same way.

### Hierarchical clustering analysis of hepatocellular carcinoma single cells from small RNA-mRNA dual transcriptomes Holo-Seq data

1. Raw reads of 32 hepatocellular carcinoma single-cell small RNA and mRNA dual Holo-Seq libraries were mapped on human genome hg19 by TopHat (v2.0.11) using the default settings. We calculated RPKMs of genes. We analyzed the section “Small RNA-Seq data processing and small RNA expression analysis for mouse ES single cells and human hepatocellular carcinoma single cells” on the paired 32 small RNA Holo-Seq library to get RPMs of miRNA. We added 1 in the RPKMs values of genes and in the RPMs values of miRNAs. We then log2-transformed the values.
2. We calculated the mean (*u*) and standard deviation (*s*) of the transformed expression values across 32 cells for each mRNA and miRNA. Next, we excluded the mRNAs and miRNAs with very low average expression (*u* < 1) and used *z*-scoring methods of a previous study [52] to measure the variation (*V*) of each mRNA and miRNA which is calculated as *V* = *u*/*s*^2^. At last, we ranked mRNA according to their *V* value in increasing order and selected top 500 mRNA. In the same way we selected top 99 miRNA. We combined the top 500 mRNA, top 99 miRNA, and miR-26a-5p as featured mRNAs and miRNAs.
3. We scaled the log2-transformed expression value of featured mRNA and miRNA in 32 cells with *Z*-score transform. We set the distance parameter as “euclidean” and the agglomeration method as “complete” and used the heatmap.2 function of R to cluster the cells and featured miRNAs and mRNAs.

### Calculate Fano factor of introns and exons of a gene in mouse ES single cells from directional total RNA-Seq data

1. We mapped the raw reads of 10 mouse ES single-cell directional total RNA Holo-Seq libraries on the mouse genome (mm9) by TopHat (v2.0.11) using the default settings.
2. We counted the uniquely mapped reads in the sense strand of the exons of genes (RefSeq gene annotation profile, mm9 version) and calculated the RPKMs as genes’ mRNA RPKMs. We counted the uniquely mapped reads in the sense strand of the introns of genes and calculated the RPKM as their intron RPKMs. We selected the expressed genes meeting three requirements: (a) their mRNA RPKMs and intron RPKMs are both larger than 0.1 in at least 5 cells; (b) the number of uniquely mapped reads is larger than 5 in the sense strand of their exon regions in at least 5 cells and the number of uniquely mapped reads is larger than 1 in the sense strand of their intron regions in at least 5 cells; and (c) their intron length is larger than 1000 bp.
3. We calculated the mean and variance of the mRNA RPKMs of each expressed gene across 10 single cells. We also calculated the mean and variance of the intron RPKMs of each expressed gene. We calculated the exon RPKM Fano factor of an expressed gene as the ratio of variance to mean of its mRNA RPKMs. We calculated the intron RPKM Fano factor of the expressed gene as the ratio of variance to mean of its intron RPKMs.

### Infer miRNA and target gene regulatory network in mouse ES cells from small RNA-mRNA dual transcriptomes Holo-Seq data

1. Seven mouse ES single-cell small RNA-mRNA dual transcriptomes Holo-Seq libraries were sequenced. We mapped the reads of mRNA Holo-Seq libraries on mm9 by TopHat (v2.0.11) using the default settings. We calculated RPKMs of genes. We analyzed the section “Small RNA-Seq data processing and small RNA expression analysis for mouse ES single cells and human hepatocellular carcinoma single cells” on the paired 7 small RNA Holo-Seq libraries to get RPMs of miRNAs.
2. We downloaded 40,670 experimentally validated mouse miRNA-target gene pairs from miRTarBase (Release 7.0) and selected 1306 miRNA-target pairs validated by different experimental approaches. Then, we selected the detected miRNAs (with at least 1 mapped reads in at least two single cells) and got 623 miRNA-target pairs.
3. Next, we used the Spearman coefficient to measure the correlation between miRNAs and target genes of the filtered miRNA-target pairs among the 7 single cells. Interactions whose miRNA and target gene expression is of significantly negative correlation (*p* < 0.05 and Spearman coefficient < 0) were selected to miRNA and target gene regulatory network.

### Infer miRNA and target gene regulatory network in hepatocellular carcinoma single cells from small RNA-mRNA dual transcriptomes Holo-Seq data

1. We calculated the RPKMs of genes and the RPMs of miRNAs in 32 hepatocellular carcinoma single cells with the step 1 of the section “Hierarchical clustering analysis of hepatocellular carcinoma single cells from small RNA-mRNA dual transcriptomes Holo-Seq data”.
2. We downloaded 380,633 experimentally validated human miRNA-mRNA pairs from miRTarBase (Release 7.0).
3. We inferred miRNA and target gene regulatory network for oncomiR miR-155-5p and miR-221-5p in hepatocellular carcinoma. At first, we selected the miRNA-target interactions of miR-155-5p and miR-221-5p. Then, we filtered out the interactions in which either the miRNA or the target gene was detected in less than 1/3 cells of all 32 single cells, which means the RPM of the miRNA or the RPKM of the gene is zero in at least 22 cells. Next, we used the Spearman coefficient to measure the correlation between miRNAs and target genes of the filtered interactions in 32 single cells. Interactions whose miRNA and target genes’ expression is of significantly negative correlation (*p* < 0.05 and Spearman coefficient < 0) were selected. At last, we conducted differential gene expression analysis on the miRNAs and target genes of the selected interactions. We compared expression of the miRNAs and target genes between Exp-subpopulation I and Exp-subpopulation II & III by *t* test with unequal variance and single-tailed distribution. The interactions whose miRNA and target gene are both significantly differentially expressed (*p* < 0.05) were kept. We got 345 interactions between miR-155-5p and miR-221-5p and 342 target genes to build miRNA and target gene regulatory network of miR-155-5p and miR-221-5p. The target genes are characterized with tumor suppressive identity referring to the KEGG and GeneCards database were annotated in miRNA and target gene regulatory network for oncomiR miR-155-5p and miR-221-5p (Additional file 3: Table S17).
4. We inferred miRNA and target gene regulatory network for potential tumor suppressor miRNAs in Exp-subpopulation II and Exp-subpopulation III of hepatocellular carcinoma. At first, we got miRNA-target interactions from step 2 of the analysis and filtered out the interactions of known oncomiRs (here they are miR-155-5p and miR-221-5p). Then, we excluded interaction whose miRNA or target gene was detected in less than 1/3 cells of the 14 cells from Exp-subpopulation II and III, which means the RPMs of the miRNA or the RPKMs of the gene is zero in at least 10 cells. Next, per each filtered interaction, we used the Spearman coefficient to measure the expression correlation of its miRNA and target gene in the 14 cells. We selected 1420 interactions whose miRNA and target genes’ expression is in significant negative correlation (*p* < 0.05 and Spearman coefficient < 0) to build miRNA and target gene regulatory network for potential tumor suppressor miRNAs. The target genes are characterized with oncogenic identity referring to the KEGG and GeneCards databases were annotated in miRNA and target gene regulatory network for potential tumor suppressor miRNAs (Additional file 3: Table S18).

### Identify super-enhancer (SE)-related master miRNAs and mRNAs in mouse ES cells from small RNA-mRNA dual transcriptomes Holo-Seq data

1. We got RPKMs of genes and RPMs of miRNAs from step 1 of the section “Infer miRNA and target gene regulatory network in mouse ES cells from small RNA-mRNA dual transcriptomes Holo-Seq data”. We kept the expressed miRNAs which had at least 10 mapped reads in at least one single cell and their RPM > 0.1 in at least one single cell. We kept the expressed genes which had at least 10 mapped reads in at least one single cell and their RPKM > 0.1 in at least one single cell.
2. We downloaded 233 established super-enhancers (SEs) in mouse ES cell from a previous study [32]. We associated the 233 SEs with their regulating miRNAs and genes. We found the closest gene and miRNA (or miRNA cluster) in ± 100 kb of each SE (RefSeq gene annotation profile, mm9) as SE associated miRNA and gene. We corrected our association pairs using previous studies [14, 32].
3. We got 169 SE-expressed gene pairs and 15 SE-expressed miRNA pairs.

### Infer cell subpopulations of hepatocellular carcinoma single cells by expression of super-enhancer (SE)-related genes and miRNAs from small RNA-mRNA dual transcriptomes Holo-Seq data

1. We calculated the RPKMs of mRNAs and the RPMs of miRNAs in 32 hepatocellular carcinoma single cells with the step 1 of the section “Hierarchical clustering analysis of hepatocellular carcinoma single cells from small RNA-mRNA dual transcriptomes Holo-Seq data.”
2. We downloaded 497 established super-enhancers (SEs) in human HepG2 cell line identified by a previous study [33]. Then, we followed the steps 2 of the section “Identify super-enhancer (SE)-related master miRNAs and mRNAs in mouse ES cells from small RNA-mRNA dual transcriptomes Holo-Seq data” to get 213 SE-expressed gene pairs and 14 SE-expressed miRNA pairs.
3. We ranked the 32 single cells following their order in Exp-subpopulations I, II, and III. We calculated the Spearman coefficient of SE-related genes and miRNAs between the 32 single cells.
4. We selected the oncogenic signal gene group, mitochondrial function gene group, tumor suppressive miRNA group, and oncomiR group (Additional file 3: Table S22). We calculated the expression value (*Group* _ *Exp*) of the four gene groups in the 32 cell with the step 1 of the section “Differential expression analysis of the gene groups 1–6 between cell subpopulation I, II, and III (Exp-subpopulations I, II, and III) of hepatocellular carcinoma”. For a gene group in the single cell *i*, we calculated the gene group’s activity (*Group* _ *Act*_*i*_) with following formula:

$$ \mathrm{Group}\_{\mathrm{Act}}_i=\frac{\mathrm{Group}\_{\mathrm{Exp}}_i-\mathrm{Group}\_{\mathrm{Exp}}_{\mathrm{min}}}{\mathrm{Group}\_{\mathrm{Exp}}_{\mathrm{max}}-\mathrm{Group}\_{\mathrm{Exp}}_{\mathrm{min}}}, $$

where Group _ Exp_*i*_ is the expression value of the gene group in the single cell *i*; Group _ Exp_min_ is the minimum value of the gene group expression across 32 single cells; Group _ Exp_max_ is the maximum value of the gene group expression in 32 single cells. We calculated their activities of the four gene groups in the 32 cells.

### GO term analysis of gene groups identified from “Hierarchical clustering analysis of hepatocellular carcinoma single cells from small RNA-mRNA dual transcriptomes Holo-Seq data”

We conducted GO term analysis on the gene (mRNA and miRNA) groups with DAVID functional annotation tools (version 8.0). The background gene set was set as all the genes in the human genome. We annotated the gene groups on the terms of the biological process of GO with annotation level 5 (GO_BP_5 in David). We selected the terms with Benjamini corrected *P* value < 0.05 as significant GO terms.

### Hierarchical clustering analysis of top variant mRNAs and miRNAs

1. We select top 1000 variant mRNAs as featured mRNAs following the step 1 and step 2 from the method of the section “Hierarchical clustering analysis of hepatocellular carcinoma single cells from small RNA-mRNA dual transcriptomes Holo-Seq data.”
2. We scale the log2-transformed expression value of featured mRNAs with *Z*-score transform. We set the distance parameter as “euclidean” and the agglomeration method as “complete” and used the heatmap.2 function of R to cluster the 32 HCC single cells and featured mRNAs.
3. We select top 500 variant mRNAs, top 190 variant miRNAs, and top 99 variant miRNAs & mir-26a-5p, then respectively cluster the 32 HCC single cells and featured mRNAs/miRNAs as we do with the top 1000 variant mRNAs.

### Differential expression analysis of the gene groups 1–6 between cell subpopulation I, II, and III (Exp-subpopulations I, II, and III) of hepatocellular carcinoma from small RNA-mRNA dual transcriptomes Holo-Seq data

1. We got the log-transformed expression values of genes and miRNAs in 32 hepatocellular carcinoma single cells with the step 1 of the section “Hierarchical clustering analysis of hepatocellular carcinoma single cells from small RNA-mRNA dual transcriptomes Holo-Seq data.” We extracted their expression values in 32 cells for the genes (mRNA and miRNA) in the gene groups 1–6. Next, per each gene group, we scaled the expression value of its genes across the 32 cells with *Z*-score transform and then calculated the median value of the scaled values in each cell as the expression value of the gene group (Group _ Exp) in the cell. We calculated their Group _ Exp for gene groups 1–6 in 32 cells.
2. We compared the Group _ Exp of gene groups 1–6 between different cell subpopulations (Exp-subpopulations I, II, and III). We used *t* test with equal variance and two-tailed distribution to conduct the differential expression analysis.

## Additional files

Additional file 1: **Figure S1.** Carrier RNAs sequences and gel images of in vitro digestions. **Figure S2.** Holo-mRNA-Seq flowchart. **Figure S3.** RPKM scatterplots of expressed genes after Not I and Cas9 digestion. **Figure S4.** RPKM scatterplots of expressed genes. **Figure S5.** Hierarchical clustering of mRNA transcriptomes. **Figure S6.** Hierarchical clustering heatmap of mESC bulk-mRNA-Seq, mESC Holo-Seq (1 ng total RNA and single-cell total RNA), and mESC Smart-Seq2 (1 ng total RNA and single-cell total RNA). **Figure S7.** Comparison of Holo-mRNA-Seq with Smart-Seq2 coupled with Nextera XT workflow. **Figure S8.** The saturation curves of Holo-Seq, Smart-Seq2, SUPeR-Seq and MATQ-Seq. **Figure S9.** Holo-Seq flowchart for total RNA with a complete strand of origin information. **Figure S10.** Hierarchical clustering of expressed genes and antisense transcripts. **Figure S11.** Signal plot of the Rpe locus by Holo-Seq. **Figure S12.** Comparison of the diversity of antisense transcripts and coding transcripts at similar expression level. **Figure S13.** RPKMs of mRNAs and introns of selected core genes and housekeeping genes. **Figure S14.** Holo-Seq flowchart for profiling small RNAs. **Figure S15.** The saturation curves of miRNA. **Figure S16.** RPM scatterplots of expressed small RNAs. **Figure S17.** Relative expression heat maps of super-enhancer-regulated master miRNAs and mRNAs. **Figure S18.** Hematoxylin and Eosin (HE) staining of the HCC tissue. **Figure S19.** Relative expression levels of gene groups between HCC Exp-subpopulations. **Figure S20.** mRNA capture sequencing of the Holo-Seq total RNA library. **Figure S21.** mRNA and miRNA solo transcriptome analyses of hepatocellular carcinoma (HCC) single cells. (DOCX 5908 kb)

Additional file 2: **Table S1.** Not1-site-containing transcripts in mouse. **Table S2.** Not1-site-containing transcripts in human. **Table S3.** Sequencing statistics of RNA libraries. **Table S4.** Single cell library cost with different methods. (XLSX 171 kb)

Additional file 3: **Table S5.** Known and novel antisense transcripts identified from 10 mESC single cells. **Table S6.** Core and housekeeping genes displayed in Fig. 3e. **Table S7.** miRNAs detected in 13 mESC single cells. **Table S8** snoRNAs detected in 13 mESC single cells. **Table S9.** tsRNAs detected in 13 mESC single cells. **Table S10.** List of miRNAs and their potential target genes detected in 7 mESC single cells. **Table S11.** Super-enhancers and their regulated master miRNA(expressed) in 7 mESC single cells. **Table S12.** Super-enhancers and their regulated mRNAs (expressed) in 7 mESC single cells. **Table S13.** miRNAs detected in 32 HCC single cells. **Table S14.** Six featured transcript groups in Fig. 6a. **Table S15.** GO term analysis of transcripts of groups 1, 3, 4, 5 in Fig. 6a. **Table S16.** List of miRNAs and their potential target genes detected in 32 HCC single cells. **Table S17.** List of oncomiRs (miR-155-5p, miR-221-5p) and their target gene pairs. **Table S18.** miRNAs and their target gene pairs expressed in negative correlation (*p* < 0.05) among Exp-sub II&III. **Table S19.** Super-enhancers and their regulated master genes expressed in 32 HCC single cells. **Table S20.** Super-enhancers and their regulated master miRNAs expressed in 32 HCC single cells. **Table S21.** The matrix of super-enhancer regulated master miRNAs and genes’ expression correlation (Spearman r) among 32 HCC single cells. **Table S22.** List of gene sets in the right panel in Fig. 7b. (XLSX 2860 kb)

Additional file 4: Step-by-step Holo-Seq protocols. (DOCX 29 kb)

Additional file 5: Review history. (DOCX 53 kb)

## Abbreviations

bp: Base pair
cDNA: Complementary DNA
CRISPR: Clustered regularly interspaced short palindromic repeats
Exp-subpopulations: Expression-based subpopulations
GO: Gene ontology
HCA: Hierarchical cluster analysis
HCC: Human hepatocellular carcinoma
HEK293T: Human embryonic kidney cells 293
Holo-Seq: Holo-transcriptome sequencing
kb: Kilobase
MAD: Median absolute deviation
mESC: Mouse embryonic stem cell
miRNA: Micro RNA
mRNA: Messenger RNA
nt: Nucleotide
PCA: Principle component analysis
PCR: Polymerase chain reaction
pre-mRNA: Precursor mRNA
RPKM: Reads per kilobase per million mapped reads
RPM: Reads per million mapped reads
rRNA: Ribosomal RNA
SC: Single cell
SD: Standard deviation
SE: Super enhancer
SE-subpopulations: Super-enhancer-based subpopulations
snoRNA: Small nucleolar RNA
t-SNE: T-distributed stochastic neighbor embedding
tsRNA: tRNA-derived small RNA
UMI: Unique molecular identifier
