# Supplementary material for: Holo-Seq: single-cell sequencing of holo-transcriptome
Source: Genome Biol. 2018 Oct 17;19:163. doi: 10.1186/s13059-018-1553-7 (PMC6193298; doi:10.1186/s13059-018-1553-7)
Supplement: Supplementary file 1 — Figure S1. Carrier RNAs sequences and gel images of in vitro digestions. Figure S2. Holo-mRNA-Seq flowchart. Figure S3. RPKM scatterplots of expressed genes after Not I and Cas9 digestion. Figure S4. RPKM scatterplots of expressed genes. Figure S5. Hierarchical clustering of mRNA transcriptomes. Figure S6. Hierarchical clustering heatmap of mESC bulk-mRNA-Seq, mESC Holo-Seq (1 ng total RNA and single-cell total RNA), and mESC Smart-Seq2 (1 ng total RNA and single-cell total RNA). Figure S7. Comparison of Holo-mRNA-Seq with Smart-Seq2 coupled with Nextera XT workflow. Figure S8. The saturation curves of Holo-Seq, Smart-Seq2, SUPeR-Seq and MATQ-Seq. Figure S9. Holo-Seq flowchart for total RNA with a complete strand of origin information. Figure S10. Hierarchical clustering of expressed genes and antisense transcripts. Figure S11. Signal plot of the Rpe locus by Holo-Seq. Figure S12. Comparison of the diversity of antisense transcripts and coding transcripts at similar expression level. Figure S13. RPKMs of mRNAs and introns of selected core genes and housekeeping genes. Figure S14. Holo-Seq flowchart for profiling small RNAs. Figure S15. The saturation curves of miRNA. Figure S16. RPM scatterplots of expressed small RNAs. Figure S17. Relative expression heat maps of super-enhancer-regulated master miRNAs and mRNAs. Figure S18. Hematoxylin and Eosin (HE) staining of the HCC tissue. Figure S19. Relative expression levels of gene groups between HCC Exp-subpopulations. Figure S20. mRNA capture sequencing of the Holo-Seq total RNA library. Figure S21. mRNA and miRNA solo transcriptome analyses of hepatocellular carcinoma (HCC) single cells. (DOCX 5908 kb) [file 13059_2018_1553_MOESM1_ESM.docx]

**
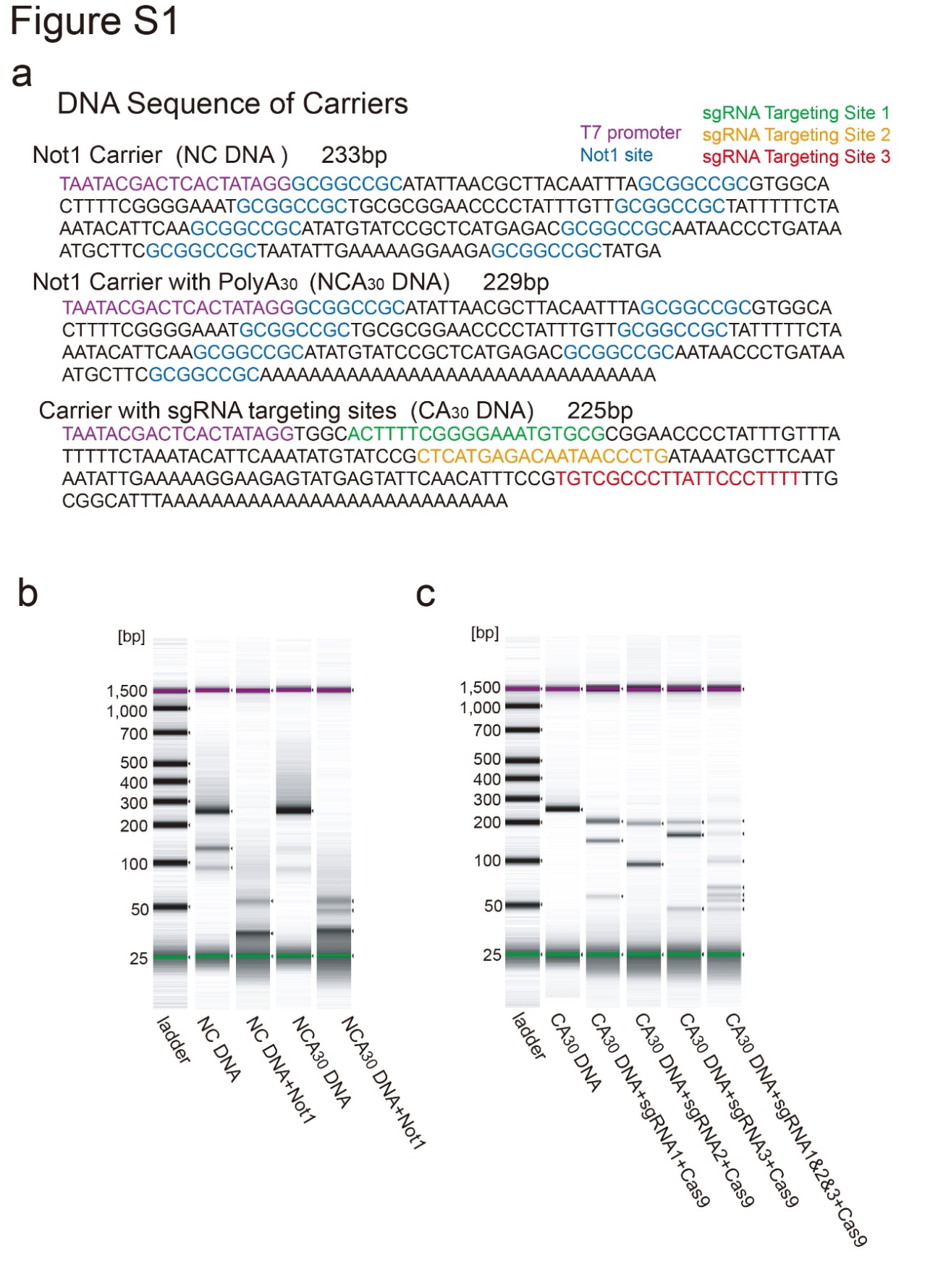
**

**Figure S1: Carrier RNAs sequences and gel images of in vitro digestions.**

a. The sequences of DNA templates used to produce carrier RNAs.

b. In vitro digestion of carrier RNA templates by NotI.

c. In vitro digestion of carrier RNA templates by CRISPR/Cas9.


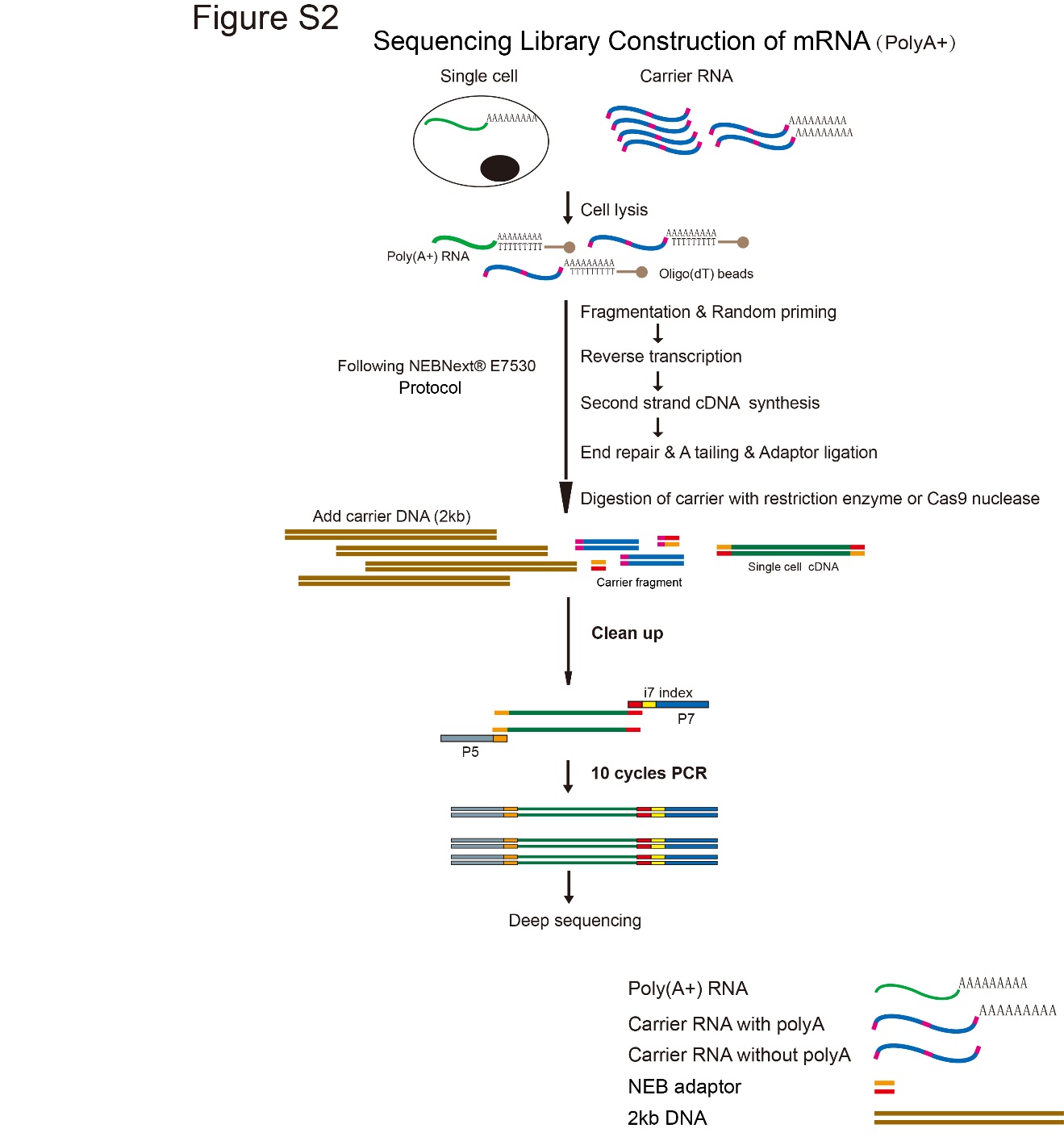


**Figure S2**: **Holo-mRNA-Seq flowchart.**

A single cell was lysed with carrier RNA mixture. The sequencing libraries were constructed following the manufacturer’s protocol using an NEB kit (E7530) after poly-A selection. After adaptor ligation and USER digestion, cDNA fragments from the RNA carrier were removed by NotI digestion. Then, the library DNA was purified for PCR amplification and deep sequencing.


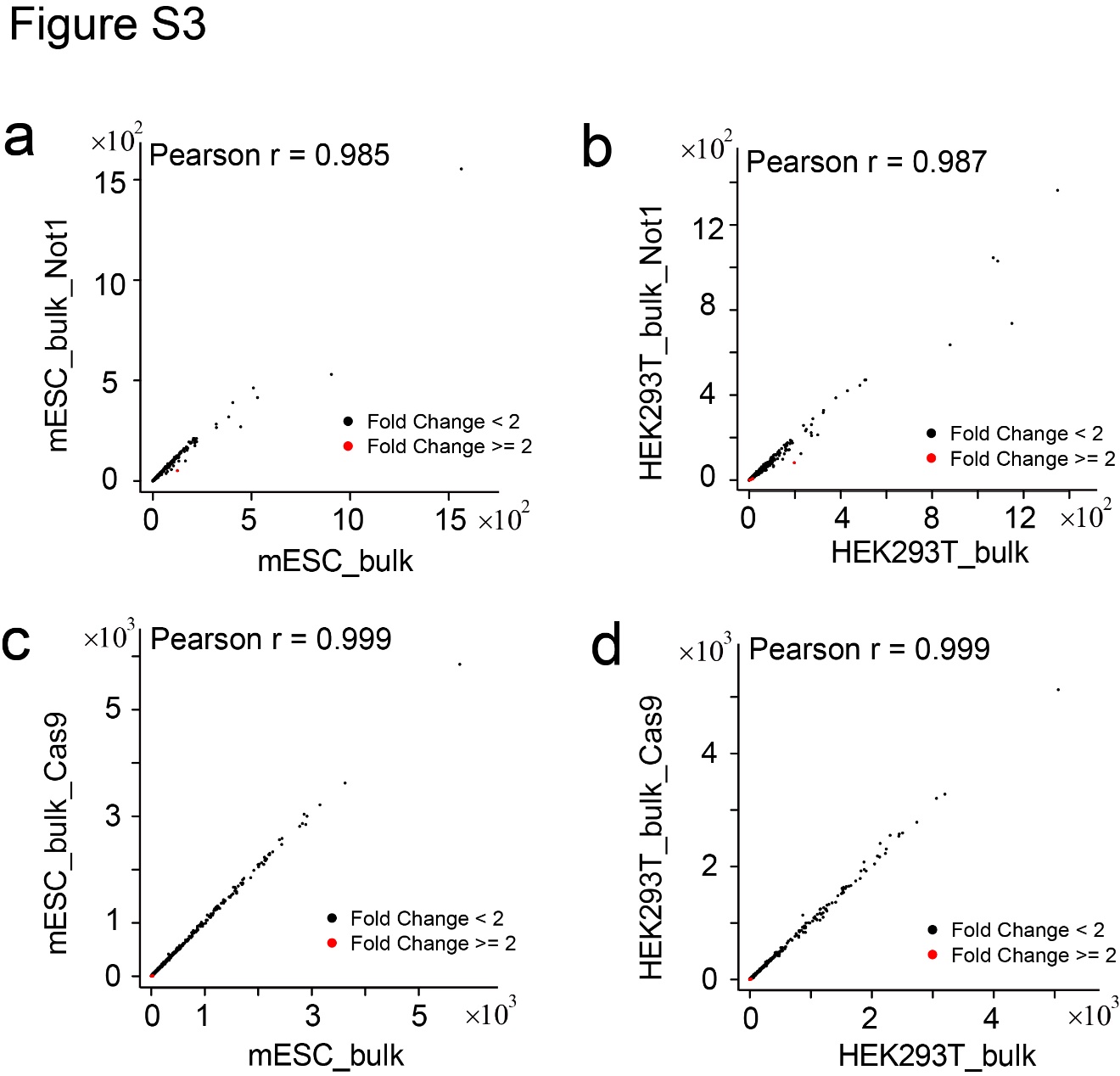


**Figure S3: RPKM scatterplots of expressed genes after Not I and Cas9 disgestion.**

a-b, RPKM scatterplots of Not I-site-containing transcripts between Not I digested bulk mRNA-Seq libraries and normal bulk mRNA-Seq libraries. (a, mESC; b, HEK293T)

c-d, RPKM scatterplots of expressed genes between Cas9 digested bulk mRNA-Seq library and normal bulk mRNA-Seq library. (c, mESCs; d, HEK293T)

**
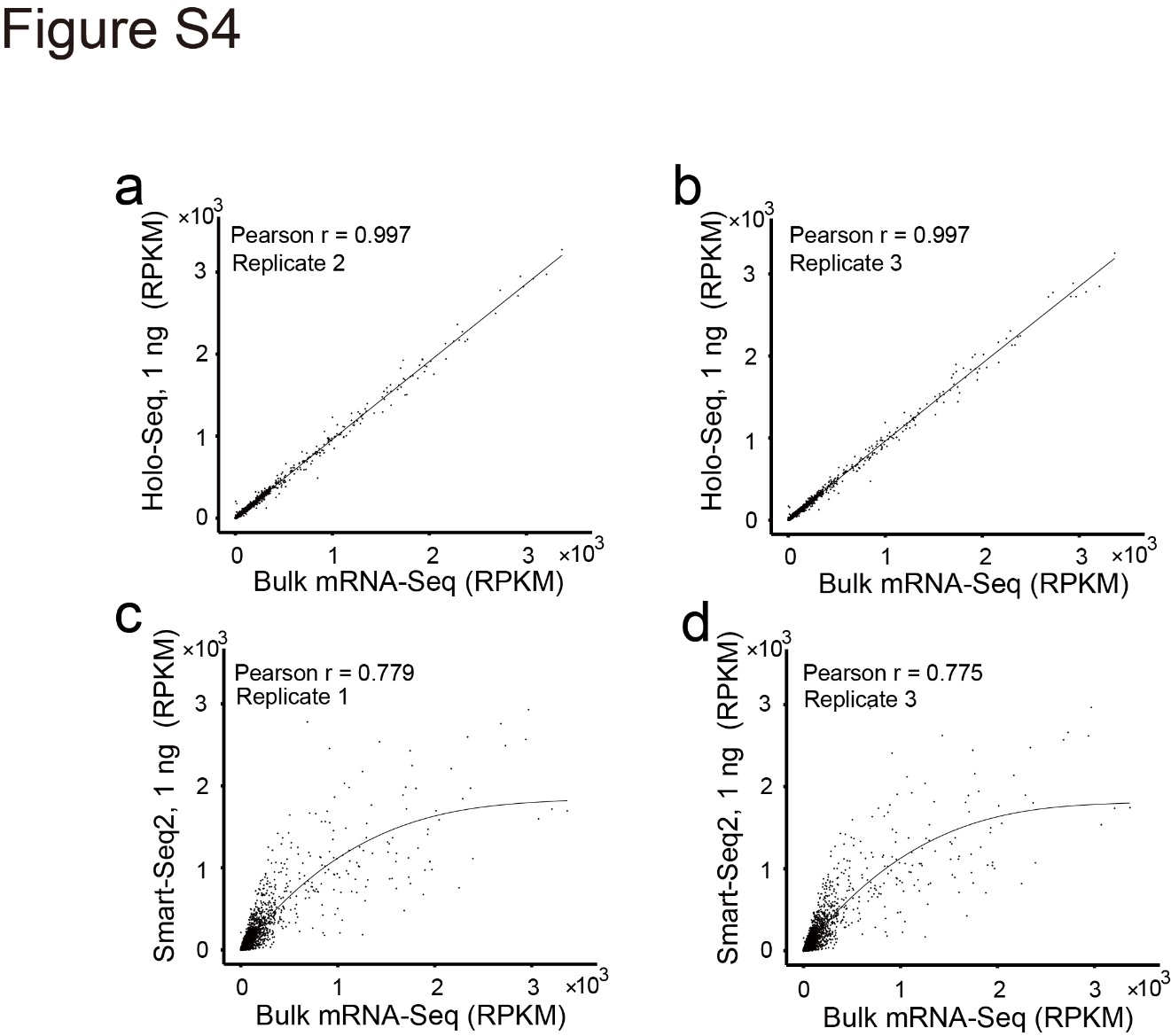
**

**Figure S4: RPKM scatterplots of expressed genes.**

a-b, RPKM scatterplot of expressed genes between Holo-mRNA-Seq (1 ng mESC total RNA) and bulk mRNA-Seq.

c-d, RPKM scatterplot of expressed genes between Smart-Seq2 (1 ng mESC total RNA) and bulk mRNA-Seq.

**
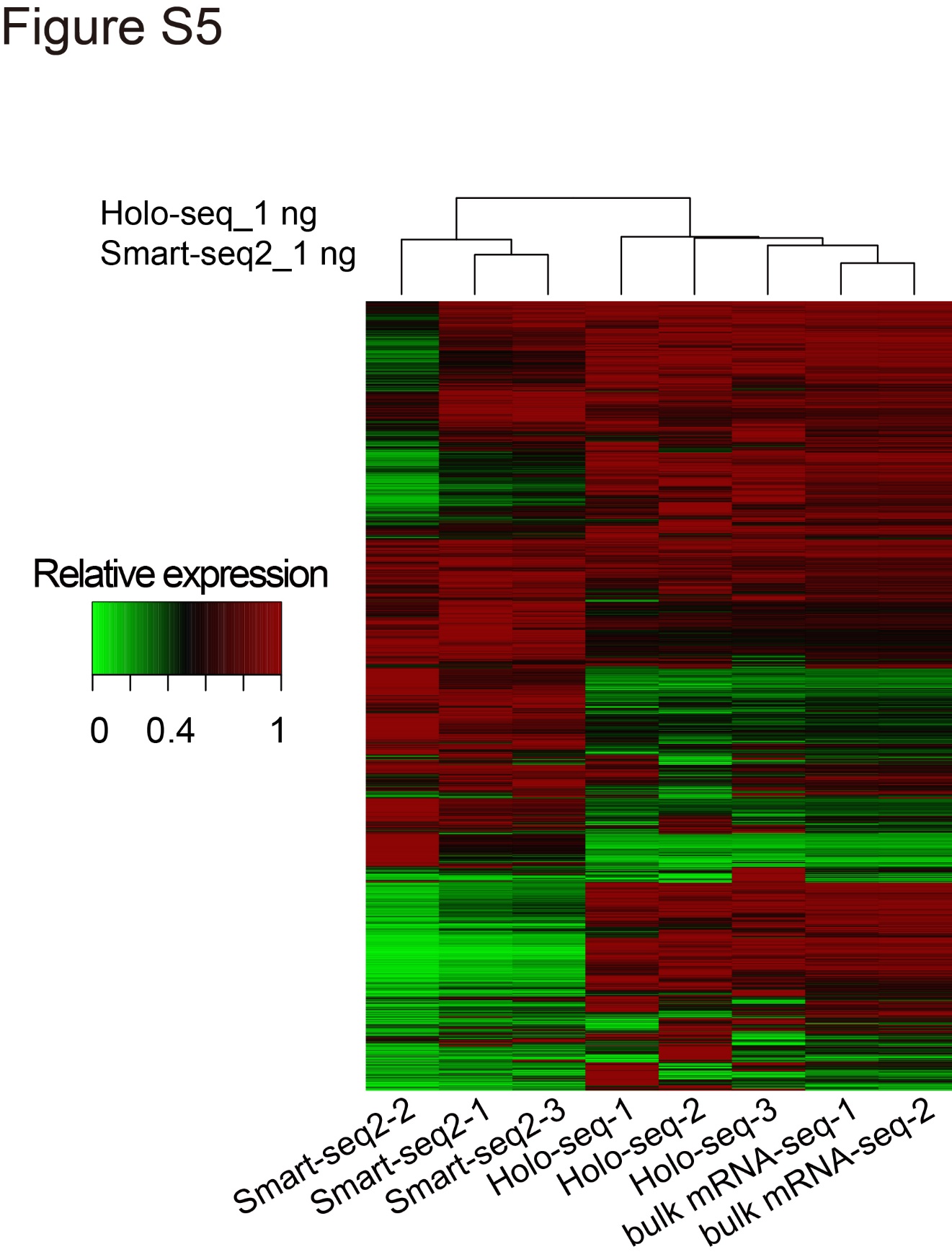
**

**Figure S5: Hierarchical clustering of mRNA transcriptomes.**

Hierarchical clustering of mRNA transcriptomes detected by Smart-Seq2 (1 ng of mESC total RNA), Holo-mRNA-Seq (1 ng of mESC total RNA) and bulk mRNA-Seq.

**
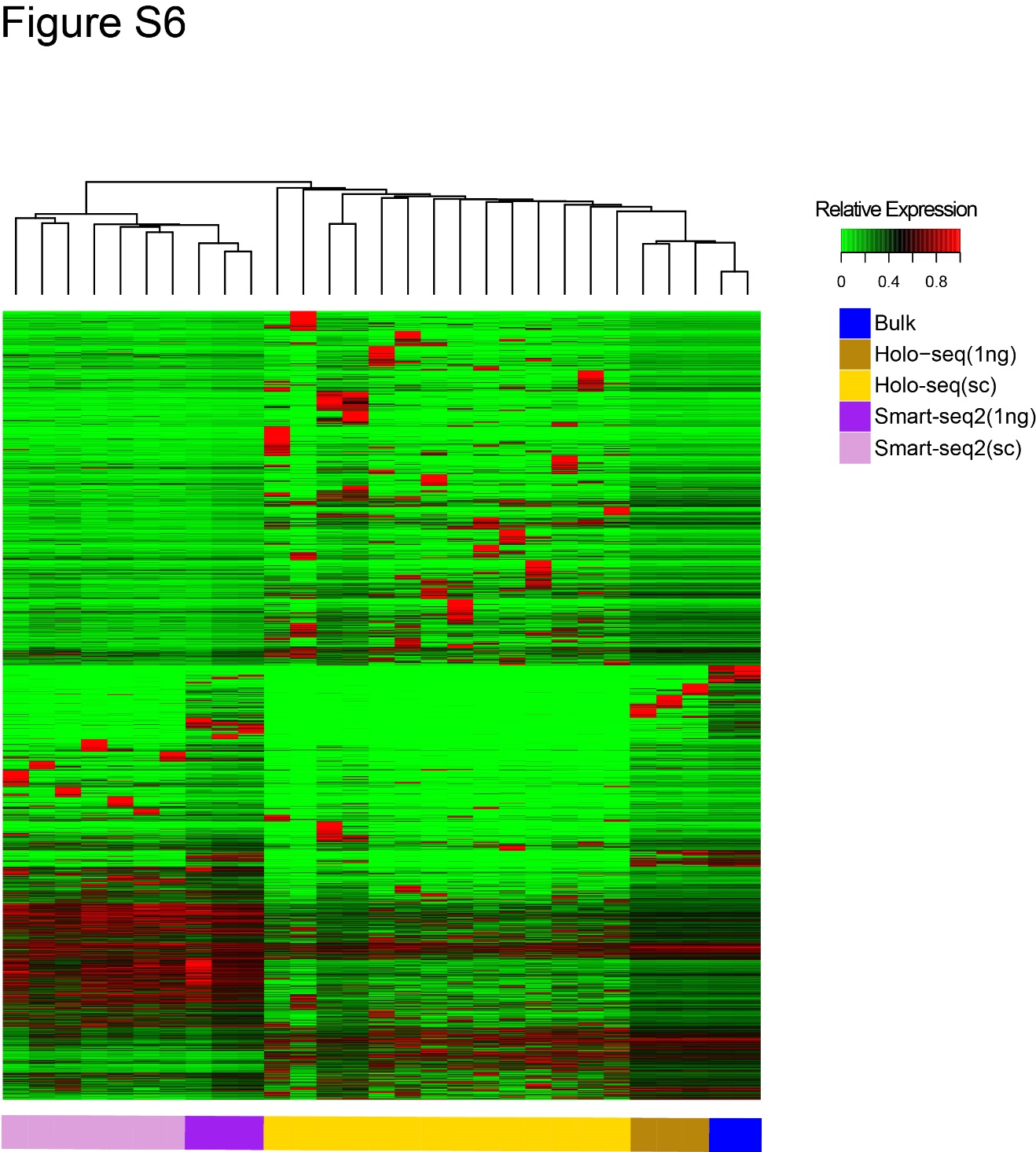
**

**Figure S6:** Hierarchical clustering heatmap of mESC bulk-mRNA-Seq, mESC Holo-Seq (1ng total RNA and single-cell total RNA) and mESC Smart-Seq2 (1ng total RNA and single-cell total RNA).


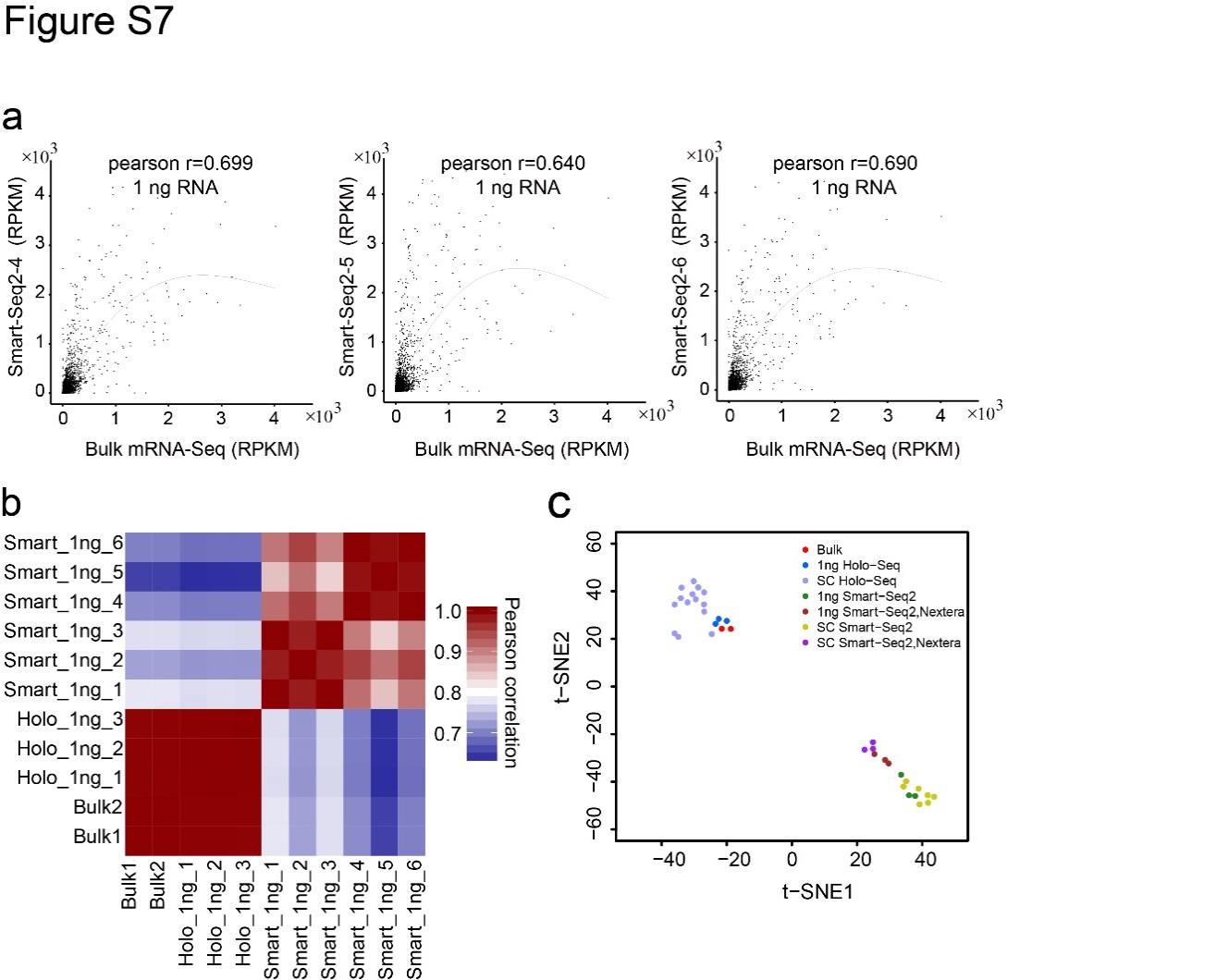


**Figure S7: Comparison of Holo-mRNA-Seq with Smart-Seq2 coupled with Nextera XT workflow**

a. RPKM scatterplots of expressed genes between Smart-Seq2 and bulk mRNA-Seq. 1 ng of mESC total RNA was used and the full-length cDNA output of the Smart-Seq2 were processed with the Nextera XT DNA library preparation kit. Three biological (Smart-Seq2-4,5,6) replicates were performed.

b. Pearson correlation coefficient heat-map of the mRNA profiles generated from 1 ng of total RNA by Smart-Seq2 (with NEB library preparation kit), Smart-Seq2 (with Nextera XT library preparation kit) and bulk-mRNA-Seq. Three biological replicates were performed.

c. t-SNE analysis of mESCs (bulk-mRNA-Seq), mESC single cells (Holo-Seq and Smart-Seq2) and 1ng mESCs total RNA (Holo-Seq and Smart-Seq2). Principal components were used as inputs. Both NEB library preparation kit and Nextera XT DNA library preparation kit were used to generate Smart-Seq2 single-cell and 1ng libraries.

**
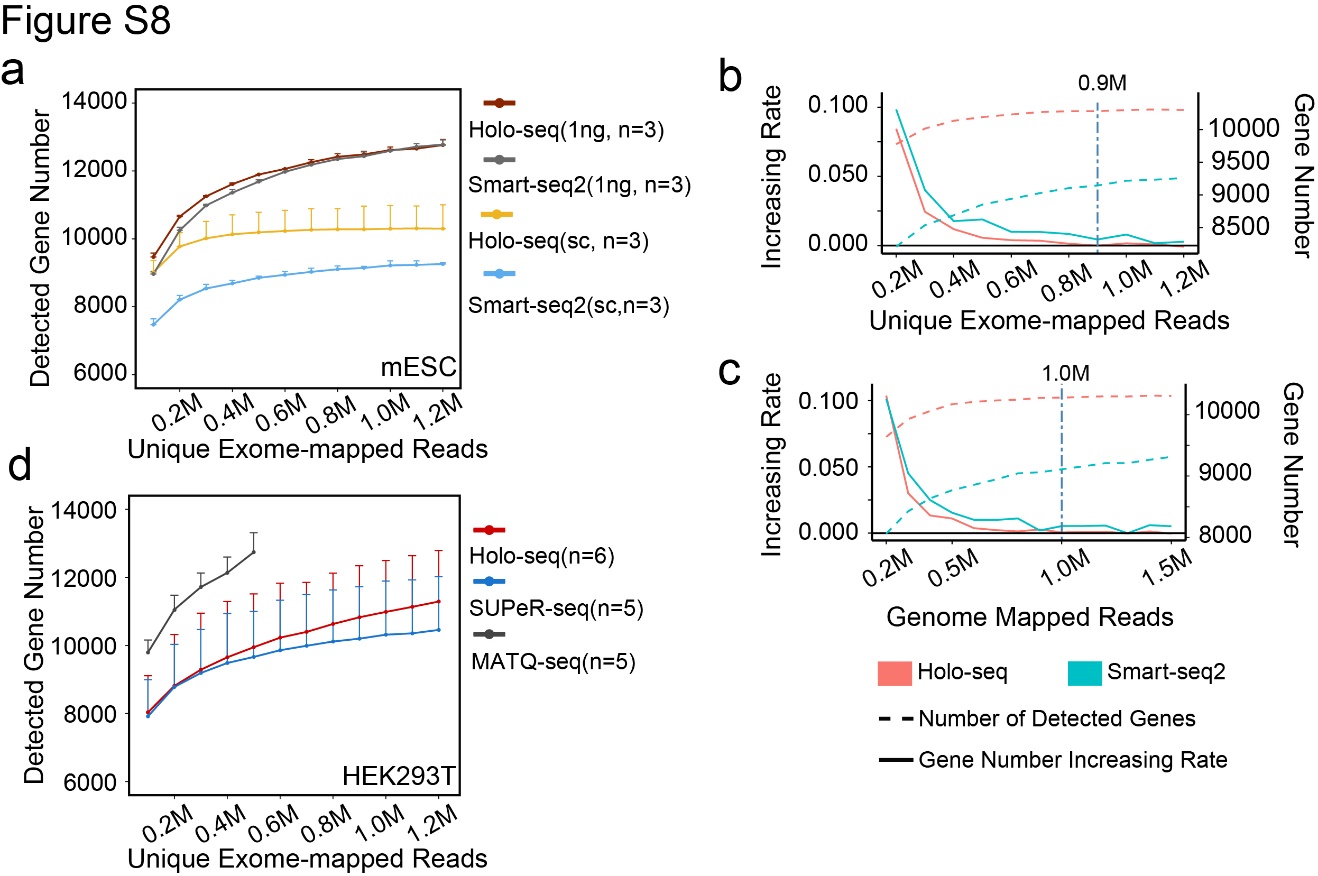
**

**Figure S8: The saturation curves of Holo-Seq, Smart-Seq2, SUPeR-Seq and MATQ-Seq.**

a. The number of detected genes at different unique exome-mapped depth of Holo-Seq and Smart-Seq2 libraries (1ng mESCs total RNA and mESC single cells; Poly-A mRNAs), b-c. Comparison of increasing rate of detected gene number at different unique exome-mapped depth (b) and genome mapped depth (c) of above libraries. d. the number of detected genes at different unique exome-mapped depth of Holo-Seq, SUPeR-Seq and MATQ-Seq single cell libraries (HEK293T single cells; UMI labeled MATQ reads were used). Error bars: Median Absolute Deviation (MAD).

**
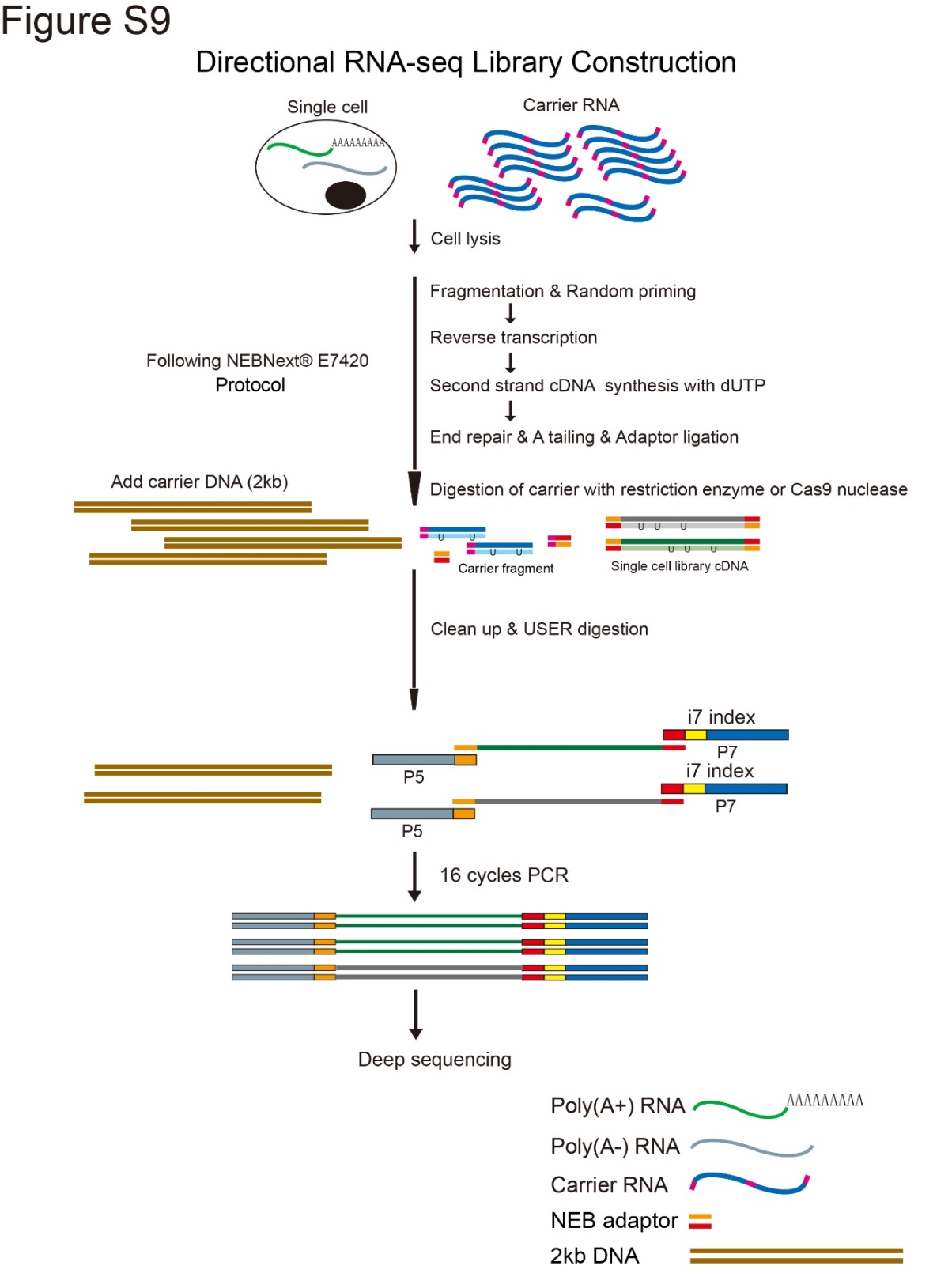
**

**Figure S9: Holo-Seq flowchart for total RNA with a complete strand of origin information.**

A single cell was lysed with the carrier RNA mixture. The sequencing libraries were constructed following the manufacturer’s protocol using a NEBNext kit (E7420). After adaptor ligation, the cDNA fragments from the RNA carrier were removed by NotI digestion. After NotI digestion, the library DNA was purified for USER digestion and then subjected to PCR amplification and deep sequencing.

**
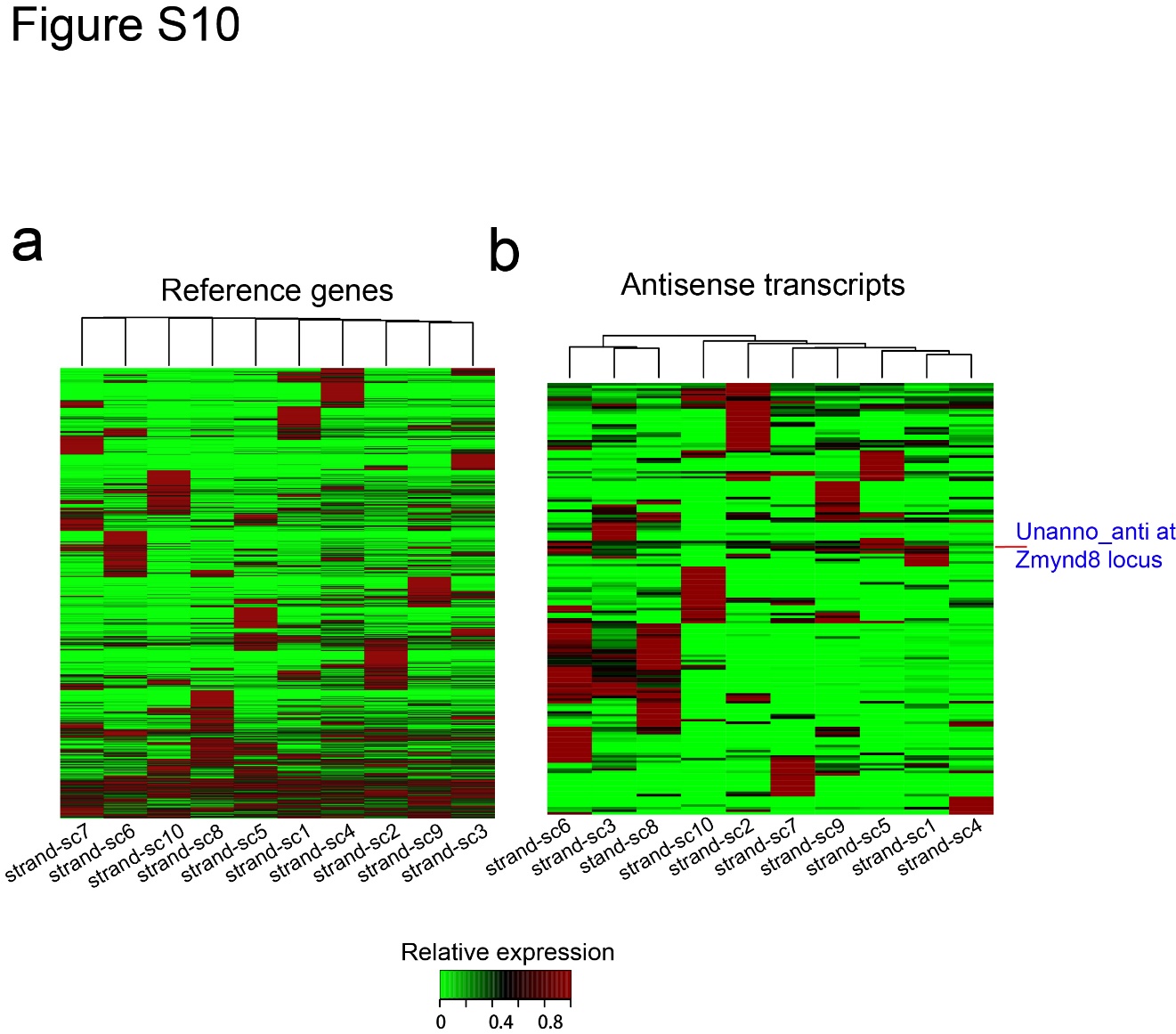
**

**Figure S10: Hierarchical clustering of expressed genes and antisense transcripts.**

Hierarchical clustering of the expressed gene (a) and antisense transcript (b) profiles from 10 mESC single cells by Holo-Seq directional total RNA pipeline.

**
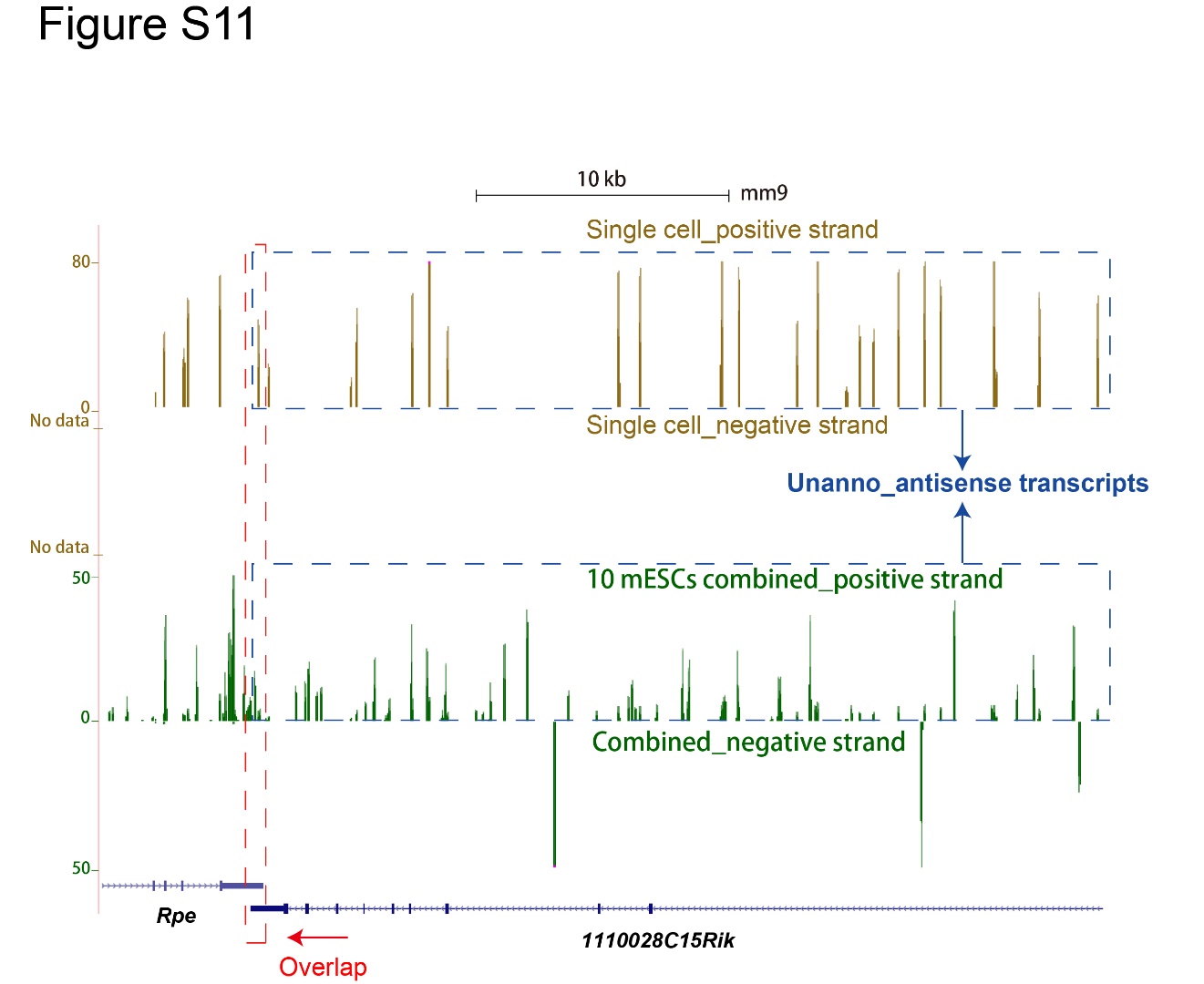
**

**Figure S11: Signal plot of the Rpe locus by Holo-Seq.**

Signal plot of the Rpe locus (positive strand) from mESC single cell directional total RNA sequencing datasets. The arrow indicates that the transcriptional signal could be erroneously attributed to the overlapped gene (1110028C15Rik, negative strand) without a strand of origin information.

**
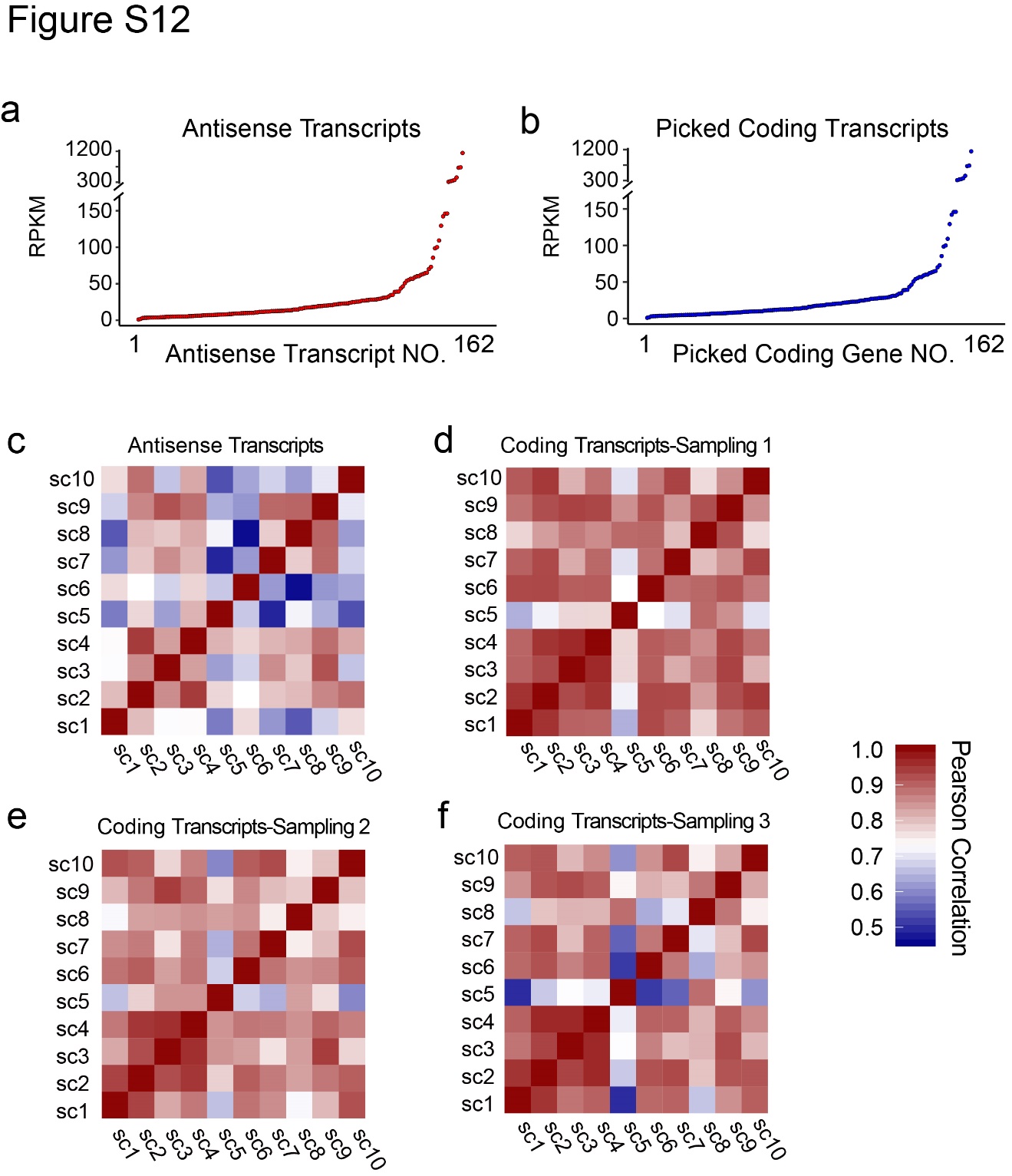
**

**Figure S12: Comparison of the diversity of antisense transcripts and coding transcripts at similar expression level**

a-b. Scatterplots of the expression level (the highest RPKM among 10 mESC single-cell libraries) of 162 expressed antisense transcripts (a), and the 162 randomly selected coding transcripts which have a similar expression distribution as the antisense transcripts (b).

c-f. Pearson correlation heatmaps between single cells of the 162 antisense transcripts (c) and 162 randomly selected coding transcripts which have a similar expression distribution as the antisense transcripts (d-f). Three rounds of random picking were performed.

**
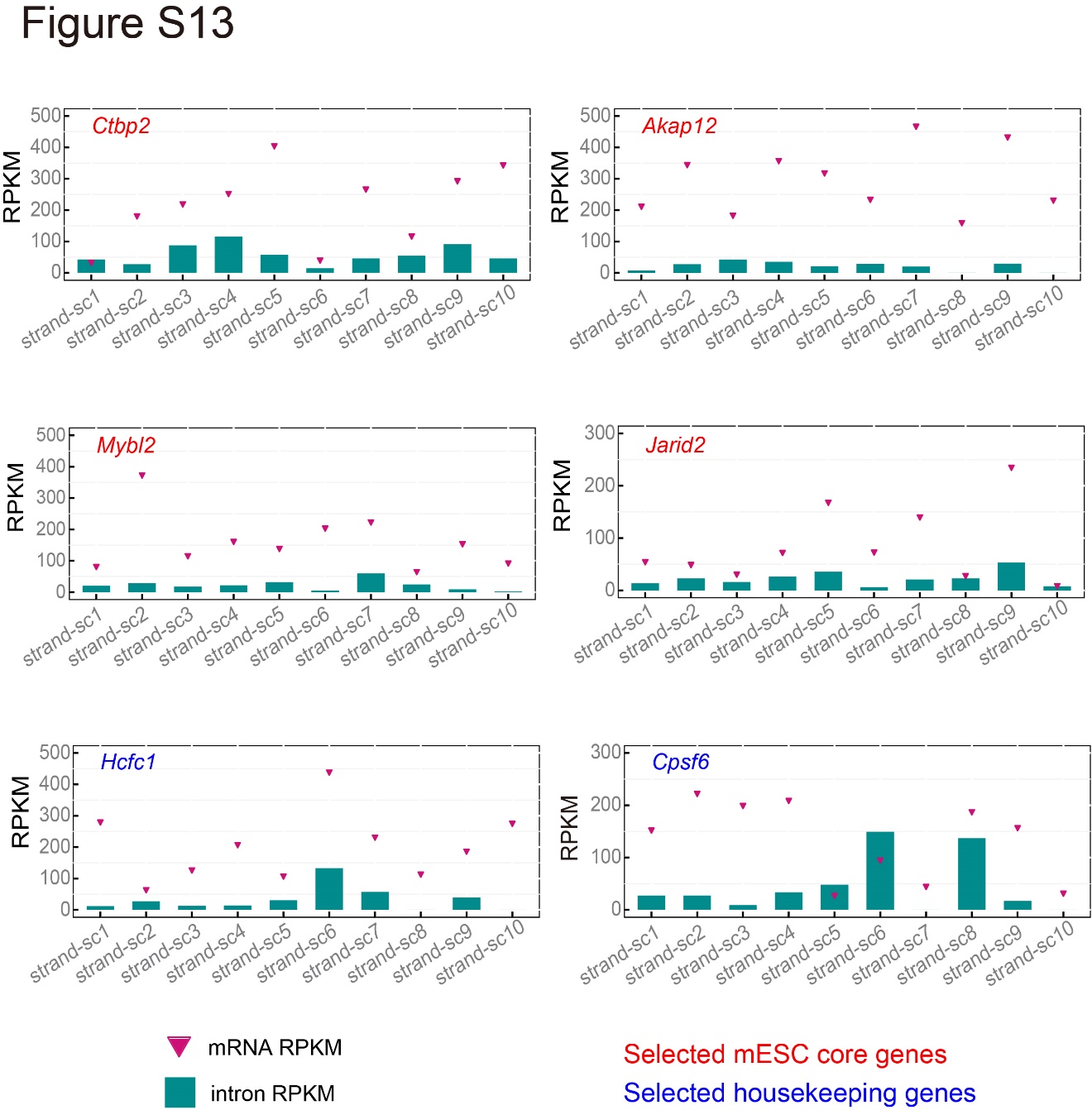
**

**Figure S13: RPKMs of mRNAs and introns of selected core genes and housekeeping genes.**

The mRNA RPKM and the intron RPKM of core genes (*Ctbp2, Akap12, Mybl12* and *Jarid2*) and housekeeping genes (*Hcfc1* and *Cpsf6*) in 10 mESC single cells.

**
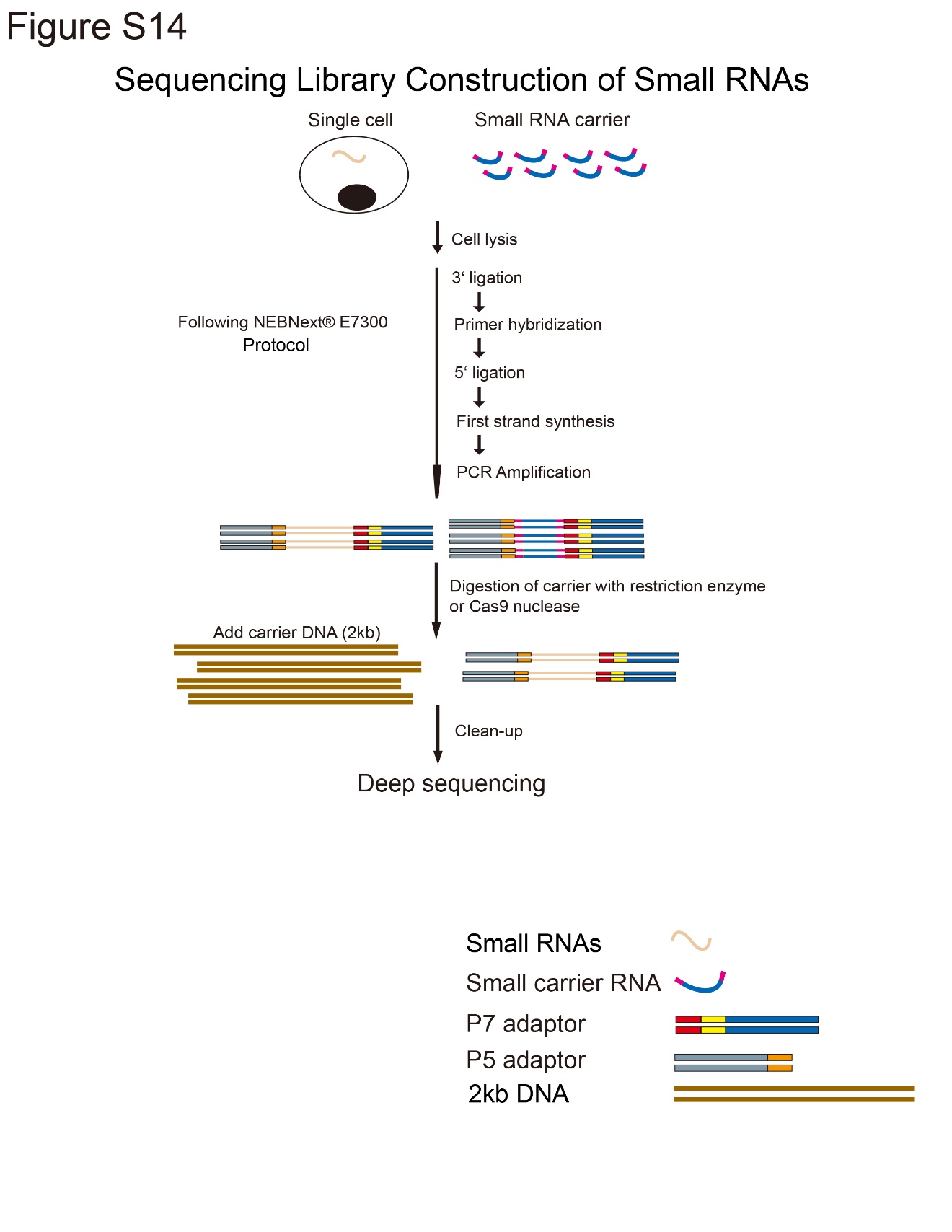
**

**Figure S14: Holo-Seq flowchart for profiling small RNAs.**

A single cell was lysed with the carrier RNA mixture. Small RNA sequencing libraries were constructed following the manufacturer’s protocol using a NEBNext kit (E7300). After PCR amplification, cDNA fragments from the RNA carrier were removed by NotI digestion. After NotI digestion, the library DNAs were purified for deep sequencing.


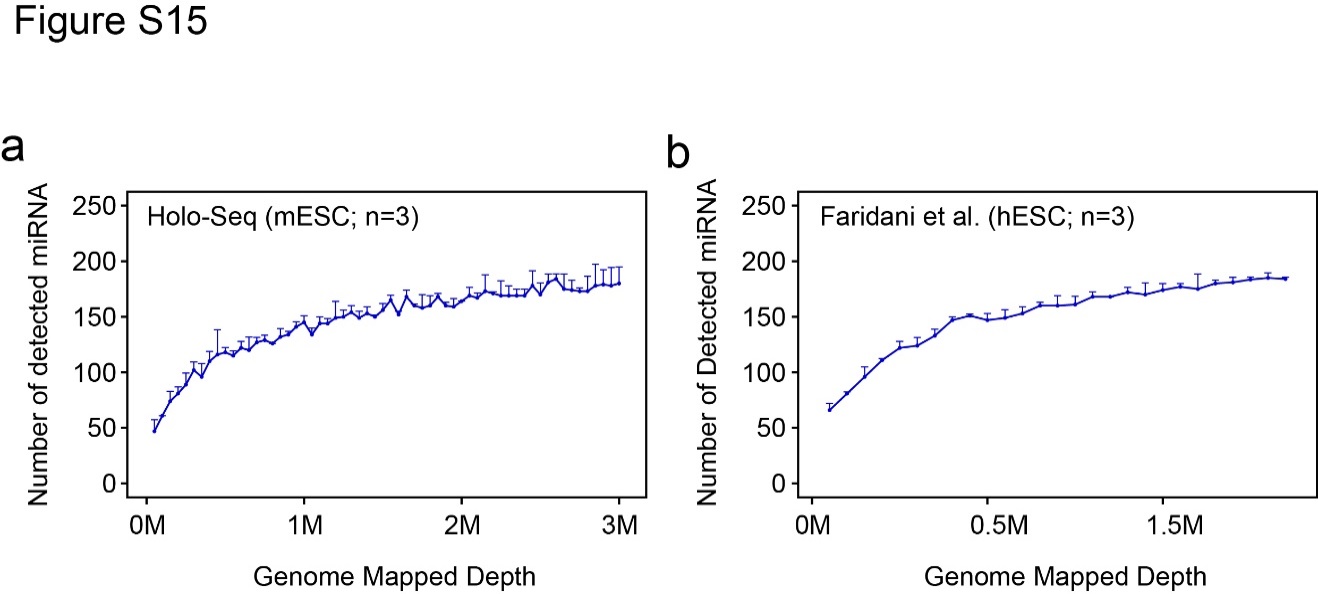


**Figure S15: The saturation curves of miRNA**

a-b, The number of detected miRNAs at different genome mapped depth of Holo-Seq small RNA libraries (mESC; n=3) and the libraries of the published single cell small RNA sequencing method [[29](#_ENREF_29)](hESC; n=3). Error bars: Median Absolute Deviation (MAD).

**
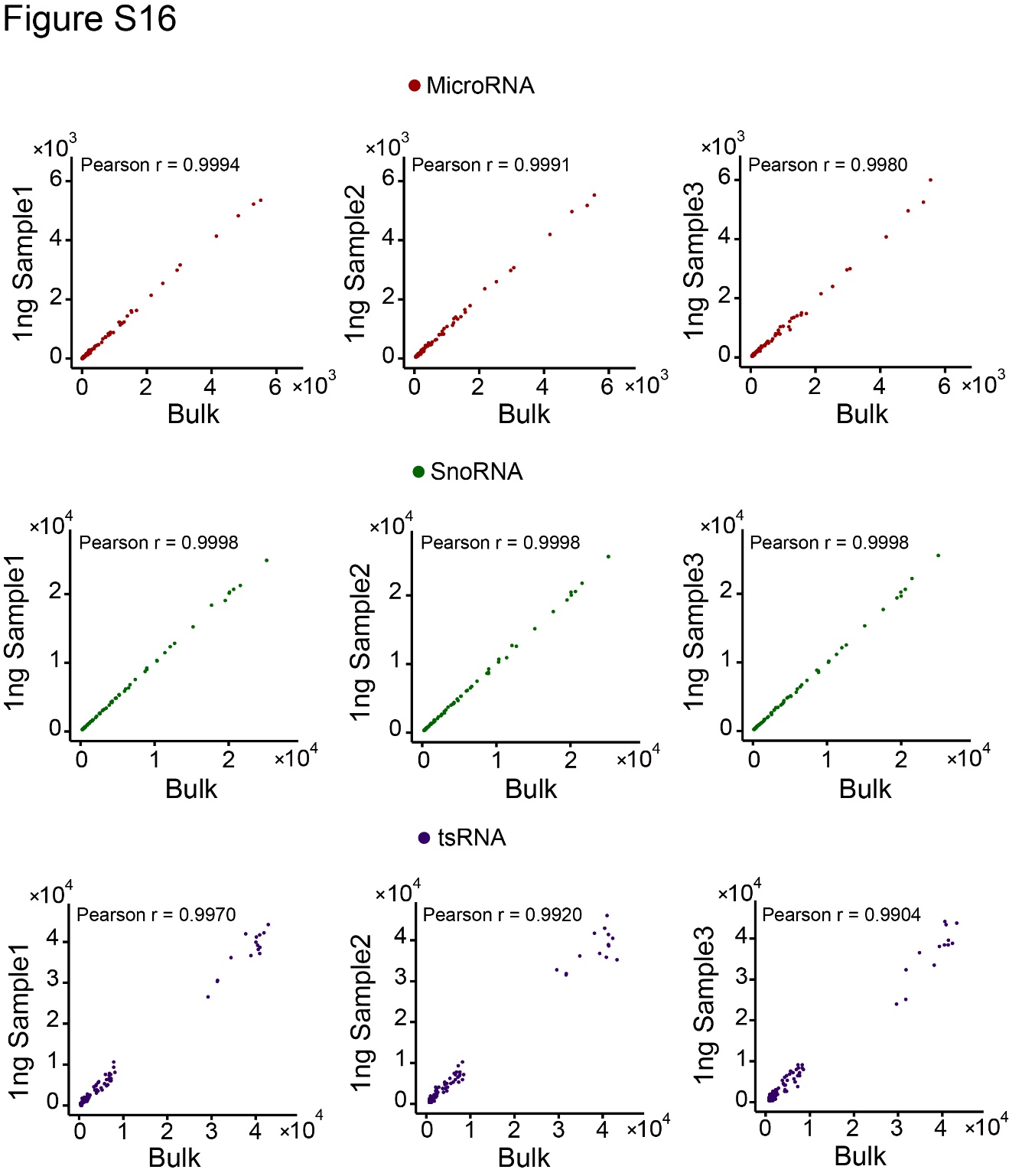
**

**Figure S16:** **RPM scatterplots of expressed small RNAs.**

The RPM scatterplots of miRNAs, snoRNAs and tsRNAs between bulk small RNA-Seq and Holo-Seq (1ng ZHBTc4 total RNA).

**
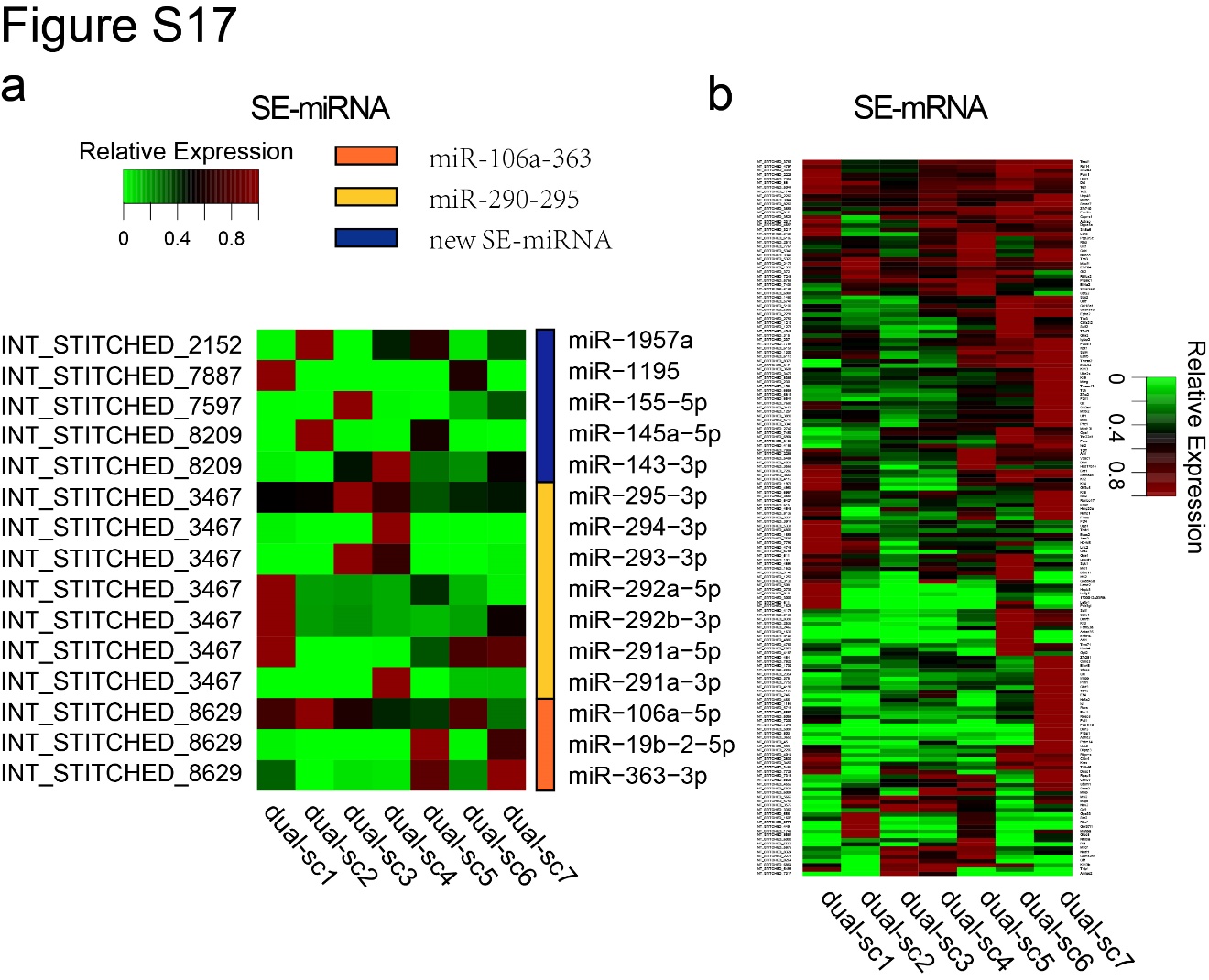
**

**Figure S17：Relative expression heat maps of super-enhancer-regulated master miRNAs and mRNAs.**

a-b, Heat maps of the relative expression of master miRNAs and mRNAs regulated by super-enhancers from seven mESC single cells.

**
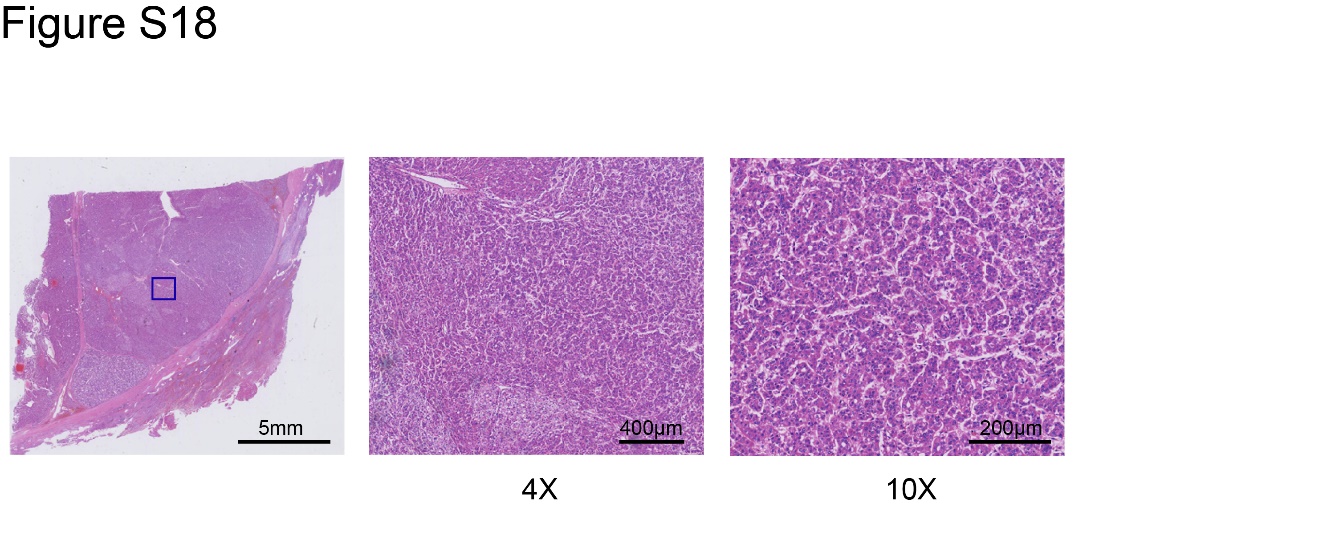
**

**Figure S18**: **Hematoxylin and Eosin (HE) staining of the HCC tissue.**

**
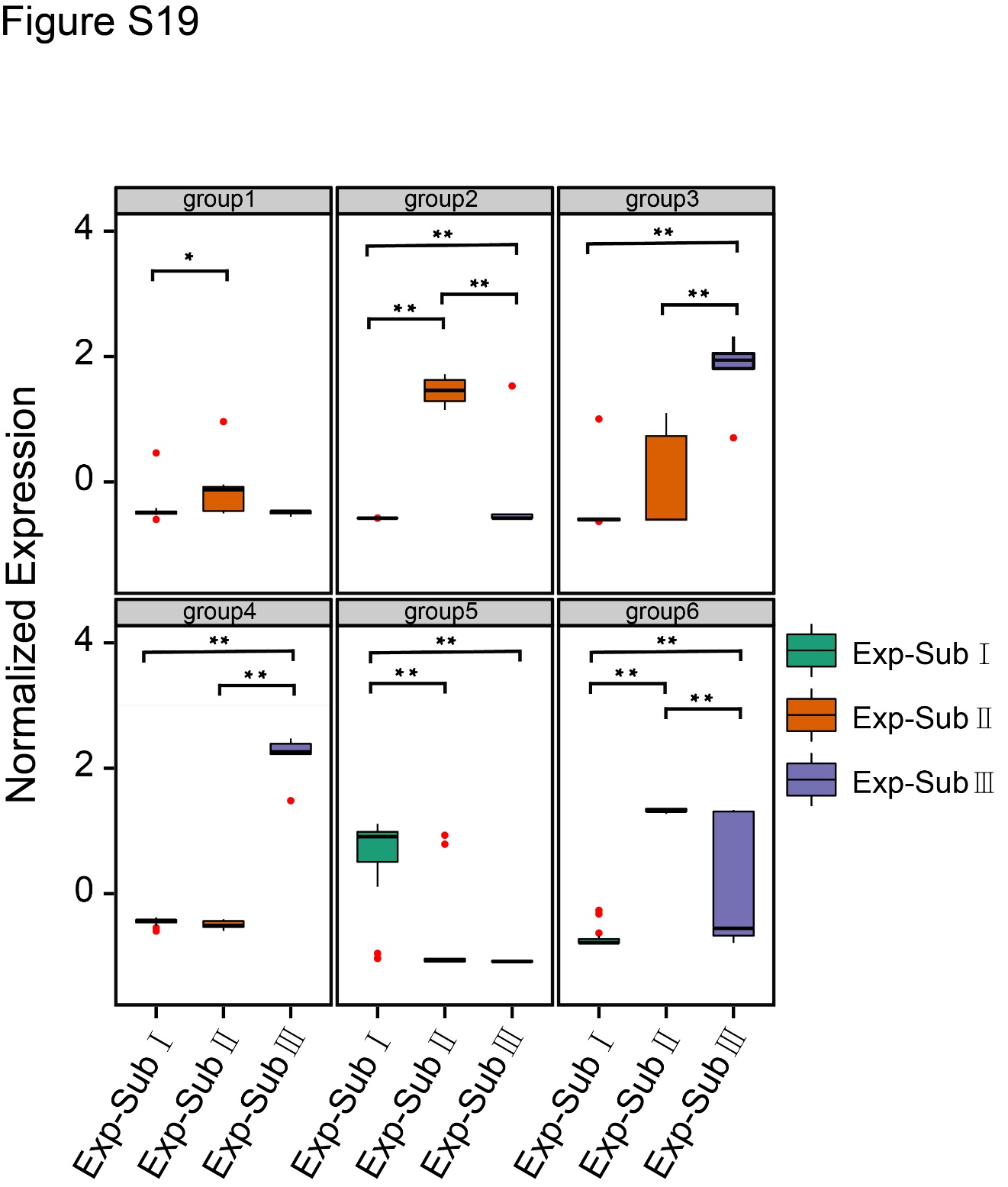
**

**Figure S19: Relative expression levels of gene groups between HCC Exp-subpopulations.**

The boxplots show the relative expression level of gene groups in three HCC Exp-subpopulations. * p<0.05 ** p < 0.01

**
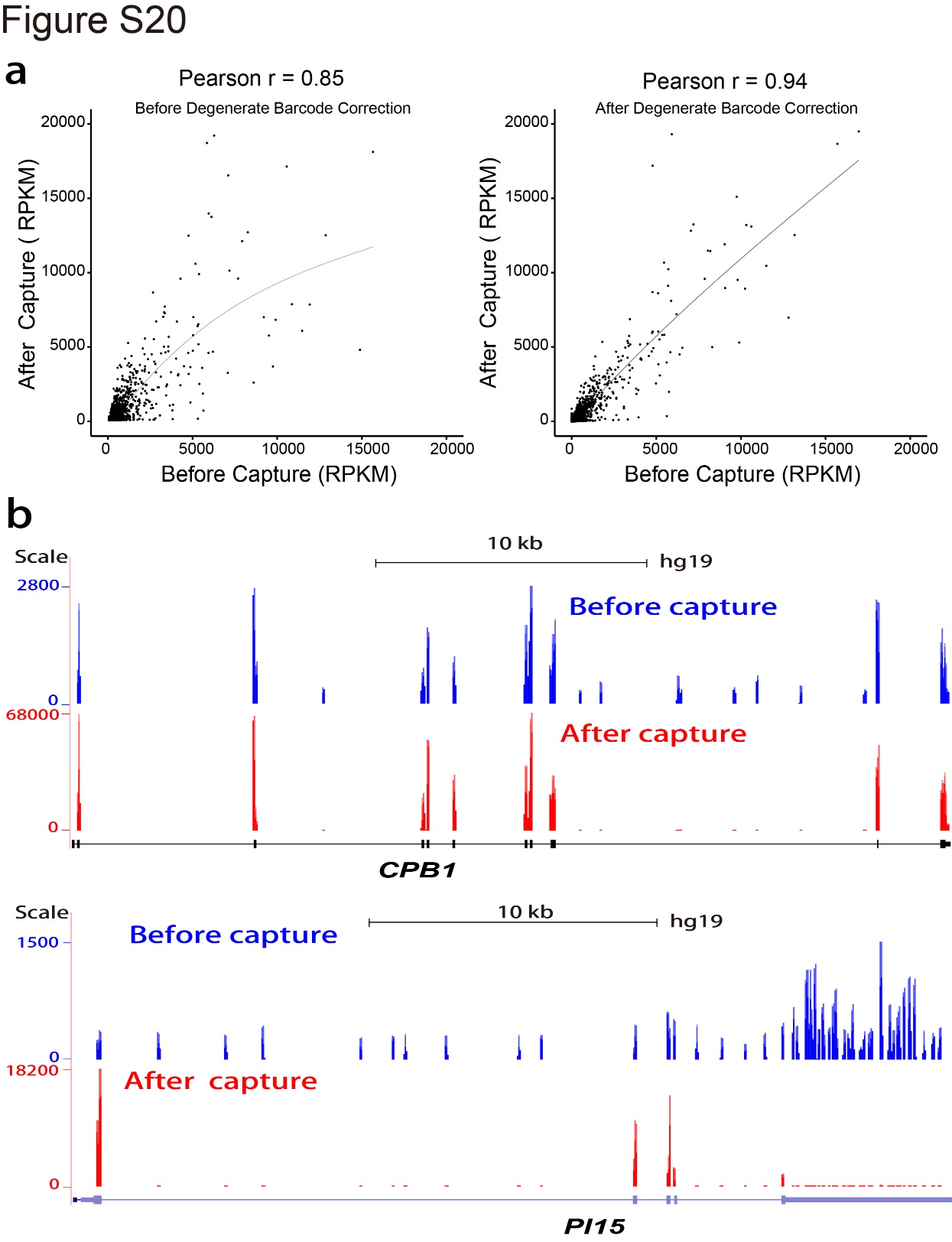
**

**Figure S20: mRNA capture sequencing of the Holo-Seq total RNA library.**

a. RPKM scatterplot of the targeted genes between original directional total RNA sequencing and captured sequencing from MCF7 cell. The PCR amplification bias is efficiently corrected by the 8-nt degenerate barcode.

b. Signal plots of the CBP1 and PI15 loci from original directional total RNA sequencing and captured sequencing from MCF7 cell.


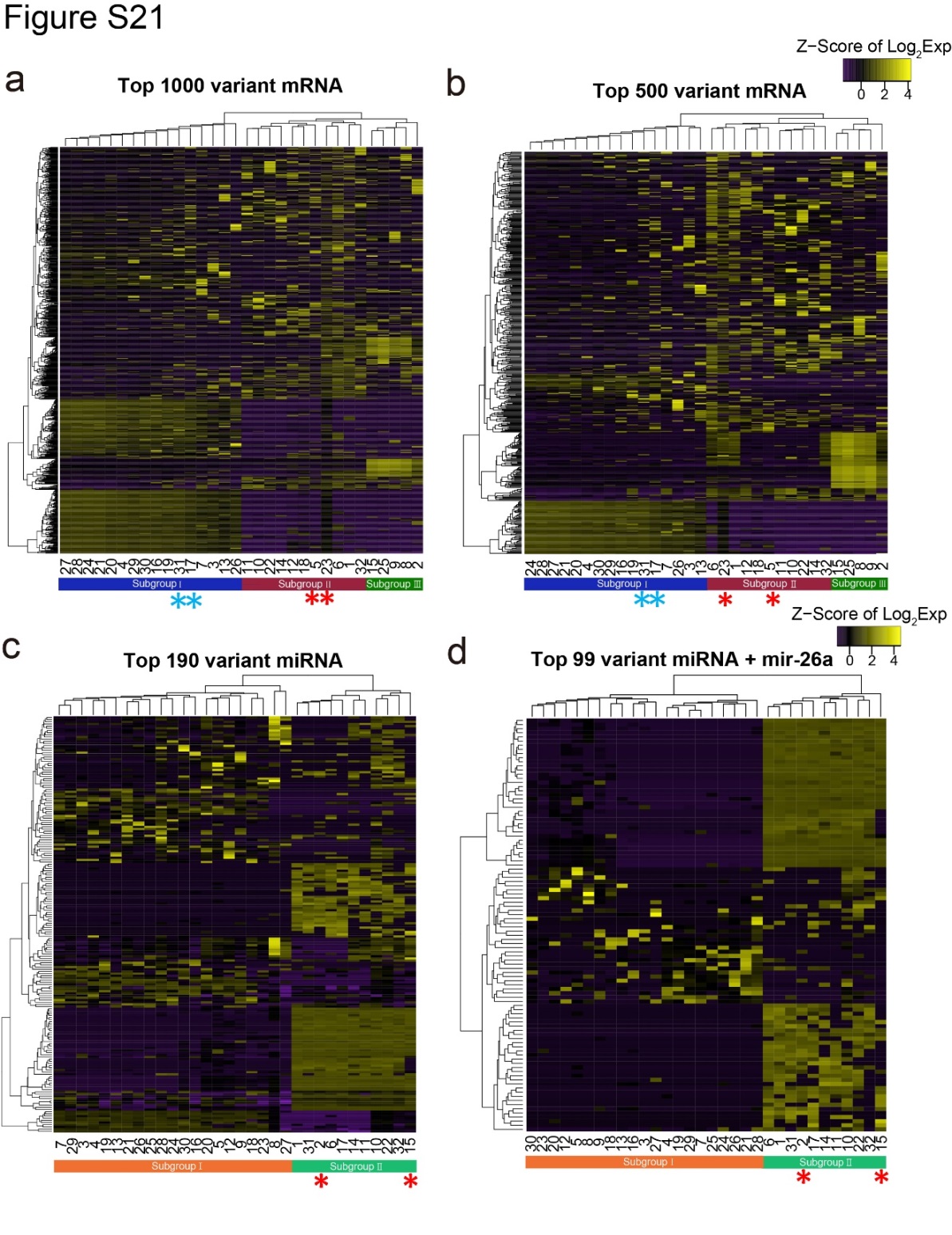


**Figure S21: mRNA and miRNA solo transcriptome analyses of hepatocellular carcinoma (HCC) single cells.**

a-b, Hierarchical clustering heatmaps by top 1000 variant mRNAs (a) and top 500 variant mRNAs (b) of 32 HCC single cells. Cells in subgroup I, II and III are similar to the cells in Exp-sub I, II and III of Figure 6. Blue * labeled cells are in Exp-sub II and Red * labeled cells are in Exp-sub I of Figure 6a.

c-d, Hierarchical clustering heatmaps by top 190 variant miRNAs (c) and top 99 variant miRNAs + mir-26a (d) of 32 HCC single cells. Cells in subgroup 1 are the mixture of Exp-sub I and III of Figure 6a. Cells in subgroup II are the cells in Exp-sub II of Figure 6 except cell-2 and cell-15 (Red * labeled).
