# Supplementary material for: Holo-Seq: single-cell sequencing of holo-transcriptome
Source: Genome Biol. 2018 Oct 17;19:163. doi: 10.1186/s13059-018-1553-7 (PMC6193298; doi:10.1186/s13059-018-1553-7)
Supplement: Supplementary file 4 — Step-by-step Holo-Seq protocols. (DOCX 29 kb) [file 13059_2018_1553_MOESM4_ESM.docx]

**Sequencing library construction of mRNAs from a single cell**

**(Step by step protocol)**

1. Cell lysis

Pipette single cell in lysis solution or in 3μL 0.1% BSA/PBS with 100 ng of the carrier RNA mixture, briefly centrifuge, put on preheated thermal cycler 95°C 5min, then quickly transfer to ice, proceed immediately to mRNA isolation.

Lysis solution:

0.2% Triton-X100 (Sigma) in Nuclease-free water 2μL

Recombinant RNase Inhibitor (Takara) 0.1μL

(The sequencing library was constructed following the manufacturer’s protocol using NEBNext kits E7490 & E7530)

1. mRNA isolation

Use NEBNext kits E7490 to isolate mRNA.

10μL NEBNext Oligo d(T)_25_ beads in 2X RNA Binding Buffer 25μL

Single cell lysate with carrier 3μL

Nuclease-free water 22μL

1. Fragmentation and Priming

Elute isolated mRNA from the beads with First-Strand Synthesis Reaction Buffer and Random Primer mix (2X) 8μL

First Strand Synthesis Reaction Buffer and Random Primer mix (2X):

NEBNext First-Strand Synthesis Reaction Buffer (5X) 4μL

NEBNext Random Primers 1μL

Nuclease-free water 5μL

94°C 15min

Hold at 4°C

1. First Strand cDNA Synthesis

Fragmented and primed mRNA 7.5μL

Murine RNase Inhibitor 0.25μL

ProtoScript II Reverse Transcriptase 0.5μL

Nuclease-free water 1.75μL

25°C 10min

42°C 60min

70°C 15min

Hold at 4°C

1. Second Strand cDNA Synthesis

First Strand Synthesis reaction 10μL

Second Strand Synthesis Reaction Buffer (10X) 4μL

Second Strand Synthesis Enzyme Mix 2μL

Nuclease-free water 24μL

16°C 60min

Hold at 4°C

1. Double-stranded cDNA purification

Purify the Double-stranded cDNA Using 1.8X Agencourt AMPure XP Beads 72μL

1. End Prep of cDNA Library

Purified double-stranded cDNA 27.75μL

NEBNext End Repair Reaction Buffer (10X) 3.25μL

NEBNext End Prep Enzyme Mix 1.5μL

20°C 30min

65°C 30min

Hold at 4°C

1. Adaptor Ligation

End Prep Reaction 32.5μL

Blunt/TA Ligase Master Mix 7.5μL

20 fold Diluted NEBNext Adaptor 1μL

Nuclease-free water 0.75μL

20°C 15min

Add 1.5μL USER Enzyme to the ligation mixture

37°C 15min

1. Purify the Ligation Reaction Using 1X AMPure XP Beads (Add 100ng ~2Kb DNA carrier)
2. Removal of Carrier with Not1 or Cas9 nuclease

Not1:

Adaptor Ligated DNA library 43μL

NEB 10X CutSmart Buffer 5μL

Not1 Enzyme 2μL

37°C 2h

Cas9 nuclease:

Adaptor Ligated DNA library 46μL

NEB 10X Cas9 nuclease Reaction Buffer 6μL

Cas9 nuclease NLS 2μL

SgRNA mix (300nM) 6μL

37°C 2h

1. Purify the Carrier-removal Reaction Using 1X AMPure XP Beads.
2. PCR Enrichment

Carrier-removal library 10μL

Index Primer/i7 Primer 1.25μL

Universal PCR Primer/i5 Primer 1.25μL

2X NEBNext Q5 Hot Start HiFi PCR Master Mix 12.5μL

98°C 30s

16 cycles of

98 °C 10s

65 °C 75s

End cycles

65 °C 5min

Hold at 4°C

1. Purify the PCR Reaction using 0.9X AMPure XP Beads

**Directional sequencing library construction of total RNAs from a single cell**

**(Step by step protocol)**

1. Cell lysis

Pipette single cell in lysis solution or in 3μL 0.1% BSA/PBS with 100 ng of the carrier RNA mixture, briefly centrifuge, put on preheated thermal cycler 95°C 5min, then quickly transfer to ice, proceed immediately to Fragmentation and Priming.

(The sequencing library was constructed following the manufacturer’s protocol using NEBNext kits E7420)

1. Fragmentation and Priming

Single cell lysate with carrier 3μL

NEBNext First-Strand Synthesis Reaction Buffer (5X) 4.8μL

NEBNext Random Primers 1.2μL

Nuclease-free water 3μL

94°C 15min

Hold at 4°C

1. First Strand cDNA Synthesis

Fragmented and primed mRNA 12μL

Murine RNase Inhibitor 0.25μL

ProtoScript II Reverse Transcriptase 0.5μL

Actinomycin D (0.25μg/μL) 1.25μL

25°C 10min

42°C 60min

70°C 15min

Hold at 4°C

1. Second Strand cDNA Synthesis

First Strand Synthesis reaction 14μL

Second Strand Synthesis Reaction Buffer (dUTP, 10X) 4μL

Second Strand Synthesis Enzyme Mix 2μL

Nuclease-free water 20μL

16°C 60min

Hold at 4°C

1. Double-stranded cDNA purification

Purify the Double-stranded cDNA Using 1.8X Agencourt AMPure XP Beads 72μL

1. End Prep of cDNA Library

Purified double-stranded cDNA 27.75μL

NEBNext End Repair Reaction Buffer (10X) 3.25μL

NEBNext End Prep Enzyme Mix 1.5μL

20°C 30min

65°C 30min

Hold at 4°C

1. Adaptor Ligation

End Prep Reaction 32.5μL

Blunt/TA Ligase Master Mix 7.5μL

20 fold Diluted NEBNext Adaptor 1μL

Nuclease-free water 0.75μL

20°C 15min

Hold at 4°C

1. Purify the Ligation Reaction Using 1X AMPure XP Beads (Add 100ng ~2Kb DNA carrier)
2. Removal of Carrier with Not1 or Cas9 nuclease

Not1:

Adaptor Ligated DNA library 43μL

NEB 10X CutSmart Buffer 5μL

Not1 Enzyme 2μL

37°C 2h

Cas9 nuclease:

Adaptor Ligated DNA library 46μL

NEB 10X Cas9 nuclease Reaction Buffer 6μL

Cas9 nuclease NLS 2μL

SgRNA mix (300nM) 6μL

37°C 2h

1. Purify the Carrier-removal Reaction Using 1X AMPure XP Beads.
2. PCR Enrichment

Carrier-removal library 10μL

Index Primer/i7 Primer 1.25μL

Universal PCR Primer/i5 Primer 1.25μL

2X NEBNext Q5 Hot Start HiFi PCR Master Mix 12.5μL

USER Enzyme 1.5μL

37°C 15min

98°C 30s

16 cycles of

98 °C 10s

65 °C 75s

End cycles

65 °C 5min

Hold at 4°C

1. Purify the PCR Reaction using 0.9X AMPure XP Beads

**Sequencing library construction of small RNAs from a single cell**

**(Step by step protocol)**

1. Cell lysis

Pipette single cell in lysis solution or in 3μL 0.1% BSA/PBS with 100 ng 5’-phosphorylated small carrier RNA mimic, briefly centrifuge, put on preheated thermal cycler 95°C 5min, then quickly transfer to ice, proceed immediately to 3’ Ligation.

(The sequencing library was constructed following the manufacturer’s protocol using NEBNext kits E7300)

1. 3’ Ligation

Single cell lysate with carrier 3μL

2 fold Diluted 3’ SR Adaptor for Illumina 0.5μL

70°C 2min, immediately transfer to ice.

Add

3´ Ligation Reaction Buffer (2X) 5μL

3´ Ligation Enzyme Mix 1.5μL

25°C 2h

1. Reverse Transcription Primer Hybridization

Add

2 fold Diluted SR RT Primer for Illumina 0.5μL

Nuclease-free water 2.25μL

75°C 5min

37°C 15min

25°C 15min

1. 5’ Ligation

Add

2 fold Diluted 5’ SR Adaptor for Illumina (denatured) 0.5μL

5’ Ligation Reaction Buffer (10X) 0.5μL

5’ Ligation Enzyme Mix 1.25μL

25°C 1h

1. Reverse Transcription

Adaptor Ligated RNA 15μL

First Strand Synthesis Reaction Buffer 4μL

Murine RNase Inhibitor 0.5μL

ProtoScript II Reverse Transcriptase 0.5μL

50°C 60min

70°C 15min

1. PCR Amplification

Reverse Transcription Reaction 20μL

LongAmp Taq 2X Master Mix 25μL

SR Primer for Illumina 1.25μL

Index (X) Primer 1.25μL

Nuclease-free water 2.5μL

94°C 30s

22 cycles of

94 °C 15s

62 °C 30s

70 °C 15s

End cycles

70 °C 5min

Hold at 4°C

1. Purify the PCR Reaction Using 1.8X AMPure XP Beads

8. Removal of Carrier with Not1 or Cas9 nuclease

Not1:

Enriched DNA library 43μL

NEB 10X CutSmart Buffer 5μL

Not1 Enzyme 2μL

37°C 2h

Cas9 nuclease:

Enriched DNA library 46μL

NEB 10X Cas9 nuclease Reaction Buffer 6μL

Cas9 nuclease NLS 2μL

SgRNA mix (300nM) 6μL

37°C 2h

1. Purify the Carrier-removal Reaction Using 1X AMPure XP Beads. (Add 100ng ~2Kb DNA carrier)

**Dual-sequencing library construction of small RNAs and poly(A) mRNAs from a single cell**

**(Step by step protocol)**

1. Cell lysis

Pipette single cell in lysis solution or in 2μL 0.1% BSA/PBS with 100 ng carrier RNA mixture and 100ng 5’-phosphorylated small carrier RNA mimic, briefly centrifuge, put on preheated thermal cycler 95°C 5min, then quickly transfer to ice, proceed immediately to mRNA isolation.

(The sequencing library was constructed following the manufacturer’s protocol using NEBNext kits E7490 & E7530 & E7300)

1. mRNA isolation

Use NEBNext kits E7490 to isolate mRNA.

10μL NEBNext Oligo d(T)_25_ beads in 2X RNA Binding Buffer 2μL

Single cell lysate with carrier 2μL

65°C 5min

Hold at 4°C

Resuspend the beads and incubate at room temperature for 10 minutes to allow the mRNA to bind to the beads.

1. mRNA and Small RNA isolation

Place the tube on the magnetic rack, transfer 3μL supernatant containing small RNAs to a clean nuclease-free PCR Tube with 0.5μL 2-fold Diluted 3’ SR Adaptor for Illumina and continue with small RNA library construction as described above.

The mRNA binding to the beads should be isolated using NEBNext kits E7490 and continue with mRNA library construction as described above.
