# Supplementary material for: Holo-Seq: single-cell sequencing of holo-transcriptome
Source: Genome Biol. 2018 Oct 17;19:163. doi: 10.1186/s13059-018-1553-7 (PMC6193298; doi:10.1186/s13059-018-1553-7)
Supplement: Supplementary file 5 — Review history. (DOCX 53 kb) [file 13059_2018_1553_MOESM5_ESM.docx]

Review History

**First round of review**

**Reviewer 1**

**Are the methods appropriate to the aims of the study, are they well described, and are necessary controls included?**
The Holo-seq method needs to be better evaluated in regard to the proportion of informative reads and cost-effectiveness.

**Are the conclusions adequately supported by the data shown?**

Neither the technical nor biological conclusions are sufficiently supported by the current analysis.

**Are sufficient details provided to allow replication and comparison with related analyses that may have been performed?**

Yes, although the authors refer to the manufacturer's instruction and a brief description of the workflow would be favourable.

**Does the work represent a significant advance over previously published studies? Please explain.**

Yes

**Is the paper of broad interest to others in the field, or of outstanding interest to a broad audience of biologists?**

The method is very valuable to a limited set of researchers.

Comments to authors:

The authors introduce a new single-cell RNAseq method termed Holo-Seq that makes use of artificial spike-in RNA to couple single-cell RNA-seq to established library preparation workflows for bulk RNA-seq. Specifically, the use of high amount of artificial RNA enabled the authors to apply NEB library preparation kits on low input material (1 ng) and single cells. Holo-seq could advance current single-cell library preparation methods by providing full-length information also for long transcripts, quantifying antisense sequences and combining information from coding transcriptomes with small RNAs. The authors provide initial proof-of-concept results and subsequently apply their method to gain insights into mESC and liver cancer biology. The manuscript tries to balance between the introduction of a new methods and its application. Here, the weight (starting with the title) should be on the methodological part to better estimate the performance of the technique. Especially, the efficiency of the method to derive informative reads (mapping on-target) needs to better presented (also in respect to the required sequencing depth and the costs). In the current form of the manuscript the performance of the technique is hard to evaluate and, on the other hand, biological insights seems not to be sufficiently supported. The authors should focus a revised manuscript on a better introduction and benchmarking of Holo-seq. 

Major comments:

For all library preparation types basic mapping statistics (exon, intron, intergenic, unmapped, etc) would help to better judge the on-target productivity of the method. In general, the weight of the manuscript should be on the description and benchmarking of the method and less on the interpretation of the results. How many reads fall into the carrier RNA? How many rRNA reads in supernatant based libraries? A more detailed description of the method in results (including main figure) and the methods section would be beneficial and better set the focus on the method itself. 

The low accuracy of Smart-Seq2 with 1ng of input RNA is a bit puzzling. I suspect that the SMARTseq (Clontech) workflow might not perform optimally with the selected NEB library preparation kit. The authors should compare their results with SMARTseq protocols coupled with Nextera XT workflow. 

What is the predicted number of endogenous transcripts cut (and removed) by NotI? In other word, is there an off-target effect of the restriction that could bias/deplete the resulting libraries?

The authors state that the Smart-Seq2 is relatively far away from the bulk in the tSNE representation. Please note that distances in the tSNE do not reflect real distances and thus this representation is not an adequate measure to draw this conclusion. 

Did the authors consider to use spike-in RNAs (ERCC) to better estimate the accuracy and sensitivity of the method compared to other techniques. In particular the benchmarking against other methods could be better supported by these measures. 

The authors sequenced their libraries to pretty high depth, likely being close to the saturation point (Fig. 1e). To better estimate the complexity of the libraries and the number of reads required to detect high number of genes the authors should down-sample (or/and simulate) both the Holo-seq and Smart-Seq2 libraries to determine the saturation point for each technique. This comparison would be also informative in the section that includes MATQ-seq and SUPeR-seq. 

The authors claim that the antisense transcript are more diversely expressed between single cells compared to mRNAs. As antisense RNAs are likely expressed to much lower level this result has to be confirmed using expression level matched coding transcripts. The following conclusion that the antisense transcript are able to separate mESC from mouse Tcell is not very surprising considering the vast difference between the cell types. 

Regarding the microRNA analysis, again, an estimate of the optimal sequencing depth would be very helpful to estimate library complexity and the amount of sequencing required to detect these numbers and to draw such conclusions. 

Some analyses (e.g. microRNA networks and super-enhancer; Figure 5) are very limited by the small number of cells that were profiled. Is this due to the high sequencing depth and the related high costs per cell? This relates to the former comment that the costs of the methods have to be evaluated in terms of sequencing required, the library complexity and the proportion of informative reads. Conclusion drawn from low cell number analysis have to be replicated or confirmed by external data sets. In the current form this analysis provides very weak evidence for the strong conclusions drawn in the manuscript. 

The authors state the following and it is unclear to which data they compared to reach the conclusion: "As expected, the miRNA and mRNA abundance profiles possessed better potency to assign the cell heterogeneity (data not shown)." The subsequent description of the expression groups is not informative if not directly related to transcript types or putative functional interpretation. 

The authors might consider down-toning their statements about tumor cell malignancy as this interpretation is solely based on the annotation of differentially expression genes/miRNA. 

The super-enhancer analysis and interpretation seems a bit off scope in this manuscript. The read out (expression of SE related transcripts) is not specific for this technique and could be achieved with other scRNA-seq methodologies. The analysis of SE does not give an added value to this manuscript or technique. Also, the related conclusions appear quite premature and are not sufficiently supported or replicated. 

The authors should discuss their method in light of pervious published work on small and non-coding single-cell RNA seq methods (doi:10.1038/nbt.3701, RamID-seq, DUAL-seq, etc).

**Reviewer 2**

**Are the methods appropriate to the aims of the study, are they well described, and are necessary controls included?**
Yes

**Are the conclusions adequately supported by the data shown?**

Yes

**Are sufficient details provided to allow replication and comparison with related analyses that may have been performed?**

Yes

**Does the work represent a significant advance over previously published studies? Please explain.**

Yes

**Is the paper of broad interest to others in the field, or of outstanding interest to a broad audience of biologists?**

Yes: Whole transcriptomics analysis of a single cell draws attention in physiological and pathologic process

Comments to author:

In this study, the authors introduced the Holo-seq method that could simultaneously measure small RNA and mRNA in a single cell. They showed the main advantages of Holo-Seq over existing methods. The authors applied Holo-seq to hepatocellular carcinoma and presented the single-cell resolution hepatic neoplasm kinetics. The authors provided an efficient method that combines mRNA and miRNA to investigate cell or tumor heterogeneity. Some specific concerns and suggestions are listed as followings:

Major Concerns:

1.	The authors used carrier that contains Not I site in the library construction. The original genome also contains Not I sites. Could the digestion of carrier affect the original genome? Will some of the genomic fragments containing Not I sites be missing in the sequencing results?

2.	In Figure 3e, "the pre-mRNA bursting of some housekeeping genes was significantly higher than that of some core transcriptional factors". What are "some housekeeping genes" and "some core transcriptional factors"? Do their differences have biological meanings in the specific cells?

3.	In Figure 4c, "the miRNA and tsRNA abundance profiles possessed better potency to assign cell heterogeneity than the snoRNA abundance profile", could the "potency" be calculated? Or just judged from the figures?

4.	In "miRNA-mRNA regulatory network in mouse ES cells" part, 61 miRNA-target pairs were selected from 695 miRNA-target pairs. That's no more than 10 percent, which means the majority of miRNA-target pairs are not anti-correlated in the data. Could the authors please explain this observation?

5.	In Figure 6a, cells were clustered based on a combined expression profiles of both mRNAs and miRNAs. Did the authors compare the results generated by using mRNAs or miRNAs alone with the current results?

Minor Concerns:

1.	In Figure 6a, when selecting featured mRNAs and miRNAs, the authors chose top 500 mRNA and top 99 miRNA according to the variation rank. Why do the authors choose the cut-off "500" and "99"?

2.	In Figure 7a, are all the mRNA targets tumor suppressors or oncogenes? Please mark them in different colors

**Authors’ response to reviewers:**

Reviewer #1: The authors introduce a new single-cell RNAseq method termed Holo-Seq that makes use of artificial spike-in RNA to couple single-cell RNA-seq to established library preparation workflows for bulk RNA-seq. Specifically, the use of high amount of artificial RNA enabled the authors to apply NEB library preparation kits on low input material (1 ng) and single cells. Holo-seq could advance current single-cell library preparation methods by providing full-length information also for long transcripts, quantifying antisense sequences and combining information from coding transcriptomes with small RNAs. The authors provide initial proof-of-concept results and subsequently apply their method to gain insights into mESC and liver cancer biology. The manuscript tries to balance between the introduction of a new methods and its application. Here, the weight (starting with the title) should be on the methodological part to better estimate the performance of the technique. *(Thanks for the suggestion! We have revised the manuscript accordingly.)*

Especially, the efficiency of the method to derive informative reads (mapping on-target) needs to better presented (also in respect to the required sequencing depth and the costs). In the current form of the manuscript the performance of the technique is hard to evaluate (We have added such information in the revised manuscript (Table1-sheet 3). The cost of Holo-seq for mRNA (~$30) is significantly lower than Smart-seq2 (~$110). Because no significant expensive commercial reagents and high sequencing depth required, the cost of Holo-seq for total RNA (~$50) and small RNA ($32.5) should be comparable with previously published approaches (MATQ-seq, SUPeR-seq and single cell small RNA-seq). The detailed cost measurement is in (Table1-sheet4).) and, on the other hand, biological insights seems not to be sufficiently supported. The authors should focus a revised manuscript on a better introduction and benchmarking of Holo-seq. *(We have revised the manuscript accordingly and toned down many statements.)*

Major comments:

For all library preparation types basic mapping statistics (exon, intron, intergenic, unmapped, etc) would help to better judge the on-target productivity of the method. *(We have added the mapping information of all 120 single-cell sequencing libraries in the (Table1-sheet 3).)* In general, the weight of the manuscript should be on the description and benchmarking of the method and less on the interpretation of the results *(Thanks for the suggestion! We have modified the revised manuscript accordingly. We have added several new data (such as saturation analysis, Not I digestion analysis and clustering analysis, etc.) on benchmarking and detailed step-by-step protocols to describe Holo-seq.).* How many reads fall into the carrier RNA? *(There are around 10%--20% carrier-mapped reads (Table1-sheet 3).)* How many rRNA reads in supernatant based libraries? *(There are 50%--60% rRNA-mapped reads which has been described in the discussion section of the original manuscript. We have added this information in the result section of the revised manuscript. We also provided a whole-exome capture strategy which can efficiently remove the rRNA reads (>90%; Figure S20) when necessary.)* A more detailed description of the method in results (including main figure) and the methods section would be beneficial and better set the focus on the method itself *(Thanks for the suggestion！We think that the method section is a good place to add more detailed description. We have added all step-by-step protocols of Holo-seq in the method section.).*

The low accuracy of Smart-Seq2 with 1ng of input RNA is a bit puzzling. I suspect that the SMARTseq (Clontech) workflow might not perform optimally with the selected NEB library preparation kit. The authors should compare their results with SMARTseq protocols coupled with Nextera XT workflow *(We performed SMARTseq protocol coupled with Nextera XT workflow and found that it does not improve the accuracy of Smart-Seq2 (Figure S7). We believe that the preamplification of full-length cDNA fragments which have various lengths and secondary structures lowers the accuracy of Smart-Seq2. So, optimizing the library construction step which is after the preamplification step would not improve the accuracy of SMARTseq).*

What is the predicted number of endogenous transcripts cut (and removed) by NotI? In other word, is there an off-target effect of the restriction that could bias/deplete the resulting libraries? *(There are 2671 Not1-site-containing genes in human and 1679 Not1-site-containing genes in mouse (Table 1-sheet 1,2).* *Most of them (>90%) contain < 3 cutting sites. Not I digestion only disrupts very limited reads of a Not1-site-containing transcript and cannot deplete the whole transcript from a library because Holo-Seq fragments mRNAs to generate short cDNAs (200-300bp) before the library construction step. This only causes a very limited bias, which is supported by the high correlation (r=0.985-0.987) of Not1-site-containing transcripts between Not I-digested libraries and normal libraries (Figure S3 a,b). We did not find any notable off-target effects through comparing Not1-digested libraries with normal libraries (Figure S4 a,b)).*

*As we have discussed in the original manuscript, the carrier can also be removed by CRISPR/Cas9 digestion if the restriction enzyme was not accepted. Theoretically, the CRISPR/Cas9 digestion unlikely bias or deplete any known transcripts, and we did not find any notable off-target effects through comparing Cas9-digested libraries and normal libraries (Figure S3 c,d))*

The authors state that the Smart-Seq2 is relatively far away from the bulk in the tSNE representation. Please note that distances in the tSNE do not reflect real distances and thus this representation is not an adequate measure to draw this conclusion*. (Thanks for the suggestion! We have revised the sentence into “, whereas the data of Smart-Seq2 (1ng) and Smart-Seq2 (SC) are separated from them.”, and we also added a clustering analysis to support this conclusion (Figure S6).)*

Did the authors consider to use spike-in RNAs (ERCC) to better estimate the accuracy and sensitivity of the method compared to other techniques. In particular the benchmarking against other methods could be better supported by these measures. *(Because many single-cell RNA-Seq studies used ERCC to perform quality controls (Fan et al., 2015; Islam et al., 2014; Macosko et al., 2015; Sheng et al., 2017), we do plan to use ERCC to estimate the accuracy and sensitivity at the beginning. However, we found that the size of spike-in RNAs (ERCC) is short (250nt to 2000nt; Ambion Cat: 4456740) and Smart-seq/other methods can accurately probe short (250nt to 2000nt) but not long transcripts (Figure 1f). In addition, several other papers have similar observations that ERCC cannot represent the complexity of mammalian transcriptome (Sheng et al., 2017; Vallejos et al., 2017). So spike-in RNAs (ERCC) is not an ideal benchmark to compare Holo-seq with other methods.)*

The authors sequenced their libraries to pretty high depth, likely being close to the saturation point (Fig. 1e). To better estimate the complexity of the libraries and the number of reads required to detect high number of genes the authors should down-sample (or/and simulate) both the Holo-seq and Smart-Seq2 libraries to determine the saturation point for each technique. This comparison would be also informative in the section that includes MATQ-seq and SUPeR-seq *(This is a very good point! We have simulated the detected gene number at different unique exome-mapped reads of Holo-Seq and other methods (Smart-Seq2, MATQ-seq and SUPeR-seq). We found that only Holo-seq for mRNA and Smart-seq2 are sequenced close to the saturation point (Figure S8).*

*The detected gene number of Holo-seq increases until unique exome-mapped reads are saturated. Normally, 0.8-1 M unique exome-mapped reads (~1.5 M total reads) are the saturation region of Holo-seq mRNA libraries.*

*The complexity is measured by the number of unique exome-mapped reads which is decided by the unique broken patterns of cDNA during the fragmentation step. The high complexity of Smart-seq is artificial because it preamplifies large cDNA before the fragmentation step that leads to more broken patterns of cDNAs. Although Smart-seq2 has a higher saturated point than Holo-seq (Figure S8 a,b,c), it has lower detected gene number than Holo-seq at the same unique exome-mapped depth (Figure S8 a,b,c).*

*For total RNAs, SUPeR-seq detected fewer genes than Holo-seq at the same unique exome-mapped depth. MATQ-seq detected more genes than Holo-seq at the same unique exome-mapped depth (Figure S8 d). Although both SUPeR-Seq and MATQ-Seq preamplify the large cDNAs before the fragmentation step, we believe that the advantage of the special preamplification strategy of MATQ-seq promotes its performance (Figure S8 d).*

*Normally, 0.5 M unique exome-mapped reads (5 M total reads) are enough for Holo-seq to detect > 10000 genes from directional total RNA libraries.)*

The authors claim that the antisense transcript are more diversely expressed between single cells compared to mRNAs. As antisense RNAs are likely expressed to much lower level this result has to be confirmed using expression level matched coding transcripts. *(Thanks for the suggestion! We compared the diversity of antisense transcripts and coding transcripts which have similar expression level (Figure S12). The result still shows that the antisense transcripts are more diversely expressed between single cells compared to coding transcripts (Figure S12).)* The following conclusion that the antisense transcript are able to separate mESC from mouse Tcell is not very surprising considering the vast difference between the cell types. *(We agree that this is not very surprising. This is why antisense transcripts are also valuable to separate cell types. So, as the only method which can acquire a complete strand of origin information and efficiently probe antisense transcripts of single cells, Holo-seq is valuable to the field.)*

Regarding the microRNA analysis, again, an estimate of the optimal sequencing depth would be very helpful to estimate library complexity and the amount of sequencing required to detect these numbers and to draw such conclusions. *(We plotted the mapped reads and the number of detected miRNAs in Figure S15, which indicates that 2-3 M reads (Table1-sheet 3) is ideal sequencing depth and it is comparable to the published single-cell small RNA-seq approach (Figure S15) (Faridani et al., 2016).)*

Some analyses (e.g. microRNA networks and super-enhancer; Figure 5) are very limited by the small number of cells that were profiled. Is this due to the high sequencing depth and the related high costs per cell? This relates to the former comment that the costs of the methods have to be evaluated in terms of sequencing required, the library complexity and the proportion of informative reads. Conclusion drawn from low cell number analysis have to be replicated or confirmed by external data sets. In the current form this analysis provides very weak evidence for the strong conclusions drawn in the manuscript. *(As the data shown in the above questions, the cost is not a problem (Table 1-sheet 3,4). The purpose of Figure 5a,b is to show that Holo-seq can simultaneously profile small RNAs and mRNAs. We agree that the miRNA networks analysis from three single cells is over-digging. In the revised manuscript, we added more single cells (Figure 5). Actually, all miRNA targets are extracted from an external experiment verified database (Chou et al., 2018). The miRNA networks displayed in Figure 5a are the miRNA/mRNA pairs whose expressions are anti-correlated among all single cells supported by our data. Besides, we also toned down the statements in this part to fit our data.)*

The authors state the following and it is unclear to which data they compared to reach the conclusion: "As expected, the miRNA and mRNA abundance profiles possessed better potency to assign the cell heterogeneity (data not shown)." *(Sorry for the unclear statement! What we want to express is that “The previous report has shown that miRNA and mRNA profiles possessed the better capability to assign single cell heterogeneity (Faridani et al., 2016)” We have modified the sentence in the revised manuscript accordingly.) The subsequent description of the expression groups is not informative if not directly related to transcript types or putative functional interpretation. (There is much information in this part. Firstly, we described the patterns of expression group in different cell subpopulations; next, we described the biological function of each expression groups; finally, we inferred the biological significance of each cell subpopulation. So, we do describe the transcript types and biological functions of all expression groups in the original manuscript.)*

The authors might consider down-toning their statements about tumor cell malignancy as this interpretation is solely based on the annotation of differentially expression genes/miRNA. *(Thanks for the suggestion! We have toned down our statements in several places)*

The super-enhancer analysis and interpretation seems a bit off scope in this manuscript. The read out (expression of SE related transcripts) is not specific for this technique and could be achieved with other scRNA-seq methodologies. The analysis of SE does not give an added value to this manuscript or technique*. (We believe that a proof of principle novel application is in the scope of a method paper. A previous report has shown that miRNAs are very important effectors of super-enhancers (Suzuki et al., 2017). So, miRNA/mRNA dual information is much better than either mRNA or miRNA alone to display the super-enhancer activity. As the only scRNA-seq method which can profile miRNA and mRNA transcriptomes simultaneously, the super-enhancer analysis not only shows the specific advantage of Holo-seq but also gives an added value to the manuscript.)* Also, the related conclusions appear quite premature and are not sufficiently supported or replicated. *(We agree that some conclusions in the original manuscript are premature. We have toned down those statements to fit a method paper. We are doing a cohort study to systemically dissect the dynamics of miRNA/mRNA and super-enhancers during human hepatocellular carcinoma (HCC), which should belong to another research article and be out of the scope of this method paper)*

The authors should discuss their method in light of pervious published work on small and non-coding single-cell RNA seq methods (doi:10.1038/nbt.3701), *(We do cite and discuss this paper in many places in the original manuscript. Now, we have added more discussions.),* RamID-seq *(Sorry, we cannot find this method. What we found are DamID-seq and RamDA-seq. DamID-seq is an approach to probe protein/DNA interactions (van Steensel and Henikoff, 2000).* *RamDA-seq develops an approach to amply cDNA during reverse transcription. RamDA can probe total RNAs from single cells. However, it still cannot provide a complete strand of origin information which is the key information for non-coding RNA study (Hayashi et al., 2018). We have discussed RamDA-seq in the revised manuscript.), DUAL-seq, etc. (The DUAL-seq is a bulk total RNA-seq strategy to probe RNAs (from both host cell and pathogen) from pathogen-infected mammalian cells (Westermann et al., 2016). DUAL-seq is not for probing either small RNAs or non-coding RNAs from a single cell.*

*Holo-seq is an open platform which can also adopt the amplification strategy of Smart-seq, MATQ-seq, SUePR-seq and RamDA-seq when necessary.)*

Reviewer #2: In this study, the authors introduced the Holo-seq method that could simultaneously measure small RNA and mRNA in a single cell. They showed the main advantages of Holo-Seq over existing methods. The authors applied Holo-seq to hepatocellular carcinoma and presented the single-cell resolution hepatic neoplasm kinetics. The authors provided an efficient method that combines mRNA and miRNA to investigate cell or tumor heterogeneity. Some specific concerns and suggestions are listed as followings:

Major Concerns:

1. The authors used carrier that contains Not I site in the library construction. The original genome also contains Not I sites. Could the digestion of carrier affect the original genome? Will some of the genomic fragments containing Not I sites be missing in the sequencing results *(Holo-seq is a single cell RNA-seq not genomic DNA sequencing approach. We think what the reviewer wants to know is how Not I digestion affects the transcripts in vitro that is also asked by another reviewer.*

*Our answer is that: There are 2671 Not1-site-containing genes in human and 1679 Not1-site-containing genes in mouse (Table 1-sheet 1, 2). Most of them (>90%) contain < 3 cutting sites. NotI digestion only disrupts very limited reads of a Not1-site-containing transcript and cannot deplete the whole transcript from a library because Holo-Seq fragments mRNAs to generate short cDNAs (200-300bp) before the library construction step. This only causes a very limited bias, which is supported by the high correlation (r=0.985-0.987) of NotI-site-containing transcripts between Not I-digested libraries and normal libraries (Figure S3 a,b). We did not find any notable off-target effects through comparing NotI-digested libraries with normal libraries (Figure S4 a,b).*

*As we have discussed in the original manuscript, the carrier can also be removed by CRISPR/Cas9 digestion if the restriction enzyme was not accepted. Theoretically, the CRISPR/Cas9 digestion unlikely bias or deplete any known transcripts, and we did not find any notable off-target effects through comparing Cas9-digested libraries and normal libraries (Figure S3 c,d).)*

2. In Figure 3e, "the pre-mRNA bursting of some housekeeping genes was significantly higher than that of some core transcriptional factors". What are "some housekeeping genes" and "some core transcriptional factors"? Do their differences have biological meanings in the specific cells*? (Sorry for the unclear statement! Housekeeping genes are required for the maintenance of basic cellular function and express in all cells (Eisenberg and Levanon, 2013). Core transcriptional factors are genes that maintain the pluripotency of mESCs. Although core genes are essential for maintaining mESCs, they are rarely essential for the survival of other cells (Boyer et al., 2005). “Some” means the genes highlighted in figure 3e were randomly picked.)*

3. In Figure 4c, "the miRNA and tsRNA abundance profiles possessed better potency to assign cell heterogeneity than the snoRNA abundance profile", could the "potency" be calculated? Or just judged from the figures*? (Sorry for the unclear statement! What we want to express is that “Our results show that the miRNA and tsRNA abundance is more diverse than snoRNAs abundance, and this is consistent with the previous report that shows miRNA and tsRNA profile possess the better capability to display single cell heterogeneity (the figure1，S8 of Faridani et al., 2016).” We have modified the sentence in the revised manuscript.)*

4. In "miRNA-mRNA regulatory network in mouse ES cells" part, 61 miRNA-target pairs were selected from 695 miRNA-target pairs. That's no more than 10 percent, which means the majority of miRNA-target pairs are not anti-correlated in the data. Could the authors please explain this observation*? (The 695 miRNA-target pairs are extracted from the database (Chou et al., 2018). They are accumulated from different kinds of cells/tissues and from different kinds of experiments (most of them are in vitro experiments). It is reasonable that most of them do not exist in a specific in vivo context such as mESCs. Another possible explanation is that most negative regulations are stable in a cell type (mESCs) and cannot be detected if miRNAs do not significantly fluctuate among single cells of this cell type (mESCs). What we found are the miRNA/mRNA pairs whose expression are anti-correlated among single cells of mESCs. In the revised manuscript, we added more single cells to perform miRNA networks analysis of mESCs. Under a more stringent condition (more single cells), we found fewer anti-correlation pairs among all single cells (Figure 5a).)*

5. In Figure 6a, cells were clustered based on a combined expression profiles of both mRNAs and miRNAs. Did the authors compare the results generated by using mRNAs or miRNAs alone with the current results? *(mRNA profile alone can also cluster human hepatocellular carcinoma (HCC) cells into three similar groups with a minimal variance (four cells from Exp-sub1 and Exp-sub 2 rearranged) (Figure 6a; S21 a,b). However, without miRNA information, the biological significance of the Subgroup II is hard to explain (Figure S21 a,b). miRNA profile alone can only cluster HCC cells into two groups (Figure S21 c,d). The subgroup II cells are similar to the Exp-sub2 cells based on combined expression profiles with a minimal variance (two cells are from Exp-sub3) (Figure 6a; S21c,d). The subgroup I cells are the mixture of Exp-sub1 and Exp-sub 3 based on combined expression profiles (Figure 6a; S21c,d). Without mRNA information, the biological significance of both miRNA subgroups is also hard to explain (Figure S21c,d). We have added this information into the discussion section in the revised manuscript.)*

Minor Concerns:

1. In Figure 6a, when selecting featured mRNAs and miRNAs, the authors chose top 500 mRNA and top 99 miRNA according to the variation rank. Why do the authors choose the cut-off "500" and "99")? *(This is a good question! Currently, it is still difficult to decide how many genes should be used to perform clustering analysis in a single cell study. Normally, single cells will be clustered into too many sub-groups without clear biological significance if too many genes are used for clustering. Previous studies show that approximately 500 top variant genes can efficiently display the clustered results of single cells including liver cancer cells (Hou et al., 2016) (Matcovitch-Natan et al., 2016; Villani et al., 2017). We just selected the top 500 variant mRNAs which produce a similar result as top 1000 variant genes (Figure S21).*

*There are 190 significant variant miRNAs (Z-score<1). The clustering results by all 190 miRNA and top 100 variant miRNAs are similar, and they all can get two cell groups (Figure S21c,d). Because there are many miRNAs which did not contribute to the clustering when using all 190 miRNAs (Figure S21c,d), we just used top 99 variant miRNA+miRNA26 (a well-known tumor suppressor in liver cancer (Ji et al., 2009)).*

*What we did are simple empirical strategies and work for this method paper in which there are not a lot of single cells. In a future single-cell study of liver cancer cohorts, more efforts should be applied to optimize the clustering parameters.)*

2. In Figure 7a, are all the mRNA targets tumor suppressors or oncogenes? Please mark them in different colors. *(In figure 7a, most mRNA targets are known tumor suppressors or oncogenes. We have marked them in different colors in the revised manuscript. Thanks for the suggestion!)*

References:

Boyer, L.A., Lee, T.I., Cole, M.F., Johnstone, S.E., Levine, S.S., Zucker, J.P., Guenther, M.G., Kumar, R.M., Murray, H.L., Jenner, R.G., et al. (2005). Core transcriptional regulatory circuitry in human embryonic stem cells. Cell 122, 947-956.

Chou, C.H., Shrestha, S., Yang, C.D., Chang, N.W., Lin, Y.L., Liao, K.W., Huang, W.C., Sun, T.H., Tu, S.J., Lee, W.H., et al. (2018). miRTarBase update 2018: a resource for experimentally validated microRNA-target interactions. Nucleic Acids Res 46, D296-D302.

Eisenberg, E., and Levanon, E.Y. (2013). Human housekeeping genes, revisited. Trends Genet 29, 569-574.

Fan, X., Zhang, X., Wu, X., Guo, H., Hu, Y., Tang, F., and Huang, Y. (2015). Single-cell RNA-seq transcriptome analysis of linear and circular RNAs in mouse preimplantation embryos. Genome Biol 16, 148.

Faridani, O.R., Abdullayev, I., Hagemann-Jensen, M., Schell, J.P., Lanner, F., and Sandberg, R. (2016). Single-cell sequencing of the small-RNA transcriptome. Nat Biotechnol 34, 1264-1266.

Hayashi, T., Ozaki, H., Sasagawa, Y., Umeda, M., Danno, H., and Nikaido, I. (2018). Single-cell full-length total RNA sequencing uncovers dynamics of recursive splicing and enhancer RNAs. Nat Commun 9, 619.

Hou, Y., Guo, H., Cao, C., Li, X., Hu, B., Zhu, P., Wu, X., Wen, L., Tang, F., Huang, Y., et al. (2016). Single-cell triple omics sequencing reveals genetic, epigenetic, and transcriptomic heterogeneity in hepatocellular carcinomas. Cell Res 26, 304-319.

Islam, S., Zeisel, A., Joost, S., La Manno, G., Zajac, P., Kasper, M., Lonnerberg, P., and Linnarsson, S. (2014). Quantitative single-cell RNA-seq with unique molecular identifiers. Nat Methods 11, 163-166.

Ji, J., Shi, J., Budhu, A., Yu, Z., Forgues, M., Roessler, S., Ambs, S., Chen, Y., Meltzer, P.S., Croce, C.M., et al. (2009). MicroRNA expression, survival, and response to interferon in liver cancer. N Engl J Med 361, 1437-1447.

Macosko, E.Z., Basu, A., Satija, R., Nemesh, J., Shekhar, K., Goldman, M., Tirosh, I., Bialas, A.R., Kamitaki, N., Martersteck, E.M., et al. (2015). Highly Parallel Genome-wide Expression Profiling of Individual Cells Using Nanoliter Droplets. Cell 161, 1202-1214.

Matcovitch-Natan, O., Winter, D.R., Giladi, A., Vargas Aguilar, S., Spinrad, A., Sarrazin, S., Ben-Yehuda, H., David, E., Zelada Gonzalez, F., Perrin, P., et al. (2016). Microglia development follows a stepwise program to regulate brain homeostasis. Science 353, aad8670.

Sheng, K., Cao, W., Niu, Y., Deng, Q., and Zong, C. (2017). Effective detection of variation in single-cell transcriptomes using MATQ-seq. Nat Methods 14, 267-270.

Suzuki, H.I., Young, R.A., and Sharp, P.A. (2017). Super-Enhancer-Mediated RNA Processing Revealed by Integrative MicroRNA Network Analysis. Cell 168, 1000-1014 e1015.

Vallejos, C.A., Risso, D., Scialdone, A., Dudoit, S., and Marioni, J.C. (2017). Normalizing single-cell RNA sequencing data: challenges and opportunities. Nat Methods 14, 565-571.

van Steensel, B., and Henikoff, S. (2000). Identification of in vivo DNA targets of chromatin proteins using tethered dam methyltransferase. Nat Biotechnol 18, 424-428.

Villani, A.C., Satija, R., Reynolds, G., Sarkizova, S., Shekhar, K., Fletcher, J., Griesbeck, M., Butler, A., Zheng, S., Lazo, S., et al. (2017). Single-cell RNA-seq reveals new types of human blood dendritic cells, monocytes, and progenitors. Science 356.

Westermann, A.J., Forstner, K.U., Amman, F., Barquist, L., Chao, Y., Schulte, L.N., Muller, L., Reinhardt, R., Stadler, P.F., and Vogel, J. (2016). Dual RNA-seq unveils noncoding RNA functions in host-pathogen interactions. Nature 529, 496-501.

**Second round of review**

**Reviewer 1:**

All my comments were sufficiently addressed.

Please remove the cost estimates for the benchmarked methods (Smart-seq2, etc), as the costs differ dramatically between labs (down to 2-10 USD/cell using the reagents of the original article and low-volume pipetting). Thus, the high costs stated in the current manuscript might be give the false impression that HOLO-seq is a rather cheap method.

**Reviewer 2:**

The author has addressed all my questions and confusions.
